# Synthesis of Natural *O*-Linked Carba-Disaccharides, (+)- and (−)-Pericosine E, and Their Analogues as α-Glucosidase Inhibitors

**DOI:** 10.3390/md15010022

**Published:** 2017-01-23

**Authors:** Yoshihide Usami, Koji Mizuki, Rikiya Kawahata, Makio Shibano, Atsuko Sekine, Hiroki Yoneyama, Shinya Harusawa

**Affiliations:** 1 Laboratory of Pharmaceutical Organic Chemistry, Osaka University of Pharmaceutical Sciences, Nasahara 4-20-1, Takatsuki, Osaka 569-1094, Japan; d12007@gly.oups.ac.jp (K.M.); katahaba4@icloud.com (R.K.); SekineA@mbox.pref.osaka.lg.jp (A.S.); yoneyama@gly.oups.ac.jp (H.Y.); harusawa@gly.oups.ac.jp (S.H.); 2 Laboratory of Pharmacognosy, Osaka University of Pharmaceutical Sciences, Nasahara 4-20-1, Takatsuki, Osaka 569-1094, Japan; shibano@gly.oups.ac.jp

**Keywords:** synthesis, pericosine E, marine natural product, *O*-linked carba-disaccharide, α-glucosidase inhibitor, enantiomeric mixture

## Abstract

Pericosine E (**6**), a metabolite of *Periconia byssoides* OUPS-N133 was originally isolated from the sea hare *Aplysia kurodai*, which exists as an enantiomeric mixture in nature. The enantiospecific syntheses of both enantiomers of *Periconia byssoides* OUPS-N133 has been achieved, along with six stereoisomers, using a common simple synthetic strategy. For these efficient syntheses, highly regio- and steroselective processes for the preparation of bromohydrin and *anti*-epoxide intermediates were applied. In order to access the unique *O*-linked carbadisaccharide structure, coupling of chlorohydrin as a donor and *anti*-epoxide as an acceptor was achieved using catalytic BF_3_·Et_2_O. Most of the synthesized compounds exhibited selectively significant inhibitory activity against α-glycosidase derived from yeast. The strongest analog showed almost 50 times the activity of the positive control, deoxynojirimycin.

## 1. Introduction

As the first WHO Global report says “422 million adults were living with diabetes in 2014” and “diabetes caused 1.5 million death in 2012”, conquering diabetes or obesity is one of the most serious problems facing the humankind [1]. Discovering new potent α-glucosidase inhibitors is one way that pharmacists can contribute to resolving this problem. Successful and well-known examples include the clinically used anti-diabetes drugs acarbose, voglibose, and miglitol. Acarbose and voglibose molecules contain carbasugar moieties, whereas miglitol has an azasugar structure. Most candidates for glycosidase inhibitors are nitrogen-containing molecules, such as azasugars, bicyclic molecules with a nitrogen atom at the juncture, and *N*-linked pseudo-oligosaccharides [2]. Recent studies on thiosugar-containing α-glucosidase inhibitors have also made progress [3]. However, there have been only a few reports on pseudo-oligosaccharides constructed of only carbasugars [4,5,6,7,8]. Shing and Hudlicky independently synthesized such molecules, providing a new class of unique glycosidase inhibitors [4,5,6,7]. As this background shows, the synthetic study of carba-oligosaccharides as potential glycosidase inhibitors is a challenging step into a new research area for diabetes drugs discovery.

In the course of our continuing studies on the synthesis of bioactive marine natural products, we have studied and reported on the total syntheses of pericosines [9,10,11,12,13,14,15]. Pericosines A–E (**1**–**6**) are unique carbasugar-type metabolites of the fungus *Periconia byssoides* OUPS-N133, which was originally isolated from the sea hare *Aplysia kurodai* [16,17,18]. As pericosine A (**1**) showed remarkable anticancer activity, several other research groups have also undertaken and reported the syntheses of pericosines [19,20,21,22,23,24,25]. As such, our synthetic strategy towards pericosines has changed to a biomimetic method, in which two kinds of epoxides act as common intermediates [12,13,14,15]. Recently, the Cichewicz group reported the isolation of maximiscin, which shows potent inhibition against melanoma cell line UACC-62 and contains a pericosine moiety, as a fungal metabolite of *Tolypocladium* sp. [26,27]. However, our attention was focused on the co-isolation of (+)-pericosine A (**1**) and precursor pericoside (**7**) from the fungal extract (our second synthetic intermediate, *syn*-epoxide **11**, extracted later) [27]. These natural products can reduce the antifungal activities of antibiotics. Another noteworthy synthesis is that of a pericosine analogue, along with cyathiformine B, streptol, and MK7607, using the regio- and stereo-controlled ring opening of allylic oxides reported by Lewis and co-workers [28]. These studies implied that the syntheses of pericosines will become increasingly important in the future.

**Figure 1:**
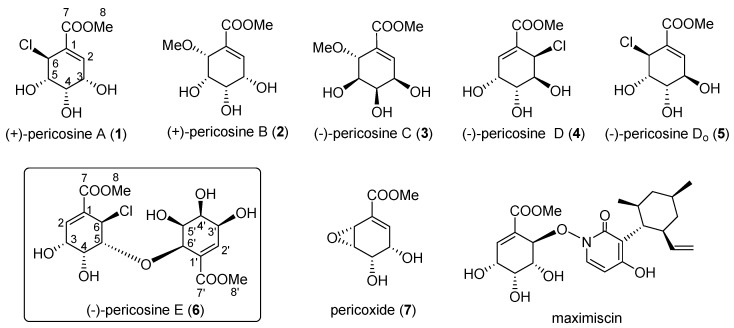
Structures of pericosines (**1**–**6**) and related natural products.

Among the pericosine family, pericosine E (**6**) is extremely unique, containing an *O*-linked carbadisaccharide structure between the pericosine-A-like moiety and pericosine-B-like moiety with the opposite absolute configurations (Figure 1). Indeed, (−)-pericosine E (**6**) has the structure of (−)-pericosine A and that of (+)-pericosine B linked together. To our knowledge, pericosine E (**6**) is the only example of a natural *O*-linked carbadisaccharide to date [29]. Furthermore, natural pericosine E (**6**) was reported to exist as an enantiomeric mixture [17]. As the stereochemistry is highly complicated, the synthesis of **6** and its analogs presents an exciting challenge toward molecules with biological activity, such as glycosidase inhibitory activities. A part of this work, the first total synthesis of (−)-**6**, was recently published in preliminary form [14]. The synthetic strategy and newly developed technologies in the previous paper could be applied to the as yet unsynthesized natural minor enantiomer (+)-pericosine E, along with its analogs. Herein, we describe the enantiospecific syntheses of both enantiomers and six diastereomers of **6**, and their glycosidase inhibitory activities.

## 2. Results and Discussion

### 2.1. Retrosynthetic Strategy

**Scheme 1:**
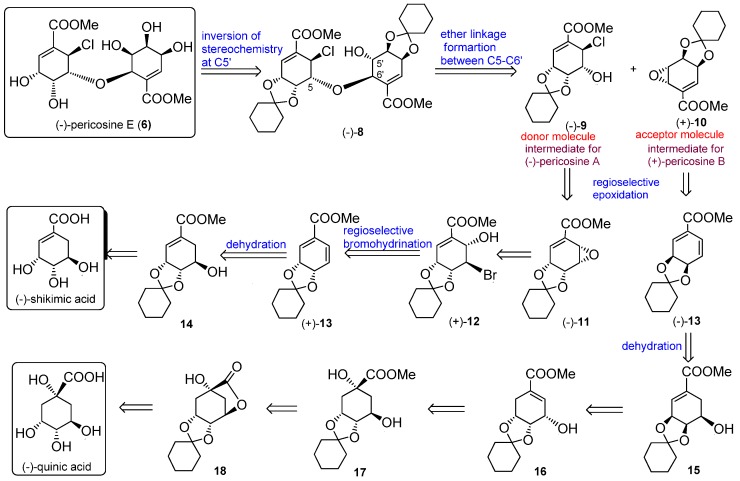
Retrosynthetic strategy of (−)-pericosine E (**6**).

Based on our previous synthetic route for pericosines A–C, we envisioned the retrosynthesis of (−)-**6** as summarized in Scheme 1 [13,14]. In this strategy, we illustrated the absolute configuration of **6** as being made up of (−)-pericosine A (**1**) and (+)-pericosine B (**2**). This structural pattern is denoted as (−pA, +pB)-type hereafter for better understanding of the puzzling stereochemistry in pericosine E and its analogs. The former corresponds with donor chlorohydrin **9**, while the latter corresponds with acceptor *anti*-epoxide **10**, in the key condensation reaction. As mentioned above, donor **9** could be derived from *syn*-epoxide **11**, which corresponds with a pericoxide proven to be the precursor of pericosine A in the culture of *Tolypocladium* sp. [27]. Therefore, our strategy might be biomimetic. Since both enantiomers of common intermediates of unstable diene (**13**) are available from commercially available (−)-quinic acid or (−)-shikimic acid [14,15,30], the synthesis of (+)-**6**, (+pA, −pB)-type, was also possible using essentially the same approach. Furthermore, coupling chlorohydrin (−)-**9** with *anti*-epoxide (−)-**10** will lead a new analog, (−pA, −pB)-type, which might correspond to undiscovered natural products.

### 2.2. Preparation of Both Enantiomers of Cholohydrin and Anti-Epoxide

In order to achieve these total syntheses effectively, two innovations were required: the regio- and steroselective bromohydrination of unstable diene **13** to bromohydrin **12**, and the epoxidation of **13** to give **10**. The former reaction was carried out with *N*-bromosuccinimide (NBS) in an acetonitrile–H_2_O (3:2) solvent system using a 5 mg/mL substrate concentration, while the latter was realized by the addition of **13** to methyl (1,1,1-trifluoromethyl)dioxirane, TFDO) [31,32], prepared in situ at −15 °C in H_2_O–1,1,1-trifluoroacetone (1:1), affording **10** exclusively. When TFDO was generated and reacted with **13** at 0 °C, the product ratio of **10** and its regioisomer, which was inseparable from **10**, was ca. 15:1. Gradual and careful addition of Oxone^®^ at −15 °C to a H_2_O–trifluoroacetone solvent system for in situ generation of TFDO was required in this process, otherwise the inseparable regioisomer was present in the product [12,28,33]. Details of reaction condition optimization can be found in our previous communication [15].

**Scheme 2:**
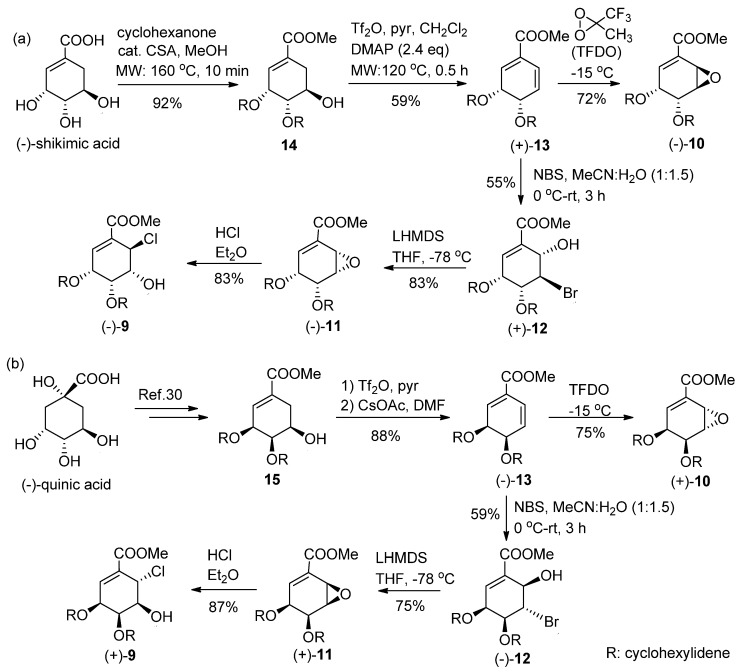
Preparation of both enantiomers of chlorohydrin (**9**) and *anti*-epoxide (**10**). (**a**) Synthesis of (−)-**9** and (−)-**10** from (−)-shikimic acid; (**b**) synthesis of (+)-**9** and (+)-**10** from (−)-quinic acid. LHMDS: lithium hexamethyldisilazide; MW: microwave; NBS: *N*-bromosuccinimide.

Using these new methods, both enantiomers of chlorohydrin **9** and *anti*-epoxide **10** were prepared, as shown in Scheme 2. Scheme 2a summarized syntheses of (−)-**9** and (+)-**10** from (−)-shikimic acid via diene (+)-**13**. A methanol solution of (−)-shikimic acid and cyclohexanone, with a catalytic amount of camphorsulfonic acid (CSA), was heated under microwave irradiation (MW; 160 °C, 30 min) to give alcohol **14** in 92% yield. Then, a CH_2_Cl_2_ solution of obtained alcohol **14**, triflic anhydride (1.2 equivalent (eq.)), pyridine, and dimethylaminopyridine (2.4 eq.) was again heated under MW irradiation (120 °C, 30 min) to give (+)-**13** in 59% yield [34]. After one-pot dehydration, bromohydrination of (+)-**13** was carried out as described above, to afford (+)-**12** in 55% yield, which was then treated with lithium hexamethyldisilazide (LHMDS) at −78 °C, causing an intramolecular S_N_2 reaction to afford epoxide (−)-**11** in an 83% yield. Treatment of epoxide (−)-**11** with HCl in dry diethyl ether yielded chlorohydrin (−)-**9** at an 83% yield. Meanwhile, epoxidation of (+)-**13** with TFDO gave (−)-**10** at a 72% yield.

The preparation of (+)-**9** and (+)-**10** from (−)-quinic acid is illustrated in Scheme 2b. Initially, (−)-quinic acid was converted to alcohol **15** using a known method. MW-aided dehydration of **15**, similar to that described above, afforded a lower yield of the desired (−)-**13**, requiring purification to remove undesired byproducts. A plausible explanation for depressed yield of (−)-**13** in the MW-aided reaction is that instability of the triflate derived from **15** in the reaction temperature might cause some degradation into undesired byproducts. The 4β-oxygen atom in the triflate was deduced to be more positively charged by a neighboring effect of the 5β-OTf group than that in **15**. Then, transformation of **15** into diene (−)-**13** was carried out using a known two-step sequence, 5β-*O*-triflylation of **15** followed by elimination of the leaving group with cesium acetate in *N*,*N*-dimethylformamide (DMF), to afford an 88% yield [35]. Diene (−)-**13** was converted to (−)-**12** and (+)-**10** using the same methods described above. Transformation of (−)-**12** into (+)-**9** via (+)-**11** was carried out using a literature method [14].

### 2.3. Synthesis of Both Enantiomers of Pericosine E and Analogs

**Scheme 3:**
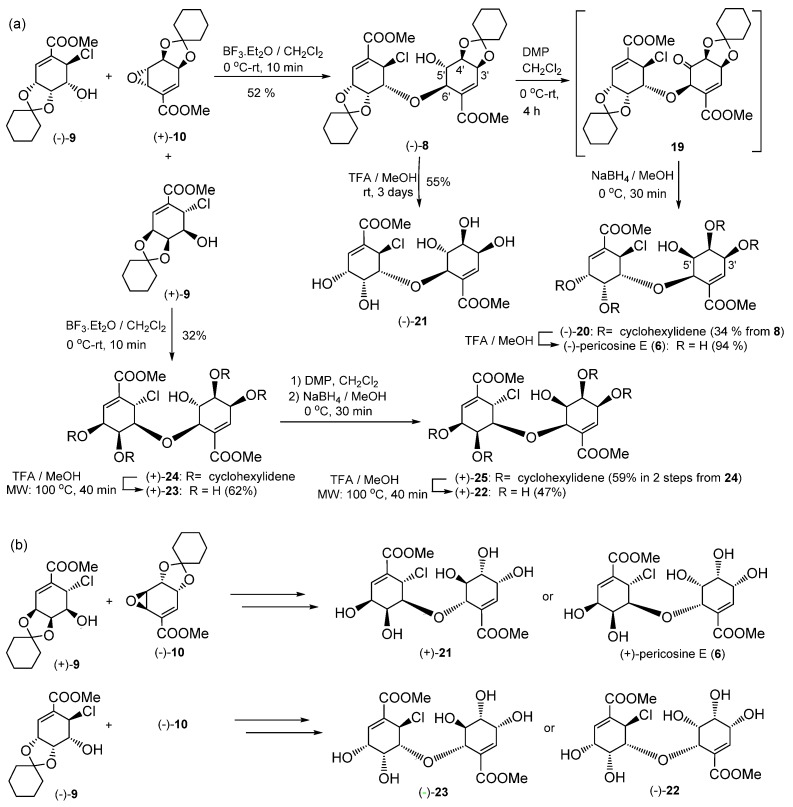
Synthesis of pericosine E (**6**) and analogs. (**a**) Synthesis (−)-**6**, (−)-**21**, (+)-**23**, and (+)-**22** from (+)-**10** and (−)-**9** or (+)-**9**; (**b**) synthesis of (+)-**6**, (+)-**21**, (−)-**23**, and (−)-**22** from (−)-**10** and (+)-**9** or (−)-**9**. DMP: Dess-Martin periodinane; TFA: trifluoroacetic acid.

With the four required intermediates, (+)/(−)-**9** and (+)/(−)-**10**, in hand, the synthesis of (−)-pericosine E (**6**) was carried out from (−)-**9** and (+)-**10**, as shown in Scheme 3. The ether linkage between (−)-**9** and (+)-**10** was formed by treatment with BF_3_·Et_2_O (0.1 eq.) in CH_2_Cl_2_ at room temperature, affording condensation product (−)-**8** at 52% isolated yield [8]. Alcohol (−)-**8** was treated with Dess–Martin periodinane (DMP) to give a crude inseparable mixture of desired ketone **19** and an unidentified compound. Without separation, the crude mixture containing **19** was reduced with NaBH_4_ to afford epimerized alcohol (−)-**20** in 34% yield over two steps. The stereochemistry of (−)-**8** and (−)-**20** was confirmed based on their detailed two-dimensional (2D) NMR analyses. In the NOESY spectrum of (−)-**8**, H-6′ correlated with H-4′ and 5′-OH, supporting the configurations of H-5′ and H-6′. For (−)-**20**, the NOESY cross peak H-3′/H-5′ suggested the configuration of H-5′. Finally, treatment of (−)-**20** with trifluoroacetic acid in methanol gave the desired product, (−)-**6**, whose spectral data, except specific rotation, showed satisfactory agreement with the natural product. The specific rotation of synthetic **6** was [α]_D_ −68.3, whereas that reported for natural **6** was only −31.5 [17]. Thus, we achieved the first total synthesis of (−)-pericosine E (**6**) and assigned the absolute configuration of the naturally dominant enantiomer of **6** to (3*R*,4*R*,5*R*,6*R*)-methyl 6-chloro-3,4-dihydroxy-5-{[(1*R*,4*S*,5*S*,6*S*)-4,5,6-trihydroxy-2-(methoxycarbonyl)cyclohex-2-en-1-yl]oxy}cyclohex-1-enecarboxylate. The results suggested a (−)-**6**/(+)-**6** ratio of roughly 3:1 in the natural product.

The similar deprotection of (−)-**8** progressed slowly, taking three days, and afforded the 5-epimer of (−)-pericosine E, (−)-**21**, at a 55% yield. The same deprotection aided by MW irradiation (100 °C, 5 min) resulted in a lower yield (34%) of (−)-**21**.

We continued the synthesis of the naturally occurring minor enantiomer (+)-**6** and its analogs using the same strategy. (+)-Pericosine E (**6**) and its C5-epimer, (+)-**21**, were synthesized from (+)-**9** and (−)-**10** (Scheme 3b).

Analog (+)-**22**, which corresponds to the (+pA, −pB)-type, and its epimer (+)-**23** were also synthesized from (+)-**9** and (+)-**10** (Scheme 3a). Ether formation between (+)-**9** and (+)-**10** to give (+)-**24**, followed by Dess–Martin oxidation and NaBH_4_ reduction, proceeded to afford (+)-**25** in a similar fashion to the synthesis of **6**. However, the final deprotection of (+)-**24** or (+)-**25** at room temperature did not occur, even after a prolonged reaction time. Thus, MW-aided deprotection was applied to this step (100 °C, 40 min) to afford the desired carba-disaccharides (+)-**23** and (+)-**22** in 47% and 62% yields, respectively. The configurations of **24** and **25** were confirmed by NOESY analyses. Cross peaks H-6′/H-4′ and 5′-OH were observed in the NOESY spectrum of **24**, as seen for **8**. The NOESY spectrum of **25** showed H-3′/H-5′ correlation, as seen for **20**. The enantiomer (−)-**22**, (−pA, −pB)-type, and its epimer (−)-**23** were prepared from (−)-**9** and (−)-**10** in the same manner (Scheme 3b).

In order to extend this scheme to a variety of stereoisomeric analogs, such as (+pA, +pC), (+pA, −pC), (−pA, +pC), and (−pA, −pC)-type of compounds, we attempted the challenging coupling of *syn*,*syn*-epoxide (−)-**11** with alcohols **15** and (+)/(−)-**9** under various conditions. Unfortunately, none of the trials gave the desired *O*-linked carba-disaccharides derived from **11**, despite the epoxide being consumed. These failures were contrary to our expectations for **11** as an acceptor molecule, as it seemed that **11** would react with alcohols more easily than **10** due to the steric demand of **11**. The reason for condensations not occurring with **11** is still not known.

### 2.4. Evaluation of Inhibitory Activities against Glycosidases

Eight samples synthesized here were used in a biological assay against three glycosidases, namely α-glucosidase (yeast), β-glucosidase (sweet almond), and α-mannosidase (Jack bean) [36]. The results are shown in Table 1. Six carba-disaccharides of eight samples showed significant α-glucosidase inhibition, but no inhibition was observed against β-glucosidase or α-mannosidase. Naturally preferred enantiomer (−)-**6** and its epimer (−)-**21** showed α-glucosidase inhibitory activity, with almost one-third the potency of deoxynojirimycin (DNJ), used as a positive control. The IC_50_ values were 1.5 × 10^−3^ M for (−)-**6** and 1.8 × 10^−3^ M for (−)-**21**. To our surprise, the naturally occurring minor enantiomer, (+)-**6**, gave better inhibition (IC_50_: 3.1 × 10^−5^ M) than the major enantiomer, (−)-**6**. Among all the compounds synthesized in this study, (−)-**22**, (−pA, −pB)-type exhibited the strongest activity, being almost 50-fold more potent than DNJ, whereas enantiomer (−)-**23** showed no activity. From these results, compounds made from (−)-**10** as an acceptor are generally excellent inhibitors.

## 3. Conclusions

We have achieved the first total synthesis of both enantiomers of pericosine E (**6**), which are metabolites of *Periconia byssoides* OUPS-N133 isolated from the sea hare *Aplysia kurodai*. The total synthesis elucidated the absolute configuration of the naturally preferred enantiomer to be (−)-**6**. Using this simple and efficient strategy, pericosine E and seven further stereoisomers were prepared. Almost all the synthesized chlorine-containing *O*-linked carbadisaccharides showed significant inhibitory activity against only α-glycosidase derived from yeast. In general, compounds containing a (−)-pericosine B-like portion as an acceptor showed better activity. The most potent compound, (−)-**22**, exhibited ca. 50 times the α-glycosidase inhibitory activity of DNJ, a positive control. These results suggest that *O*-linked carba-oligosaccharides based on pericosine E are promising seeds for a new class of diabetes drugs. Further study of the syntheses of *O*-linked carbadisaccharides without chlorine is ongoing. The anti-glycosidase assay will elucidate the role of the chlorine atom.

## 4. Experimental Section

Infrared (IR) spectra were obtained using a PerkinElmer FT-IR spectrometer 1720X (Perkin Elmer, Waltham, MA, USA). High Resolution Mass Spectra (HRMS) were obtained using a JEOL JMS-700 (2) mass spectrometer (JEOL, Tokyo, Japan). NMR spectra were recorded at 27 °C on Agilent 300-, 400-MR-DD2 (Agilent Technologies, Santa Clara, CA, USA), Agilent 600-DD2 (Agilent Technologies, Santa Clara, CA, USA), Varian Marcury-300BB (Varian, Palo Alto, CA, USA), and Varian Unity-500 spectrometers in CDCl_3_ using tetramethylsilane (TMS) as the internal standard. Liquid column chromatography was conducted on silica gel (Nacalai, silica gel 60, mesh 70–230 or 230–400). Analytical thin layer chromatography (TLC) was performed on pre-coated Merck glass plates (silica gel 60 F_254_) and compounds were detected by dipping the plates in an ethanol solution of phosphomolybdic acid, followed by heating. Microwave-aided reactions were carried out using a Biotage Initiator^®^ reactor (PartnerTech Atvidaberg AB for Biotage Sweden AB, Uppsala, Sweden). Flash chromatography was carried out using Biotage Isolera One^®^ purification system (PartnerTech Atvidaberg AB for Biotage Sweden AB, Uppsala, Sweden). Dry CH_2_Cl_2_, dry tetrahydrofuran (THF), NaBH_4_, trifluoroacetic acid (TFA), pyridine, and NBS were purchased from Wako Pure Chemical Industries (Wako Pure Chemical Industries, Tokyo, Japan). *meta*-Chloroperbenzoic Acid (*m*CPBA) and hexamethyldisilazane (HMDS) were purchased from nacalai tesque, Inc. (nacalai tesque, Inc., Kyoto, Japan). Cyclohexanone, camphorsulfonic acid (CSA), HCl in Et_2_O, trifluoroacetone, BF_3_·Et_2_O, and *n*-BuLi were purchased from Sigma-Aldrich Co. LLC. (St. Louis, MO, USA). Tf_2_O, CsOAc, and Dess-Martin periodinane (DMP) were purchased from TCI (Tokyo Chemical Industry Co. Ltd., Tokyo, Japan). (−)-Shikimic acid was purchased from Carbosynth, Ltd. (UK). (−)-Quinic acid was purchased from Merck (Merck & Co., Inc., Darmstadt, Germany). α-Glucosidase (Yeast, lot. 26010), β-glucosidase (Sweet Almond, lot. 81241), α-mannosidase (Jack Bean, lot. 055K7047), and deoxymannojirimycin were purchased from Sigma-Aldrich Co. LLC. (St. Louis, MO, USA). 1-Deoxynojirimycin was isolated from leaves of the plant *Morus alba* L.

### 4.1. Bromohydrination of (+)*-**13***

NBS (48 mg, 1.2 mmol) at 0 °C was added with stirring to a solution of diene (+)-**13** (58 mg, 0.23 mmol) in acetonitrile-H_2_O (1:1.5, 12 mL). After stirring for 3 h at room temperature (RT), the reaction mixture was treated with aqueous (aq.) Na_2_S_2_O_3_ (10 mL) and saturated (sat.) aq. NaHCO_3_ (10 mL) and extracted with EtOAc (3 × 30 mL). The combined organic layers were dried over MgSO_4_ and filtered, and the solvent was removed under reduced pressure to afford a crude residue. Purification by silica gel column chromatography (CH_2_Cl_2_) afforded (+)-**12** (44 mg, 55%).

(+)-**12**: Colorless oil; [α]${}_{D}^{20}$ +23.1 (*c* 0.06, CHCl_3_); IR (liquid film) ν_max_ 3524 (OH), 1715 (C=O), 1660 (C=C) cm^−1^; ^1^H-NMR (CDCl_3_, 400 MHz, ppm) δ 1.25–1.70 (10H, m), 3.46 (1H, d, *J* = 10.7 Hz, 6-OH), 3.84 (3H, s, COOMe), 4.59 (1H, dd, *J* = 4.0, 2.8 Hz, H-5), 4.67 (1H, dddd, *J* = 4.9, 4.1, 1.9, 1.1 Hz, H-4), 4.74 (1H, br dd, *J* = 10.7, 2.7 Hz, H-6), 4.84 (1H, dd, *J* = 4.5, 3.3 Hz, H-3), 6.88 (1H, dd, *J* = 3.3, 1.0 Hz, H-2); ^13^C-NMR (CDCl_3_, 100 MHz, ppm) δ 23.6, 23.8, 24.7, 35.8, 37.7, 45.1, 52.4, 66.8, 71.3, 75.4, 112.8, 129.8, 135.6, 165.9; HRMS *m*/*z* calcd. for C_14_H_19_O_5_^79^Br (M)^+^, 346.0416; found, 346.0415, *m*/*z* calcd. for C_14_H_19_O_5_^81^Br (M)^+^, 348.0396; found, 348.0391.

### 4.2. Methyl (3R,4R,5S,6S)-3,4-O-cyclohexilidene-3,4-dihydroxy-5,6-epoxy-1-cyclohex-ene-1-carboxy-late (−)*-**11***

To a solution of 1,1,1,3,3,3-hexamethyldisilazane (0.19 mL, 0.81 mmol) in dry THF (3 mL), 1.6 M *n*-BuLi in hexane (0.56 mL, 0.81 mmol) was added at −78 °C to give LHMDS. After 30 min the prepared LHMDS solution was added dropwise to a solution of (+)-**12** (0.28 g, 0.81 mmol) in THF (5 mL) at −78 °C through a steel cannula under argon atmosphere. After stirring the reaction mixture for 1 h at −78 °C, followed by warming to RT over 1 h, the reaction mixture was treated with sat. aq. NH_4_Cl (20 mL) and extracted with EtOAc (3 × 30 mL). The combined organic layers were dried over MgSO_4_ and filtered, and the solvent was removed under reduced pressure to afford a crude residue. Purification by silica gel column chromatography (Hexane–EtOAc, 3:1) afforded (−)-**11** (0.18 g, 83%) as crystals. (−)-**11**: White crystals (CH_2_Cl_2_); mp 81–84 °C; [α]${}_{D}^{21}$ −5.4 (*c* 0.98, CHCl_3_); IR (KBr) ν_max_ 1731 (C=O), 1650 (C=C) cm^−1^; ^1^H-NMR (CDCl_3_, 400 MHz, ppm) δ 1.35–1.80 (10H, m), 3.74 (1H, ddd, *J* = 4.1, 2.8, 2.0 Hz, H-5), 3.83 (3H, s, COOMe), 4.03 (1H, dd, *J* = 4.1, 2.1 Hz, H-6), 4.48 (1H, dd, *J* = 6.6, 2.8 Hz, H-4), 4.73 (1H, ddd, *J* = 6.6, 5.5, 2.0 Hz, H-3), 7.14 (1H, dd, *J* = 5.5, 2.0 Hz, H-2); ^13^C-NMR (CDCl_3_, 100 MHz, ppm) δ 23.9, 24.1, 25.1, 34.5, 36.8, 49.0, 52.4, 56.2, 69.7, 72.1, 109.4, 132.5, 137.5, 165.4; HRMS *m*/*z* calcd. for C_14_H_18_O_5_ (M)^+^, 266.1155; found. 266.1150.

### 4.3. Methyl (3R,4R,5R,6R)-6-chloro-3,4-O-cyclohexylidene-3,4,5-trihydroxy-1-cyclo hexene-1-carbo-xylate (−)*-**9***

To a solution of (−)-**11** (55.9 mg, 0.21 mmol) in dry Et_2_O (1 mL), 1 M HCl in Et_2_O (0.31 mL, 0.31 mmol) was added at 0 °C. After stirring for 1 h, the solvent was removed under vacuum to afford a crude residue that was purified by preparative TLC (Hexane–EtOAc, 4:1) to afford (−)-**9** (57.5 mg, 90%) as colorless crystals. (−)-**9**: Colorless crystals (CHCl_3_) mp 125–128 °C; [α]${}_{D}^{25}$ −165.0 (*c* 0.3, CHCl_3_); IR (KBr) ν_max_ 3360 (OH), 1725 (C=O), 1649 (C=C) cm^−1^; ^1^H-NMR (CDCl_3_, 400 MHz, ppm) δ 1.20–1.80 (10H, m), 2.66 (1H, d, *J* = 2.4 Hz, 5-OH), 3.83 (3H, s, COOMe), 4.30 (1H, ddd, *J* = 3.9, 3.8, 2.3 Hz, H-5), 4.70 (1H, ddd, *J* = 7.5, 3.9, 0.4 Hz, H-4), 4.77 (1H, dd, *J* = 7.5, 3.0 Hz, H-3), 5.04 (1H, d, *J* = 3.9 Hz, H-6), 7.18 (1H, d, *J* = 3.2 Hz, H-2); ^13^C-NMR (CDCl_3_, 100 MHz, ppm) δ 23.5, 23.9, 25.0, 33.4, 36.1, 50.9, 52.4, 67.2, 69.6, 71.4, 110.8, 130.2, 137.8, 164.9; HRMS *m*/*z* calcd. for C_14_H_19_O_5_^35^Cl (M)^+^ 302.0921, found 302.0925, *m*/*z* calcd. for C_14_H_19_O_5_^37^Cl (M)^+^, 304.0891; found, 304.0903.

### 4.4. Synthesis of Anti-Epoxide (−)*-**10***

To a solution of (+)-**13** (60.0 mg, 0.24 mmol) and NaHCO_3_ (0.20 g, 2.4 mmol) in trifluoroacetone-H_2_O (1:1, 2 mL) at −15 °C, Oxone^®^ was added every 15 min (four portions, each portion 0.073 g, 0.12 mmol) with stirring. After 3 h, *tert*-butyl methyl ether (TBME) (10 mL) was added and the reaction mixture was filtered through Celite. The filtrate was treated with sat. aq. NaHCO_3_ (10 mL) and extracted with TBME (3 × 10 mL). The combined organic layers were dried over MgSO_4_, filtered, and the solvent was removed under reduced pressure to afford a crude residue, which was purified by column chromatography (Hexane–EtOAc, 5:1) to afford (−)-**10** (46.0 mg, 72%) as a colorless oil. (−)-**10**: Colorless oil; [α]${}_{D}^{23}$ −20.4 (*c* 0.29, CHCl_3_); IR (liquid film) ν_max_ 1730 (C=O), 1647 (C=C) cm^−1^; ^1^H-NMR (CDCl_3_, 500 MHz, ppm) δ 1.35–1.70 (10H, m), 3.69 (1H, br dd, *J* = 3.7, 2.1 Hz, H-5), 3.84 (3H, s, COOMe), 3.99 (1H, ddd, *J* = 3.7, 1.6, 0.7 Hz, H-6), 4.58 (1H, dd, *J* = 6.9, 2.3 Hz, H-3), 4.80 (1H, br d, *J* = 6.9 Hz, H-4), 6.83 (1H, m, H-2); ^13^C-NMR (CDCl_3_, 125 MHz, ppm) δ 23.7, 23.9, 24.9, 35.3, 37.5, 46.1, 49.3, 52.3, 70.0, 70.8, 111.7, 127.2, 140.3, 165.5; HRMS *m*/*z* calcd. for C_14_H_18_O_5_ (M)^+^, 266.1156; found, 266.1158.

### 4.5. Bromohydrination of (−)*-**12***

Diene (−)-**13** (1.5 g, 5.9 mmol) was converted to (−)-**12** (1.2 g, 57% yield) using the same procedure as for (+)-**12**.

### 4.6. Synthesis of (+)-***11***

Bromohydrin (−)-**12** (556 mg, 4.3 mmol) was converted to (+)-**11** (319 mg, 75% yield) using the same procedure described above.

### 4.7. Synthesis of (+)-***9***

Epoxide (+)-**11** (386 mg, 1.4 mmol) was converted to chlorohydrin (+)-**9** (395 mg, 90% yield) using the same procedure described above.

### 4.8. Synthesis of Anti-Epoxide (+)-***10***

Diene (−)-**13** (1.5 g, 5.9 mmol) was oxidized to (+)-**10** (1.2 g, 75% yield) using a procedure similar to that for (−)-**10**. (+)-**10**: Colorless oil; [α]${}_{D}^{25}$ +24.7 (*c* 0.68, CHCl_3_); IR (liquid film) ν_max_ 1722 (C=O), 1654 (C=C) cm^−1^; ^1^H-NMR (CDCl_3_, 600 MHz, ppm) δ 1.34–1.70 (10H, m), 3.68 (1H, dd, *J* = 3.6, 2.4 Hz, H-5), 3.84 (3H, s, COOMe), 3.99 (1H, ddd, *J* = 3.8, 1.7, 0.6 Hz, H-6), 4.57 (1H, dd, *J* = 6.8, 2.4 Hz, H-3), 4.80 (1H, br d, *J* = 6.8 Hz, H-4), 6.83 (1H, m, H-2); ^13^C-NMR (CDCl_3_, 150 MHz, ppm) δ 23.7, 23.9, 24.8, 35.2, 37.4, 46.1, 49.3, 52.3, 70.0, 70.8, 111.6, 127.1, 140.3, 165.5; HRMS *m*/*z* calcd. for C_14_H_18_O_5_ (M)^+^, 266.1156; found, 266.1161.

### 4.9. Synthesis of (−)-***8*** from (−)-***9*** and (+)-***10***

To a solution of chlorohydrin (–)-**9** (73.0 mg, 0.24 mmol) and epoxide (+)-**10** (53.5 mg, 0.20 mmol) in CH_2_Cl_2_ (0.6 mL), BF_3_·Et_2_O (5 μL, 0.019 mmol) was added at 0 °C. After stirring for 10 min at RT, the reaction mixture was treated with Et_3_N (5 μL, 0.035 mmol) and concentrated under vacuum to afford a crude residue, which was purified by silica gel column chromatography (Hexane–EtOAc, 3:1) to afford (–)-**8** (58.9 mg, 52%) as a white amorphous solid. (−)-**8**: White amorphous solid; [α]${}_{D}^{25}$ −68.3 (*c* 0.21, CHCl_3_); IR (liquid film) ν_max_ 3431 (OH), 1729 (C=O), 1657 (C=C) cm^−1^; ^1^H-NMR (acetone-*d*_6_, 600 MHz, ppm) δ 1.30–1.80 (20H, m), 3.68 (3H, s, COOMe-8′), 3.81 (3H, s, COOMe-8), 4.01 (1H, ddd, *J* = 7.3, 6.1, 3.6 Hz, H-5′), 4.21 (1H, dd, *J* = 7.3, 6.2 Hz, H-4′), 4.37 (1H, ddd, *J* = 6.2, 1.4, 1,2 Hz, H-6′), 4.49 (1H, dd, *J* = 4.7, 3.9 Hz, H-5), 4.69 (1H, ddd, *J* = 6.2, 3.8, 1.2 Hz, H-3′), 4.74 (1H, d, *J* = 3.8 Hz, 5′-OH), 4.84 (1H, dd, *J* = 7.0, 3.9 Hz, H-4), 4.88 (1H, ddd, *J* = 7.0, 2.9, 0.6 Hz, H-3), 5.15 (1H, d, *J* = 4.4 Hz, H-6), 6.54 (1H, dd, *J* = 3.8, 1.4 Hz, H-2′), 6.95 (1H, d, *J* = 2.9 Hz, H-2); ^13^C-NMR (acetone-*d*_6_, 150 MHz, ppm) δ 24.4, 24.4, 24.7, 24.7, 25.7, 25.8, 34.9, 36.3, 36.6, 38.8, 51.6, 52.2, 52.4, 71.0, 71.6, 72.6, 73.7, 76.3, 78.9, 79.5, 111.4, 111.6, 131.5, 133.2, 134.8, 138.8, 165.8, 167.2; HRMS *m*/*z* calcd. for C_28_H_37_O_10_^35^Cl (M)^+^, 568.2075; found, 568.2073.

### 4.10. Dess-Martin Oxidation of (−)-***8*** Followed by NaBH_4_ Reduction

To a solution of alcohol (−)-**8** (0.22 g, 0.38 mmol) in CH_2_Cl_2_ (8 mL), DMP (0.20 g, 0.46 mmol) was added at 0 °C, with stirring. The reaction mixture was warmed to RT and stirred for 4 h. The reaction mixture was treated with aqueous Na_2_S_2_O_3_ (20 mL) and sat. aq. NaHCO_3_ (10 mL), then extracted with TBME (3 × 20 mL). The combined organic layers were washed with brine (30 mL) and water (30 mL), dried over MgSO_4_, filtered, and concentrated under vacuum to afford an inseparable mixture (0.20 g) of enone **19** and undefined compound **19u**, whose carbon skeleton is same as **19** suggested by 2D-NMR analysis of the mixture. Crude **19** and **19u**: Oil; HRMS *m*/*z* calcd. for C_28_H_35_O_10_^35^Cl (M)^+^ of **19**, 566.1919; found, 566.1923; ^1^H-NMR (CDCl_3_, 600 MHz, ppm) δ **19**: 1.20–2.00 (20H, m), 3.77 (3H, s, COOMe), 3.85 (3H, s, COOMe), 4.30 (1H, dd, *J* = 6.2, 3.2 Hz, H-5), 4.58 (1H, dd, *J* = 6.5, 0.9 Hz, H-4′), 4.70 (1H, ddd, *J* = 5.9, 3.0, 1.2 Hz, H-3), 4.77 (1H, dd, *J* = 5.9, 3.2 Hz, H-4), 4.96 (1H, ddd, *J* = 6.5, 4.1, 0.9 Hz, H-3′), 5.05 (1H, br d, *J* = 6.2 Hz, H-6), 5.12 (1H, m, H-6′), 6.75 (1H, br d, *J* = 3.0 Hz, H-2), 6.83 (1H, dd, *J* = 4.1, 1.8 Hz, H-2′); **19a**: 1.20–2.00 (20H, m), 3.79 (3H, s, COOMe), 3.83 (3H, s, COOMe), 4.15 (1H, ddd, *J* = 5.9, 1.5, 0.9 Hz, H-4′), 4.21 (1H, br s, H-6′), 4.45 (1H, t, *J* = 4.4 Hz, H-5), 4.69 (1H, ddd, *J* = 5.9, 3.5, 0.6 Hz, H-3′), 4.78 (1H, dd, *J* = 7.6, 2.7 Hz, H-3), 4.90 (1H, dd, *J* = 7.6, 4.4 Hz, H-4), 5.14 (1H, d, *J* = 4.4 Hz, H-6), 6.78 (1H, dd, *J* = 3.5, 0.9 Hz, H-2′), 7.03 (1H, d, *J* = 2.7 Hz, H-2); ^13^C-NMR (CDCl_3_, 150 MHz, ppm) δ 23.52 (CH_2_), 23.54 (CH_2_), 23.66 (CH_2_), 23.75 (CH_2_), 23.78 (CH_2_), 23.83 (CH_2_), 23.88 (CH_2_), 23.92 (CH_2_), 24.86 (CH_2_), 24.91 (CH_2_), 25.0 (CH_2_), 25.1 (CH_2_), 29.7 (CH_2_), 34.7 (CH_2_), 35.4 (CH_2_), 35.6 (CH_2_), 36.0 (CH_2_), 36.5 (CH_2_), 37.0 (CH_2_), 37.3 (CH_2_), 49.2 (CH, **19u**), 52.22 (CH_3_), 52.26 (CH_3_), 52.34 (CH_3_), 52.4 (CH_3_), 53.1 (CH, **19**), 70.4 (CH, **19u**), 71.3 (CH, **19**), 71.8 (CH, **19**), 72.6 (CH, **19u**), 73.2 (CH, **19**), 74.8 (CH, **19**), 76.2 (CH, **19**), 77.2 (CH, **19**), 77.7 (CH, **19a**), 78.8 (CH, **19**), 79.1 (CH, **19u**), 79.8 (CH, **19u**), 93.7 (Cq, **19**), 111.3 (Cq), 111.4 (Cq), 112.3 (Cq), 113.7 (Cq), 129.1 (Cq, **19u**), 129.7 (Cq, **19u**), 130.8 (Cq, **19**), 133.6 (CH, **19**), 134.0 (Cq, **19**), 136.9 (CH, **19**), 137.7 (CH, **19u**), 139.6 (CH, **19u**), 164.9 (Cq, **19u**), 165.2 (Cq, **19**), 165.5 (Cq, **19**), 166.9 (Cq, **19u**), 201.2 (Cq, **19**).

To a solution of NaBH_4_ (13.2 mg, 0.35 mmol) in MeOH (2.5 mL), a solution of crude **19** and **19u** (202 mg) in MeOH (10 mL) was added dropwise at 0 °C. After stirring for 30 min, the reaction mixture was treated with sat. aq. NH_4_Cl (30 mL) and extracted with CH_2_Cl_2_ (3 × 30 mL). The organic layer was washed with brine (30 mL), dried over MgSO_4_, filtered, and concentrated under vacuum to afford a crude residue. Purification by silica gel column chromatography (Hexane–EtOAc, 3:1) afforded (−)-**20** (74.0 mg, 34% from **7**) as a white amorphous solid. (−)-**20**: White amorphous solid; [α]${}_{D}^{25}$ −67.5 (*c* 0.30, CHCl_3_); IR (liquid film) ν_max_ 3421 (OH), 1719 (C=O), 1656 (C=C) cm^−1^; ^1^H-NMR (CDCl_3,_ 600 MHz, ppm) δ 1.20–2.00 (20H, m), 3.71 (1H, m, H-5′), 3.77 (3H, s, COOMe), 3.86 (3H, s, COOMe), 4.34 (1H, dd, *J* = 5.0, 4.7 Hz, H-5), 4.43–4.45 (1H, m, H-4′), 4.55 (1H, br d, *J* = 6.5 Hz, H-6′), 4.60 (1H, ddd, *J* = 5.6, 3.5, 0.6 Hz, H-3′), 4.77 (1H, dd, *J* = 7.6, 2.7 Hz, H-3), 4.88 (1H, dd, *J* = 7.6, 4.7 Hz, H-4), 5.05 (1H, br s, OH), 5.07 (1H, d, *J* = 5.0 Hz, H-6), 6.78 (1H, dd, *J* = 3.2, 0.9 Hz, H-2′), 6.69 (1H, d, *J* = 2.7 Hz, H-2); ^13^C-NMR (CDCl_3_, 150 MHz, ppm) δ 23.50, 23.55, 23.76, 23.83, 24.9, 25.1, 33.1, 35.0, 36.0, 37.5, 50.5, 52.2, 52.4, 68.6, 70.6, 72.1, 72.3, 74.8, 74.9, 78.9, 111.1, 112.0, 129.2, 129.8, 138.6, 139.5, 165.1, 166.7; HRMS *m*/*z* calcd. for C_28_H_37_O_10_^35^Cl (M)^+^, 568.2075; found, 568.2076.

### 4.11. Synthesis of (−)-Pericosine E (***6***)

To a solution of alcohol (−)-**20** (13.3 mg, 0.023 mmol) in MeOH (0.2 mL), TFA (1.8 mL) was added dropwise dropwise at 0 °C. After stirring for 5 h at RT, the reaction mixture was concentrated under vacuum to afford white crystals. The product was purified by preparative TLC (MeOH-CH_2_Cl_2_, 1:9) to afford (−)-**6** (9.0 mg, 94%). (−)-**6**: white crystal; [α]${}_{D}^{24}$ −68.3 (*c* 0.06, EtOH); IR (liquid film) ν_max_ 3431 (OH), 1729 (C=O), 1657 (C=C) cm^−1^; ^1^H-NMR (acetone-*d*_6_, 600 MHz, ppm) δ 3.76 (1H, dd, *J* = 4.1, 2.1 Hz, H-5′), 3.790 (3H, s, COOMe), 3.793 (3H, s, COOMe), 4.06 (1H, br s, H-4′), 4.18 (1H, br d, *J* = 11.1 Hz, OH), 4.20–4.22 (1H, m, H-4′), 4.21 (1H, br s, H-3), 4.23–4.25 (1H, m, H-3′), 4.34–4.36 (1H, m, H-5), 4.53 (1H, d, *J* = 4.1 Hz, H-6′), 5.23 (1H, d, *J* = 2.9 Hz, H-6), 5.33 (1H, br s, OH), 5.61 (1H, br dd, *J* = 8.8, 0.5 Hz, OH), 6.74 (1H, dd, *J* = 2.4, 1.4 Hz, H-2′), 7.01 (1H, d, *J* = 4.4 Hz, H-2); ^13^C-NMR (acetone-*d*_6_, 150 MHz, ppm) δ 52.45 (CH_3_), 52.49 (CH_3_), 53.1 (CH_2_), 65.5 (CH_2_), 66.8 (CH_2_), 69.3 (CH_2_), 70.5 (CH_2_), 72.4 (CH_2_), 77.1 (CH_2_), 85.6 (CH_2_), 129.3 (C), 130.4 (C), 143.2 (CH_2_), 143.5 (CH_2_), 166.1 (Cq), 166.9 (Cq); HRFABMS *m*/*z* calcd. for C_16_H_22_O_10_^35^Cl (M + H)^+^, 409.0901; found, 409.0908.

Spectroscopic data of natural **6** [17]: Oil; [α]_D_ −31.5 (*c* 0.43, EtOH) (racemate as plate from MeOH; mp 213–215 °C; [α]_D_ 0); IR (liquid film) ν_max_ 3326 (OH), 1721 (C=O), 1636 (C=C) cm^−1^; ^1^H-NMR (acetone-*d*_6_, 500 MHz, ppm) δ 3.76 (1H, br s, H-5′), 3.79 (3H, s, COOMe), 3.79 (3H, s, COOMe), 4.07 (1H, br s, H-4′), 4.22 (1H, m, H-2), 4.23 (1H, br s, 4′-OH), 4.25 (1H, br s, H-3), 4.26 (1H, br s, H-3′), 4.36 (1H, m, H-5), 4.53 (1H, d, *J* = 4.1 Hz, H-6′), 5.24 (1H, d, *J* = 3.0 Hz, H-6), 5.37 (1H, br s, 4-OH), 5.64 (1H, br s, 5′-OH), 6.74 (1H, t, *J* = 1.8 Hz, H-2′), 7.01 (1H, d, *J* = 3.9 Hz, H-2); ^13^C-NMR (acetone-*d*_6_, 125 MHz, ppm) δ 52.44 (CH_3_-8), 52.48 (CH_3_-8′), 53.06 (CH_2_-6), 65.57 (CH_2_-3), 66.75 (CH_2_-4), 69.22 (CH_2_-3′), 70.43 (CH_2_-5′), 72.42 (CH_2_-4′), 77.07 (CH_2_-6′), 85.52 (CH_2_-6), 129.23 (Cq-1), 129.91 (Cq-1′), 143.17 (CH_2_-2), 143.50 (CH_2_-2′), 166.09 (CH_3_-8), 166.87 (CH_3_-8′); HRMS *m*/*z* calcd. for C_16_H_22_O_10_^35^Cl (M + H)^+^, 409.0900; found, 409.0904.

### 4.12. Synthesis of (−)-***21***

To a solution of alcohol (−)-**8** (21.6 mg, 0.038 mmol) in MeOH (0.2 mL), TFA (1.8 mL) was added dropwise at 0 °C. After stirring for 3 days at RT, the reaction mixture was concentrated under vacuum to afford white crystals. The product was purified by preparative TLC (MeOH-CH_2_Cl_2_, 1:9) to give (–)-**21** (8.6 mg, 55%). (−)-**21**: white crystal; [α]${}_{D}^{14}$ −47.1 (*c* 0.09, EtOH); IR (liquid film) ν_max_ 3344 (OH), 1723 (C=O), 1657 (C=C) cm^−1^; ^1^H-NMR (acetone-*d*_6_, 600 MHz, ppm) δ 3.72 (3H, s, COOMe), 3.80 (3H, s, COOMe), 4.00 (1H, br dddd, *J* = 5.3, 4.1, 1.2, 0.8 Hz, H-4′), 4.11 (1H, dd, *J* = 5.3, 2.4 Hz, H-4), 4.15 (1H, br t, *J* = 5.0 Hz, H-3), 4.24 (1H, br dd, *J* = 2.7, 2.6 Hz, H-5), 4.25 (1H, br d, 3.2 Hz, H-6′), 64.46 (1H, ddd, *J* = 4.1, 2.7, 0.9 Hz, H-3′), 4.50 (1H, dd, *J* = 5.3, 3.3 Hz, H-5′), 5.23 (1H, d, *J* = 3.0 Hz, H-6), 6.79 (1H, ddd, *J* = 2.7, 1.2, 0.6 Hz, H-2′), 7.01 (1H, d, *J* = 4.7 Hz, H-2); ^13^C-NMR (acetone-*d*_6_, 150 MHz, ppm) δ 52.24 (CH_3_), 52.52 (CH_3_), 52.95 (CH_2_), 65.67 (CH_2_), 65.76 (CH_2_), 66.6 (CH_2_), 69.06 (CH_2_), 70.11 (CH_2_), 76.69 (CH_2_), 83.54 (CH_2_), 129.47 (C), 129.54 (C), 142.5 (CH_2_), 142.7 (CH_2_), 166.0 (Cq), 166.9 (Cq); HRMS *m*/*z* calcd. for C_16_H_21_O_10_^35^Cl (M)^+^, 408.0823; found, 408.0821.

### 4.13. Synthesis of (+)-***24***

To a solution of (+)-**10** (0.22 g, 0.83 mmol) and (+)-**9** (0.26 g, 0.86 mmol) in CH_2_Cl_2_ (3.0 mL), BF_3_·Et_2_O (3 μL, 0.011 mmol) was added at 0 °C, then the reaction mixture was stirred at RT. After 10 min, Et_3_N (20 μL, 0.175 mmol) was added and the mixture was stirred for another 3 h. Solvent removal under reduced pressure gave a crude residue, which was purified by silica gel column chromatography (Hexane–EtOAc, 3:1) to afford (+)-**24** as an amorphous solid (0.15 g, 32%). (+)-**24**: *R*_f_ 0.14 (Hexane–EtOAc, 3:1); [α]${}_{D}^{20}$ +131.1 (*c* 0.135, CHCl_3_); IR (liquid film) ν_max_ 3471 (OH), 1724 (C=O), 1659 (C=C) cm^−1^; ^1^H-NMR (acetone-*d*_6_, 600 MHz, ppm) δ 1.26–1.70 (20H, m), 3.78 (3H, s, COOMe), 3.80 (3H, s, COOMe), 4.03 (1H, dd, *J* = 9.6, 4.7 Hz, H-5′), 4.23 (1H, dd, *J* = 5.9, 5.6 Hz, H-4′), 4.32 (1H, dd, *J* = 4.7, 3.8 Hz, H-5), 4.35 (1H, d, *J* = 4.1 Hz, H-6′), 4.57 (1H, d, *J* = 4.4 Hz, OH), 4.67 (1H, ddd, *J* = 6.1, 3.8, 0.9 Hz, H-3′), 4.69 (1H, dd, *J* = 6.7, 3.8 Hz, H-4), 4.78 (1H, dd, *J* = 6.5, 3.0 Hz, H-3), 5.16 (1H, d, *J* = 4.7 Hz, H-6), 6.63 (1H, d, *J* = 3.9 Hz, H-2′), 6.93 (1H, d, *J* = 2.9 Hz, H-2); ^13^C-NMR (acetone-*d*_6_, 150 MHz, ppm) δ 24.6 (2C), 24.8 (2C), 25.8, 25.9, 35.6, 36.0, 37.0, 38.4, 52.2, 52.4, 52.7, 70.7, 71.1, 71.2, 72.6, 76.2, 77.3, 79.5, 111.2, 111.3, 131.4, 133.2, 135.0, 138.9, 165.9, 167.3; HRMS *m*/*z* calcd. for C_28_H_37_O_10_^35^Cl (M)^+^, 568.2075; found, 568.2074.

### 4.14. Synthesis of (+)-***25***

To a solution of (+)-**24** (170 mg, 0.30 mmol) in CH_2_Cl_2_ (5 mL), DMP (190 mg, 0.39 mmol) was added at 0 °C, and the mixture was stirred for 4 h at RT. The reaction was quenched by the addition of sat. aq. Na_2_S_2_SO_4_ and sat. aq. NaHCO_3_ (10 mL) and extracted with TBME (3 × 20 mL). The combined organic layers were washed with brine (30 mL), dried over MgSO_4_, filtered, and the solvent was removed under reduced pressure to give a crude residue (180 mg). Without purification, the residue was taken up in methanol (5 mL) and the methanol solution (1 mL) of NaBH_4_ (11 mg, 0.29 mmol) was added in four portions at 0 °C. After 30 min, the reaction mixture was quenched by the addition of sat aq. NH_4_Cl (30 mL), extracted with CH_2_Cl_2_ (3 × 30 mL). The organic layer was washed with brine, dried over MgSO_4_, filtered, and the solvent was removed under reduced pressure to give a crude residue, which was purified by silica gel column chromatography (Hexane–EtOAc, 3:1) to afford (+)-**25** (100 mg, 59% in two steps) as an oil. (+)-**25**: *R_f_* 0.14 (Hexane–EtOAc, 3:1); [α]${}_{D}^{20}$ +60.2 (*c* 0.665, CHCl_3_); IR (liquid film) ν_max_ 3460 (OH), 1722 (C=O), 1653 (C=C) cm^−1^; ^1^H-NMR (acetone-*d_6_*, 400 MHz, ppm) δ 1.20–1.70 (20H, m), 3.79 (3H, s, COOMe), 3.81 (3H, s, COOMe), 3.78–3.81 (1H, m, H-5′), 4.37 (1H, dd, *J* = 5.3, 3.9 Hz, H-4′), 4.48 (1H, br dd, *J* = 6.4, 3.5 Hz, H-4), 4.58 (1H, d, *J* = 4.9 Hz, H-6′), 4.75 (1H, d, *J* = 3.7 Hz, H-3′), 4.76 (1H, d, *J* = 3.6 Hz, H-5), 4.81 (1H, ddd, *J* = 6.8, 2.6, 0.8 Hz, H-3), 5.21 (1H, d, *J* = 5.3 Hz, H-6), 6.76 (1H, dd, *J* = 3.9, 0.8 Hz, H-2′), 6.88 (1H, d, *J* = 2.7 Hz, H-2); ^13^C-NMR (acetone-*d_6_*, 100 MHz, ppm) δ 24.5, 24.6, 24.6, 24.8, 25.8, 25.8, 35.1, 35.6, 36.7, 37.7, 52.3, 52.6, 53.3, 69.0, 71.4, 72.7, 72.7, 74.6, 75.1, 79.7, 111.1, 111.8, 131.1, 131.1, 138.2, 140.0, 166.4, 166.8; HRMS *m*/*z* calcd. for C_28_H_37_O_10_^35^Cl (M)^+^, 568.2075; found, 568.2077.

### 4.15. Microwave-Aided Deprotection toward (+)-***22***

To a methanol solution (0.2 mL) of **25** (23.7 mg, 0.042 mmol) in a microwave vial, TFA (1.8 mL) was added at 0 °C. The vial was sealed and irradiated in the MW reactor at 100 °C for 30 min. After cooling, the reaction mixture was condensed under reduced pressure to give a crude residue, which was purified by silica gel column chromatography (MeOH–CH_2_Cl_2_, 1:9) to afford (+)-**22** (8.0 mg, 47%) as an oil. (+)-**22**: *R_f_* 0.3 (MeOH-CH_2_Cl_2_, 1:9); [α]${}_{D}^{20}$ +5.7 (*c* 0.12, EtOH); IR (liquid film) ν_max_ 3404 (OH), 1713 (C=O), 1651 (C=C) cm^−1^; ^1^H-NMR (acetone-*d*_6_, 400 MHz, ppm) δ 3.64-3.82 (2H, m, OH, H-5), 3.78 (3H, s, COOMe), 3.80 (3H, s, COOMe), 3.84-3.98 (2H, m, OH), 3.92 (1H, br d, *J* = 7.3 Hz, OH), 3.99 (1H, s, H-4′), 4.02–4.10 (2H, m, H-4, OH), 4.10–4.24 (2H, m, H-3, OH), 4.27 (1H, br s, H-3′), 4.48 (1H, dd, *J* = 3.1, 2.3 Hz, H-5), 4.57 (1H, d, *J* = 4.3 Hz, H-6′), 5.38 (1H, d, *J* = 3.3 Hz, H-6), 6.80 (1H, br d, *J* = 1.0 Hz, H-2′), 7.04 (1H, d, *J* = 4.7 Hz, H-2); ^13^C-NMR (acetone-*d*_6_, 100 MHz, ppm) δ 51.49 51.52, 52.9, 64.9, 65.3, 68.2, 68.4, 71.2, 73.1, 82.2, 129.0, 129.1, 141.6, 142.5, 165.0, 166.4; HRMS *m*/*z* calcd. for C_16_H_22_O_10_^35^Cl (M + H)^+^, 409.0901; found, 409.0896.

### 4.16. Microwave Aided Deprotection toward (+)-***23***

Using the same procedure as for (+)-**22**, (+)-**24** (22.6 mg, 0.040 mmol) was converted to (+)-**23** (10.0 mg, 62%). (+)-**23**: white crystals; *R_f_* 0.11 (MeOH-CH_2_Cl_2_, 1:9); [α]${}_{D}^{20}$ +75.4 (*c* 0.340, EtOH); IR (liquid film) ν_max_ 3392 (OH), 1714 (C=O), 1652 (C=C) cm^−1^; ^1^H-NMR (acetone-*d*_6_, 300 MHz, ppm) δ 3.75 (1H, br s, H-4′), 3.79 (6H, s, COOMe ×2), 4.02 (1H, br s, H-5′), 4.10 (1H, br s, H-4), 4.20 (1H, br s, H-3), 4.37 (2H, br m, H-3′, H-6′), 4.43 (1H, br s, H-5), 5.24 (1H, br d, *J* = 3.2 Hz, H-6), 6.80 (1H, br s, H-2′), 6.99 (1H, br d, *J* = 2.9 Hz, H-2); ^13^C-NMR (acetone-*d*_6_, 75 MHz, ppm) δ 52.5, 52.5, 54.5, 66.3, 66.4, 66.6, 70.5, 71.9, 77.1, 83.7, 129.7, 130.7, 141.3, 142.8, 166.1, 167.7; HRMS *m*/*z* calcd. for C_16_H_21_O_10_^35^Cl (M)^+^, 408.0823; found, 408.0819.

### 4.17. Synthesis of (+)-***8***

Using the procedure described for (−)-**8**, (+)-**8** (0.092 g, 39%) was prepared from (+)-**9** (0.15 g, 0.5 mmol) and (−)-**10** (0.11 g, 0.4 mmol). (+)-**8**: White amorphous solid; [α]${}_{D}^{20}$ +72.7 (*c* 0.42, CHCl_3_); IR (liquid film) ν_max_ 3431 (OH), 1729 (C=O), 1653 (C=C) cm^−1^; ^1^H-NMR (acetone-*d*_6_, 600 MHz, ppm) δ 1.28–1.80 (20H, m), 3.68 (3H, s, COOMe-8′), 3.81 (3H, s, COOMe-8), 4.03 (1H, ddd, *J* = 7.7, 6.2, 3.5 Hz, H-5′), 4.21 (1H, dd, *J* = 7.7, 6.2 Hz, H-4′), 4.37 (1H, ddd, *J* = 6.2, 1.5, 1,2 Hz, H-6′), 4.49 (1H, dd, *J* = 4.4, 4.1 Hz, H-5), 4.68(1H, ddd, *J* = 6.1, 4.1, 1.2 Hz, H-3′), 4.73(1H, d, *J* = 3.5 Hz, OH), 4.84 (1H, dd, *J* = 7.0, 3.9 Hz, H-4), 4.89 (1H, ddd, *J* = 7.0, 3.0, 0.6 Hz, H-3), 5.15 (1H, d, *J* = 4.7 Hz, H-6), 6.54 (1H, dd, *J* = 4.1, 1.4 Hz, H-2′), 6.95 (1H, d, *J* = 2.9 Hz, H-2): ^13^C-NMR (acetone-*d*_6_, 150 MHz, ppm) δ 24.4, 24.4, 24.7, 24.7, 25.7, 25.8, 34.9, 36.3, 36.6, 38.8, 51.6, 52.2, 52.4, 71.0, 71.6, 72.6, 73.7, 76.3, 78.9, 79.5, 111.4, 111.6, 131.5, 133.2, 134.8, 138.8, 165.8, 167.2; HRMS *m*/*z* calcd. for C_28_H_37_O_10_^35^Cl (M)^+^, 568.2075; found, 568.2074.

### 4.18. Synthesis of (+)-***20***

Using the same procedure described for (−)-**20**, (+)-**20** (17.0 mg, 22%) was prepared from (+)-**8** (78.8 mg, 0.14 mmol). (+)-**20**: White amorphous solid; [α]${}_{D}^{20}$ +62.1 (*c* 0.355, CHCl_3_); IR (liquid film) ν_max_ 3423 (OH), 1722 (C=O), 1656 (C=C) cm^−1^; ^1^H-NMR (CDCl_3,_ 300 MHz, ppm) δ 1.20–2.00 (20H, m), 3.70–3.80 (1H, m, H-5′), 3.77 (3H, s, COOMe), 3.86 (3H, s, COOMe), 4.34 (1H, t, *J* = 4.6 Hz, H-5), 4.42–4.46 (1H, m, H-4′), 4.55 (1H, br d, *J* = 6.9 Hz, H-6′), 4.61 (1H, dd, *J* = 5.5, 3.3 Hz, H-3′), 4.77 (1H, dd, *J* = 7.5, 2.3 Hz, H-3), 4.88 (1H, dd, *J* = 7.5, 4.3 Hz, H-4), 5.06 (1H, br s, OH), 5.07 (1H, d, *J* = 4.8 Hz, H-6), 6.78 (1H, dd, *J* = 3.4, 0.9 Hz, H-2′), 6.98 (1H, d, *J* = 2.3 Hz, H-2); ^13^C-NMR (CDCl_3_, 75 MHz, ppm) δ 23.5, 23.5, 23.8, 23.8, 24.9, 25.0, 33.1, 35.0, 36.0, 37.5, 50.5, 52.2, 52.5, 68.6, 70.6, 72.1, 72.3, 74.8, 74.8, 78.9, 111.1, 112.0, 129.2, 129.8, 138.6, 139.5, 165.1, 166.8; HRMS *m*/*z* calcd. for C_28_H_37_O_10_^35^Cl (M)^+^, 568.2075; found, 568.2079.

### 4.19. Synthesis of (+)-***6***

Using the same procedure described for (−)-**6**, (+)-**6** (11.3 mg, 90%) was prepared from (+)-**20** (17.4 mg, 0.031 mmol). (+)-**6**: white crystal; [α]${}_{D}^{25}$ +73.3 (*c* 0.085, EtOH); IR (KBr) ν_max_ 3435 (OH), 1713 (C=O), 1643 (C=C) cm^−1^; ^1^H-NMR (acetone-*d*_6_, 600 MHz, ppm) δ 3.74–3.78 (1H, m, H-5′), 3.788 (3H, s, COOMe), 3.789 (3H, s, COOMe), 4.07 (1H, br s, H-4′), 4.20–4.30 (4H, m, OH, H-4′, H-3, H-3′), 4.36 (1H, br dd, *J* = 1.4, 1.2 Hz, H-5), 4.53 (1H, d, *J* = 4.1 Hz, H-6′), 5.24 (1H, d, *J* = 2.9 Hz, H-6), 5.40 (1H, br s, OH), 5.66 (1H, br d, *J* = 7.0 Hz, OH), 6.74 (1H, s, H-2′), 7.01 (1H, d, *J* = 4.2 Hz, H-2); ^13^C-NMR (acetone-*d*_6_, 150 MHz, ppm) δ 52.46 (CH_3_), 52.50 (CH_3_), 53.1 (CH_2_), 65.6 (CH_2_), 66.7 (CH_2_), 69.2 (CH_2_), 70.4 (CH_2_), 72.4 (CH_2_), 77.1 (CH_2_), 85.5 (CH_2_), 129.2 (C), 129.9 (C), 143.2 (CH_2_), 143.5 (CH_2_), 166.1 (Cq), 166.9 (Cq); HRMS *m*/*z* calcd. for C_16_H_22_O_10_^35^Cl (M)^+^, 408.0823; found, 408.0819.

### 4.20. Synthesis of (+)-***21***

Using the same procedure described for (−)-**21**, (+)-**21** (0. 9 mg, 63%) was prepared from (+)-**8** (20.0 mg, 0.035 mmol). (+)-**21**: oil; [α]${}_{D}^{20}$ −40.5 (*c* 0.035, EtOH); IR (liquid film) ν_max_ 3389 (OH), 1721 (C=O), 1653 (C=C) cm^−1^; ^1^H-NMR (acetone-*d*_6_, 300 MHz, ppm) δ 3.72 (3H, s, COOMe), 3.80 (3H, s, COOMe), 4.00 (1H, br s, H-4′), 4.09–4.20 (2H, m, H-3, H-4), 4.20–4.30 (1H, m, H-5, H-6′), 4.46 (1H, br s, H-3′), 4.50 (1H, br s, H-5′), 5.23 (1H, d, *J* = 2.6 Hz, H-6), 6.79 (1H, s, H-2′), 7.01 (1H, br d, *J* = 3.5 Hz, H-2); ^13^C-NMR (acetone-*d*_6_, 75 MHz, ppm) δ 52.3 (CH_3_), 52.5 (CH_3_), 52.9 (CH_2_), 65.7 (CH_2_), 65.8 (CH_2_), 66.7 (CH_2_), 69.1 (CH_2_), 70.1 (CH_2_), 76.7 (CH_2_), 83.5 (CH_2_), 129.5 (C), 129.5 (C), 142.5 (CH_2_), 142.7 (CH_2_), 166.0 (Cq), 166.9 (Cq); HRMS *m*/*z* calcd. for C_16_H_21_O_10_^35^Cl (M)^+^, 408.0823; found, 408.0819.

### 4.21. Synthesis of (−)-***24***

Using the same procedure described for (+)-**24**, (−)-**24** (0.25 g, 35%) was prepared from (−)-**9** (0.45 g, 1.5 mmol) and (−)-**10** (0.33 g, 1.2 mmol). (−)-**24**: amorphous solid; *R_f_* 0.14 (Hexane–EtOAc, 3:1); [α]${}_{D}^{18}$ −126.47 (*c* 0.825, CHCl_3_); IR (liquid film) ν_max_ 3470 (OH), 1722 (C=O), 1658 (C=C) cm^−1^; ^1^H-NMR (acetone-*d*_6_, 600 MHz, ppm) δ 1.26–1.70 (20H, m), 3.78 (3H, s, COOMe), 3.80 (3H, s, COOMe), 4.03 (1H, br ddd, *J* = 5.0, 4.7, 4.4 Hz, H-5′), 4.22 (1H, dd, *J* = 6.1, 5.3 Hz, H-4′), 4.32 (1H, dd, *J* = 4.7, 3.8 Hz, H-5), 4.35 (1H, dd, *J* = 4.4, 0.9 Hz, H-6′), 4.55 (1H, d, *J* = 4.7 Hz, OH), 4.67 (1H, ddd, *J* = 6.1, 3.8, 1.1 Hz, H-3′), 4.69 (1H, dd, *J* = 6.4, 3.8 Hz, H-4), 4.78 (1H, ddd, *J* = 6.7, 3.0, 0.6 Hz, H-3), 5.16 (1H, d, *J* = 4.7 Hz, H-6), 6.63 (1H, d, *J* = 3.8 Hz, H-2′), 6.93 (1H, d, *J* = 2.9 Hz, H-2); ^13^C-NMR (acetone-*d*_6_, 150 MHz, ppm) δ 24.6 (2C), 24.8 (2C), 25.8, 25.9, 35.6, 36.0, 36.9, 38.4, 52.2, 52.4, 52.7, 70.8, 71.1, 71.2, 72.6, 76.2, 77.3, 79.5, 111.2, 111.3, 131.4, 133.2, 135.0, 138.9, 165.9, 167.3; HRMS *m*/*z* calcd. for C_28_H_37_O_10_^35^Cl (M)^+^, 568.2075; found, 568.2079.

### 4.22. Synthesis of (−)-***25***

Using the same procedure described for (–)-**21**, (+)-**21** (0.066 g, 39%) was prepared from (−)-**24** (0.17 g, 0.30 mmol). (−)-**25**: oil; *R_f_* 0.14 (Hexane–EtOAc, 3:1); [α]${}_{D}^{16}$ −43.3 (*c* 0.085, CH_2_Cl_2_); IR (liquid film) ν_max_ 3523 (OH), 1717 (C=O), 1653 (C=C) cm^−1^; ^1^H-NMR (acetone-*d_6_*, 600 MHz, ppm) δ 1.20–1.70 (20H, m), 3.79 (3H, s, COOMe), 3.81 (3H, s, COOMe), 3.78–3.81 (1H, m, H-5′), 4.36 (1H, dd, *J* = 5.3, 3.9 Hz, H-4′), 4.48 (1H, dd, *J* = 6.5, 3.5 Hz, H-4), 4.58 (1H, d, *J* = 5.0 Hz, H-6′), 4.75 (1H, d, *J* = 3.9 Hz, H-3′), 4.76 (1H, dd, *J* = 3.8, 0.9 Hz, H-5), 4.81 (1H, ddd, *J* = 6.7, 2.7, 0.9 Hz, H-3), 5.21 (1H, d, *J* = 5.3 Hz, H-6), 6.76 (1H, dd, *J* = 3.8, 0.9 Hz, H-2′), 6.88 (1H, d, *J* = 2.7 Hz, H-2); ^13^C-NMR (acetone-*d_6_*, 150 MHz, ppm) δ 24.5, 24.6, 24.6, 24.9, 25.8, 25.9, 35.1, 35.7, 36.7, 37.7, 52.3, 52.6, 53.3, 69.0, 71.4, 72.7, 72.8, 74.6, 75.2, 79.7, 111.1, 111.8, 131.1, 131.2, 138.2, 140.0, 166.4, 166.8; HRMS *m*/*z* calcd. for C_28_H_37_O_10_^35^Cl (M)^+^, 568.2075; found, 568.2076.

### 4.23. Synthesis of (−)-***22***

Using the same procedure described above for (−)-**22**, (+)-**22** (5.9 mg, 33%) was prepared from (−)-**25** (25 mg, 0.043 mmol). (−)-**22**: oil; *R_f_* 0.3 (MeOH-CH_2_Cl_2_, 1:9); [α]${}_{D}^{19}$ −5.0 (*c* 0.225, EtOH); IR (liquid film) ν_max_ 3391 (OH), 1716 (C=O), 1651 (C=C) cm^−1^; ^1^H-NMR (acetone-*d*_6_, 600 MHz, ppm) δ 3.68 (1H, br d, *J* = 10.0 Hz, OH), 3.76–3.82 (1H, m, H-5), 3.78 (3H, s, COOMe), 3.80 (3H, s, COOMe), 3.86 (1H, br d, *J* = 10.3 Hz, OH), 3.92 (1H, br d, *J* = 7.3 Hz, OH), 3.99 (1H, s, H-4′), 4.01–4.08 (2H, m, H-4, OH), 4.12–4.18 (2H, m, H-3, OH), 4.26 (1H, s, H-3′), 4.47 (1H, dd, *J* = 2.9, 2.4 Hz, H-5), 4.57 (1H, d, *J* = 4.1 Hz, H-6′), 5.38 (1H, d, *J* = 3.3 Hz, H-6), 6.80 (1H, dd, *J* = 2.3, 1.2 Hz, H-2′), 7.04 (1H, d, *J* = 5.0 Hz, H-2); ^13^C-NMR (acetone-*d*_6_, 150 MHz, ppm) δ 52.40, 52.43, 53.87, 65.89, 66.20, 69.09, 69.33, 72.18, 74.01, 83.18, 129.93, 130.07, 142.56, 143.34, 165.95, 167.33; HRFABMS *m*/*z* calcd. for C_16_H_22_O_10_^35^Cl (M + H)^+^, 409.0901; found, 409.0900.

### 4.24. Synthesis of (−)-***23***

Using the same procedure described above for (+)-**23**, (−)-**23** (7.6 mg, 50%) was prepared from (−)-**24** (21.3 mg, 0.038 mmol). (−)-**23**: white crystals; *R_f_* 0.11 (MeOH-CH_2_Cl_2_, 1:9); [α]${}_{D}^{20}$ −75.3 (*c* 0.095, EtOH); IR (liquid film) ν_max_ 3349 (OH), 1716 (C=O), 1593 (C=C) cm^−1^; ^1^H-NMR (acetone-*d*_6_, 600 MHz, ppm) δ 3.75 (1H, dd, *J* = 6.7, 4.1 Hz, H-4′), 3.79 (6H, s, COOMe), 4.02 (1H, d, *J* = 6.8, 4.1 Hz, H-5′), 4.10 (1H, br s, H-4), 4.21 (1H, br s, H-3), 4.36 (1H, d, *J* = 4.1 Hz, H-6′), 4.37 (1H, dd, *J* = 4.1, 3.8 Hz, H-3′), 4.43 (1H, dd, *J* = 3.6, 2.0 Hz, H-5), 5.24 (1H, d, *J* = 3.5 Hz, H-6), 6.79 (1H, d, *J* = 3.5 Hz, H-2′), 6.99 (1H, d, *J* = 4.4 Hz, H-2); ^13^C-NMR (acetone-*d*_6_, 150 MHz, ppm) δ 52.4, 52.5, 54.5, 66.3, 66.4, 66.6, 70.6, 71.9, 77.2, 83.7, 129.8, 130.8, 141.3, 1412.7, 166.1, 167.1; HRMS *m*/*z* calcd. for C_16_H_21_O_10_^35^Cl (M)^+^, 408.0823; found, 408.0821.

### 4.25. Glycosidase Assays of Synthesized Compounds

***Assay of α-Glucosidase inhibition***: The assay reaction mixture consisted of 0.1 M acetate buffer (pH 5.0, 45 μL), *p*-nitrophenyl α-d-glucopyranoside solution (25 μL, 250 mM), and α-glucosidase solution (25 μL, a stock solution of 1.0 mg/mL in 50 mM Tris-HCl-buffer at pH 7.8 diluted 200-fold with 10 mM phosphate buffer at pH 7.0 just prior to the assay), with the test samples **6**, **21**–**23**, or DNJ (25 μL solution, concentration range 0.1–20 mg/mL). After incubation for 20 min at 37 °C, the reaction was interrupted by the addition of 0.5 M sodium carbonate (100 μL). The amount of *p*-nitrophenol liberated was measured colorimetrically at 400 nm (optical density at 400 nm: ODtest). The inhibition rates (%) were calculated using the formula 100 − 100 × (ODtest − ODblank)/(control ODtest − control ODblank) and the IC_50_ values were obtained from the inhibition curves. Assays for β-glucosidase and α-mannosidase were carried out as outlined above using *p*-nitrophenyl β-d-glucopyranoside and α-d-mannopyranoside as the substrates. The IC_50_ values are shown in Table 1.

Assays on β-glucosidase and α-mannnosidase inhibition of synthesized carbadisaccharides **6** and **21**–**23** were carried out in a similar fashion.

## Figures and Tables

**Table 1:** Evaluation of anti-glycosidase activity of the synthesized compounds.

Compound	IC_50_ (M)		
	α-Glucosidase ^a^	β-Glucosidase ^b^	α-Mannosidase ^c^
(−)-pericosine E (**6**)	1.5 × 10^−3^	NI ^d^	NI
(−)-**21**	1.8 × 10^−3^	NI	NI
(+)-pericosine E (**6**)	3.1 × 10^−5^	NI	NI
(+)-**21**	3.2 × 10^−3^	NI	NI
(+)-**22**	NI	NI	NI
(+)-**23**	5.1 × 10^−3^	NI	NI
(−)-**22**	1.2 × 10^−5^	NI	NI
(−)-**23**	NI	NI	NI
Deoxynojirimycin (DNJ)	6.3 × 10^−4^	5.5 × 10^−4^	NI
Deoxymannojirimycin	NI	NI	6.4 × 10^−4^

^a^ Yeast; ^b^ Sweet almond; ^c^ Jack bean; ^d^ No inhibition.

## Supplementary Materials

The following are available online at www.mdpi.com/1660-3397/15/1/22/s1. Figure S1: ^1^H-NMR spectrum of compound (−)-**21** in acetone-*d*_6_ (600 MHz), Figure S2: 13C-NMR spectrum of compound (−)-21 in acetone-*d*_6_ (150 MHz), Figure S3: 1H-NMR spectrum of compound (+)-24 in acetone-*d*_6_ (600 MHz), Figure S4: 13C-NMR spectrum of compound (+)-24 in acetone-*d*_6_ (150 MHz), Figure S5: 1H-NMR spectrum of compound (+)-25 in acetone-*d*_6_ (400 MHz), Figure S6: 13C-NMR spectrum of compound (+)-25 in acetone-*d*_6_ (100 MHz), Figure S7: 1H-NMR spectrum of compound (+)-22 in acetone-*d*_6_ (400 MHz), Figure S8: 13C-NMR spectrum of compound (+)-22 in acetone-*d*_6_ (100 MHz), Figure S9: 1H-NMR spectrum of compound (+)-23 in acetone-*d*_6_ (300 MHz), Figure S10: 13C-NMR spectrum of compound (+)-23 in acetone-*d*_6_ (75 MHz), Figure S11: 1H-NMR spectrum of compound (+)-8 in acetone-*d*_6_ (600 MHz), Figure S12: 13C-NMR spectrum of compound (+)-8 in acetone-*d*_6_ (150 MHz), Figure S13: 1H-NMR spectrum of compound (+)-20 in CDCl3 (300 MHz), Figure S14: 13C-NMR spectrum of compound (+)-20 in CDCl3 (75 MHz), Figure S15: 1H-NMR spectrum of compound (+)-6 in acetone-*d*_6_ (600 MHz), Figure S16: 13C-NMR spectrum of compound (+)-6 in acetone-*d*_6_ (150 MHz), Figure S17: 1H-NMR spectrum of compound (+)-21 in acetone-*d*_6_ (300 MHz), Figure S18: 13C-NMR spectrum of compound (+)-21 in acetone-*d*_6_ (75 MHz), Figure S19: 1H-NMR spectrum of compound (−)-24 in acetone-*d*_6_ (600 MHz), Figure S20: 13C-NMR spectrum of compound (−)-24 in acetone-*d*_6_ (150 MHz), Figure S21: 1H-NMR spectrum of compound (−)-25 in acetone-*d*_6_ (600 MHz), Figure S22: 13C-NMR spectrum of compound (−)-25 in acetone-*d*_6_ (150 MHz), Figure S23: 1H-NMR spectrum of compound (−)-22 in acetone-*d*_6_ (600 MHz), Figure S24: 13C-NMR spectrum of compound (−)-22 in acetone-*d*_6_ (150 MHz), Figure S25: 1H-NMR spectrum of compound (−)-23 in acetone-*d*_6_ (600 MHz), Figure S26: 13C-NMR spectrum of compound (−)-23 in acetone-*d*_6_ (150 MHz).
