# Supplementary material for: Synthesis of Natural O-Linked Carba-Disaccharides, (+)- and (−)-Pericosine E, and Their Analogues as α-Glucosidase Inhibitors
Source: Mar Drugs. 2017 Jan 23;15(1):22. doi: 10.3390/md15010022 (PMC5295242; doi:10.3390/md15010022)
Supplement: Supplementary file 1 [file marinedrugs-15-00022-s001.docx]

Supplementary Materials: Synthesis of Natural
*O*-Linked Carba-Disaccharides,
(+)- and (−)-Pericosine E, and Their Analogues as
α-Glucosidase Inhibitors

Yoshihide Usami, Koji Mizuki, Rikiya Kawahata, Makio Shibano, Atsuko Sekine,
Hiroki Yoneyama and Shinya Harusawa

^1^H- and ^13^C-NMR spectra of new compounds S-**2**–S-**27**.

| 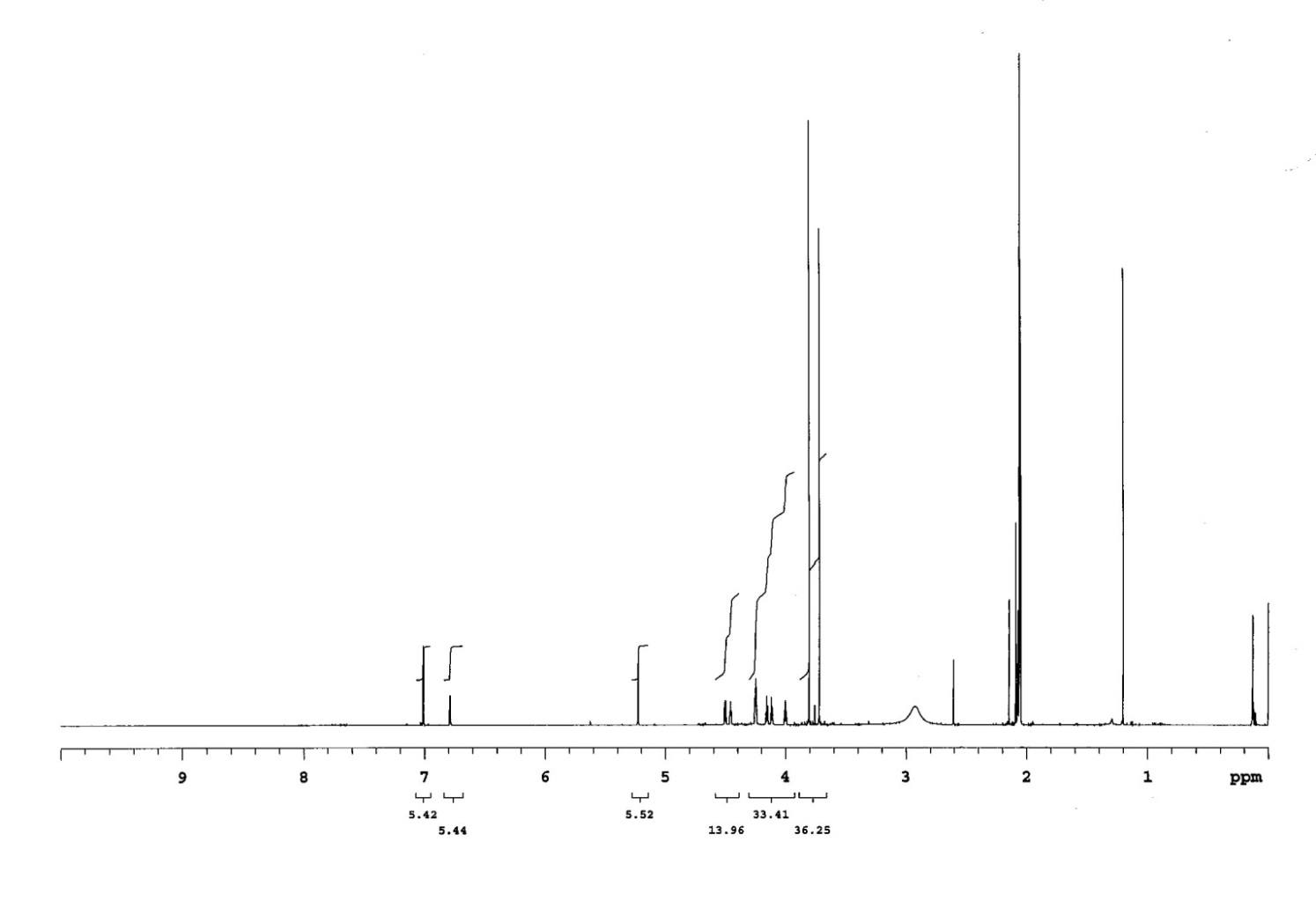 |
| --- |
| 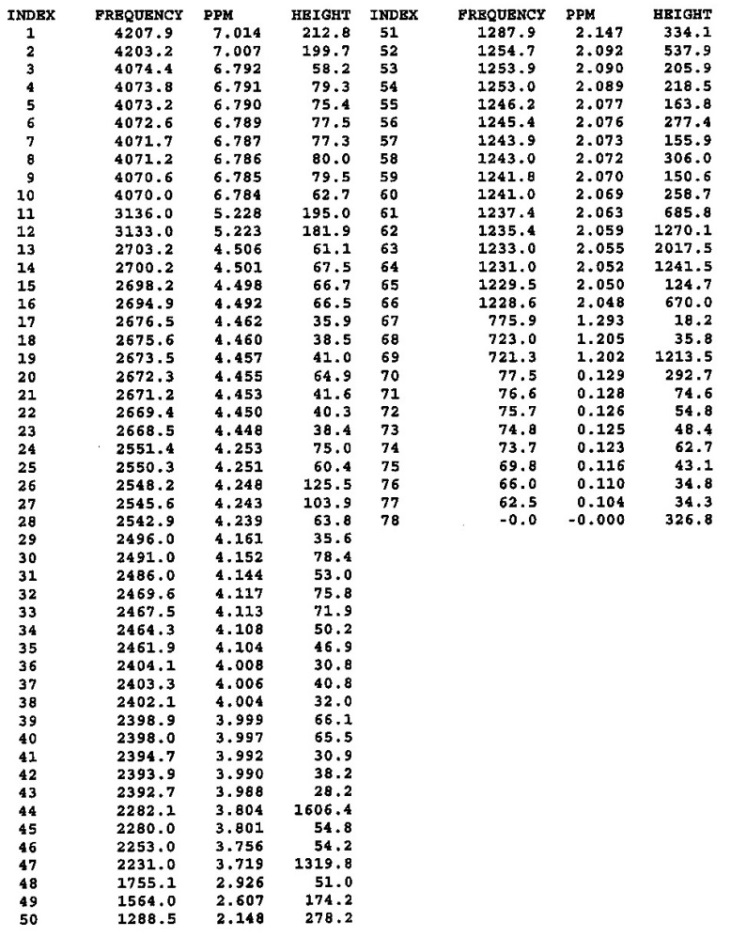 |

**Figure S1.** ^1^H-NMR spectrum of compound (−)-**21** in acetone-*d*_6_ (600 MHz).

| 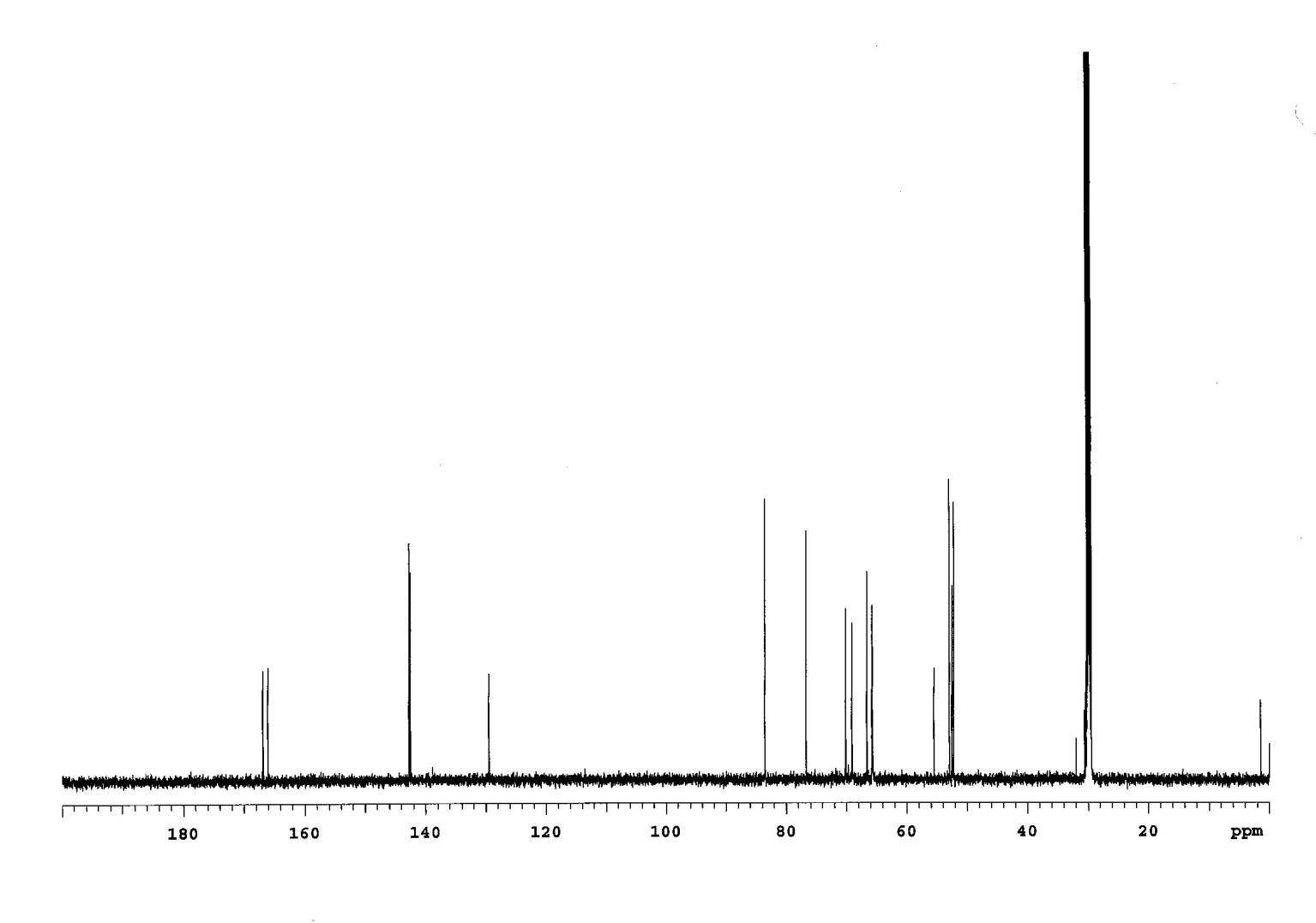 |
| --- |
| 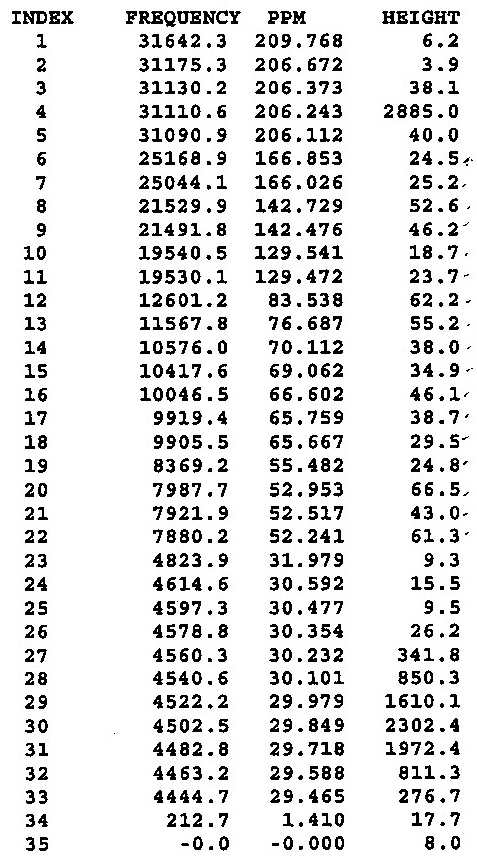 |

**Figure S2.** ^13^C-NMR spectrum of compound (−)-**21** in acetone-*d*_6_ (150 MHz).

| 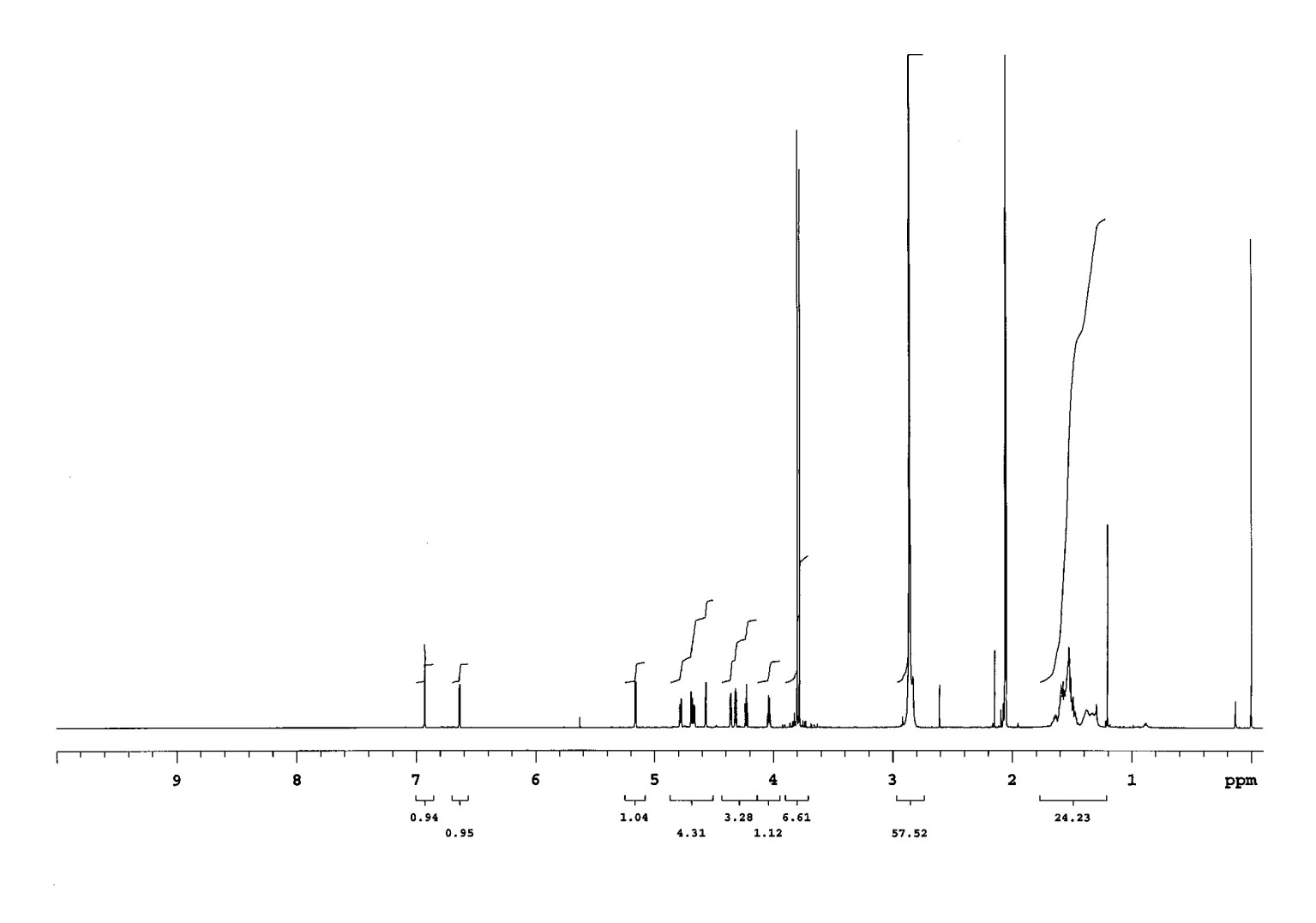 |
| --- |
| 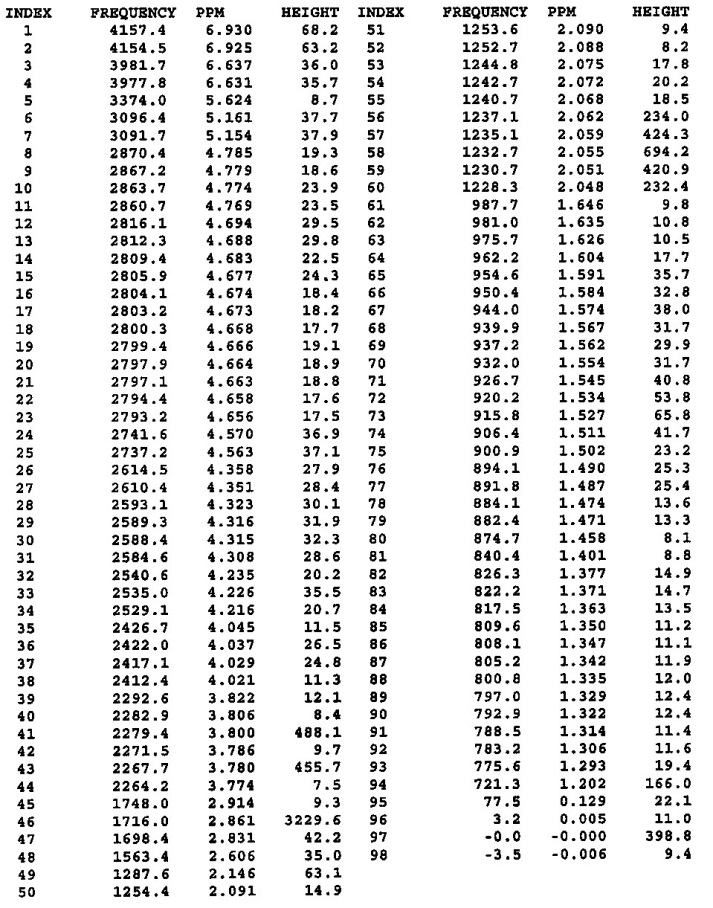 |

**Figure S3.** ^1^H-NMR spectrum of compound (+)-**24** in acetone-*d*_6_ (600 MHz).

| 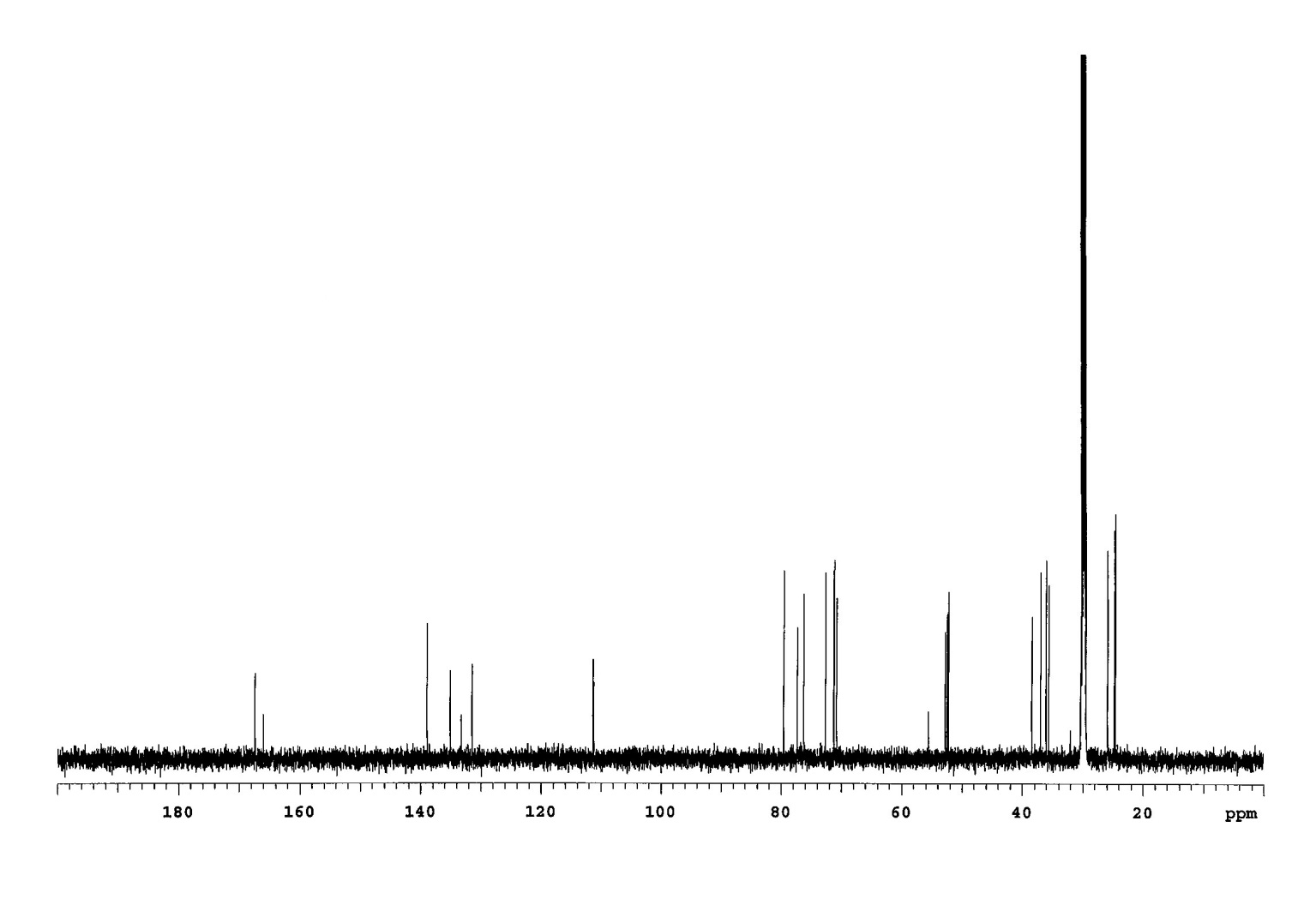 |
| --- |
| 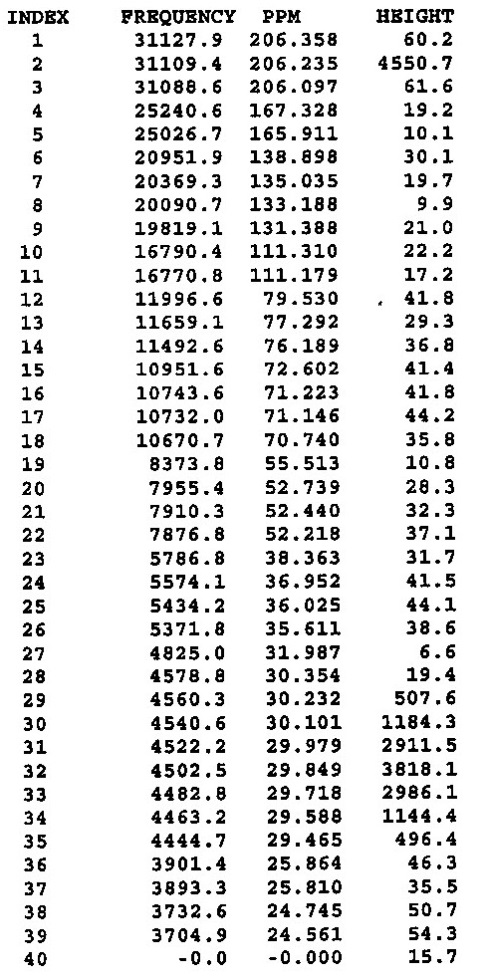 |

**Figure S4.** ^13^C-NMR spectrum of compound (+)-**24** in acetone-*d*_6_ (150 MHz).

| 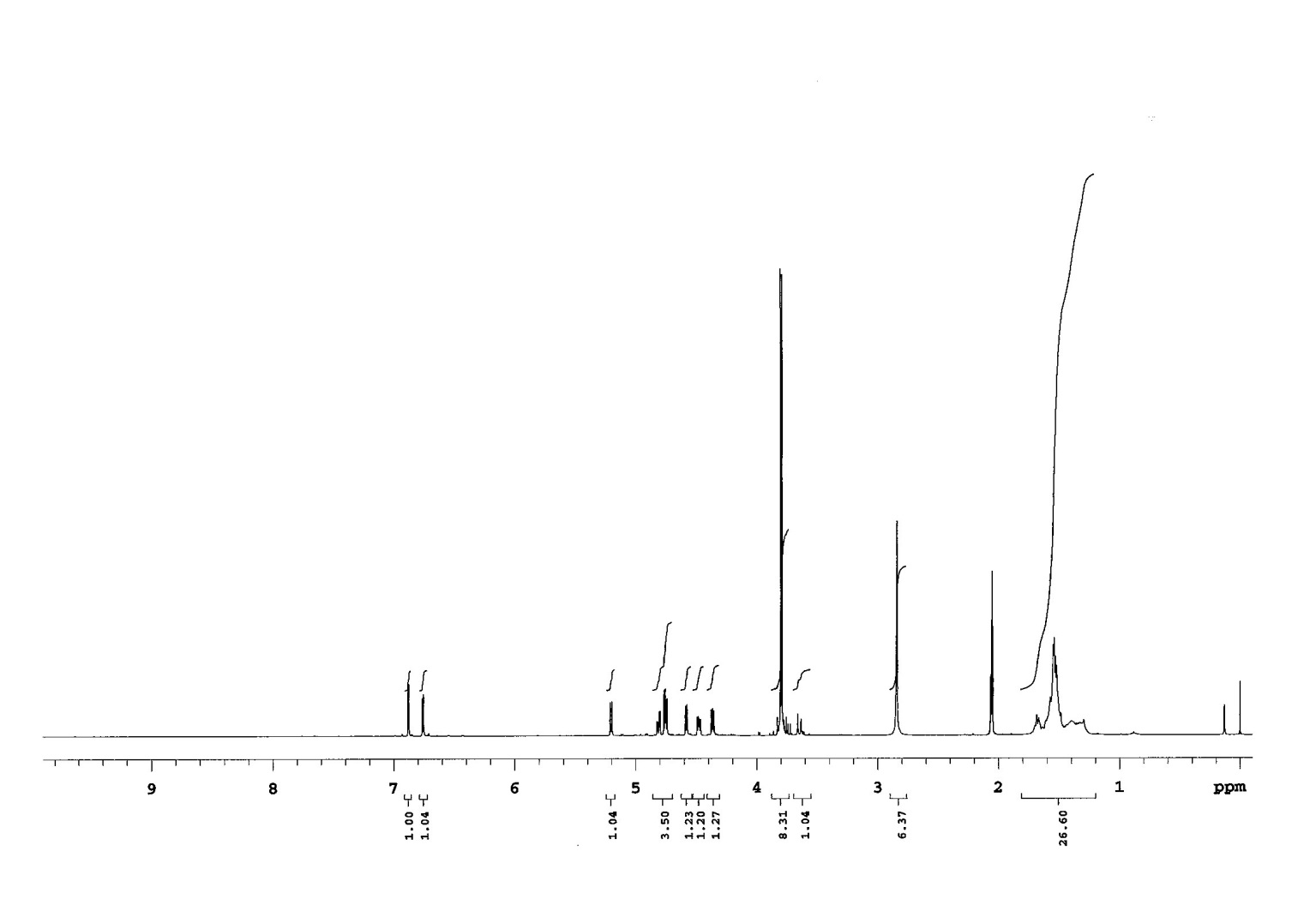 |
| --- |
| 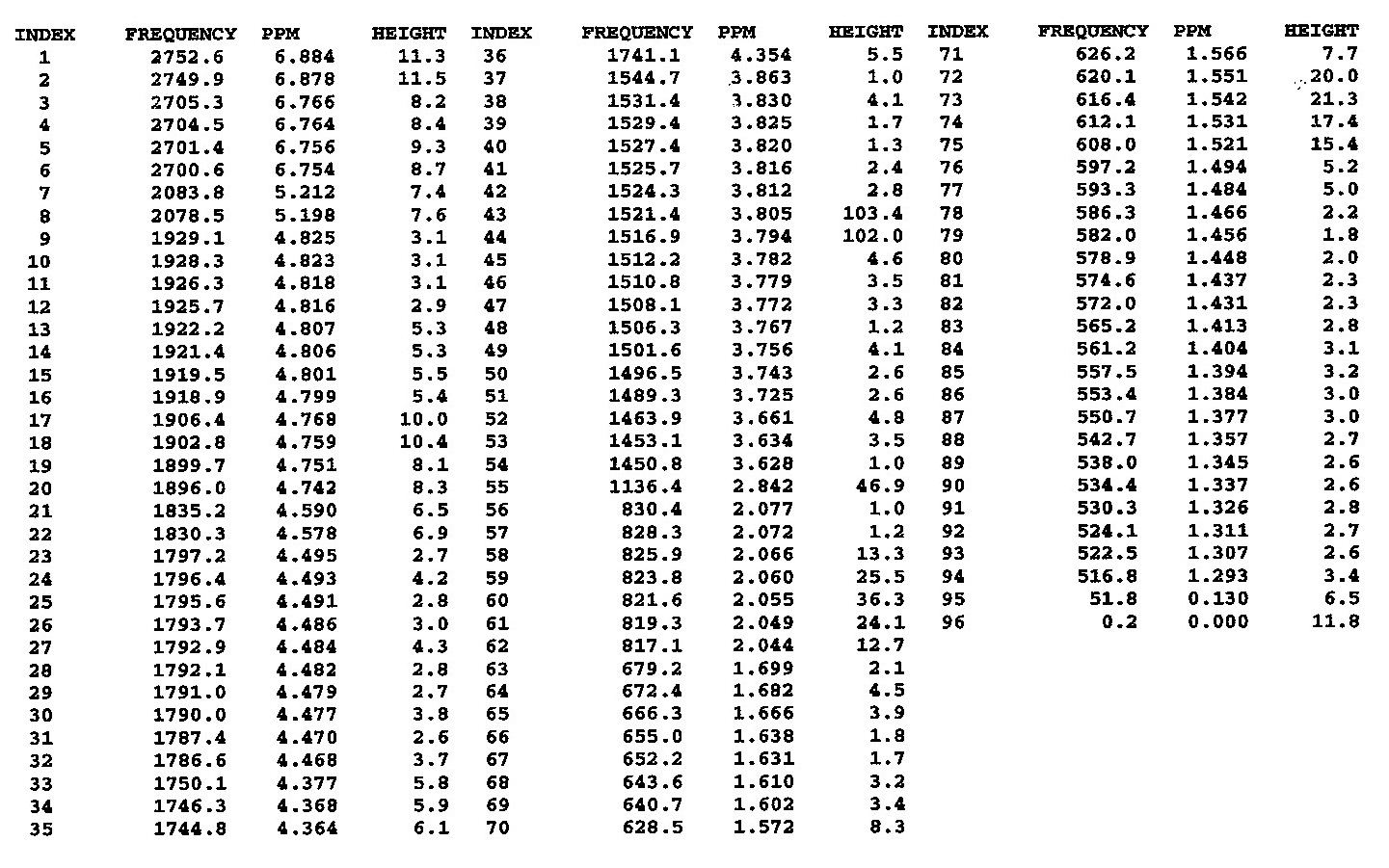 |

**Figure S5.** ^1^H-NMR spectrum of compound (+)-**25** in acetone-*d*_6_ (400 MHz).

| 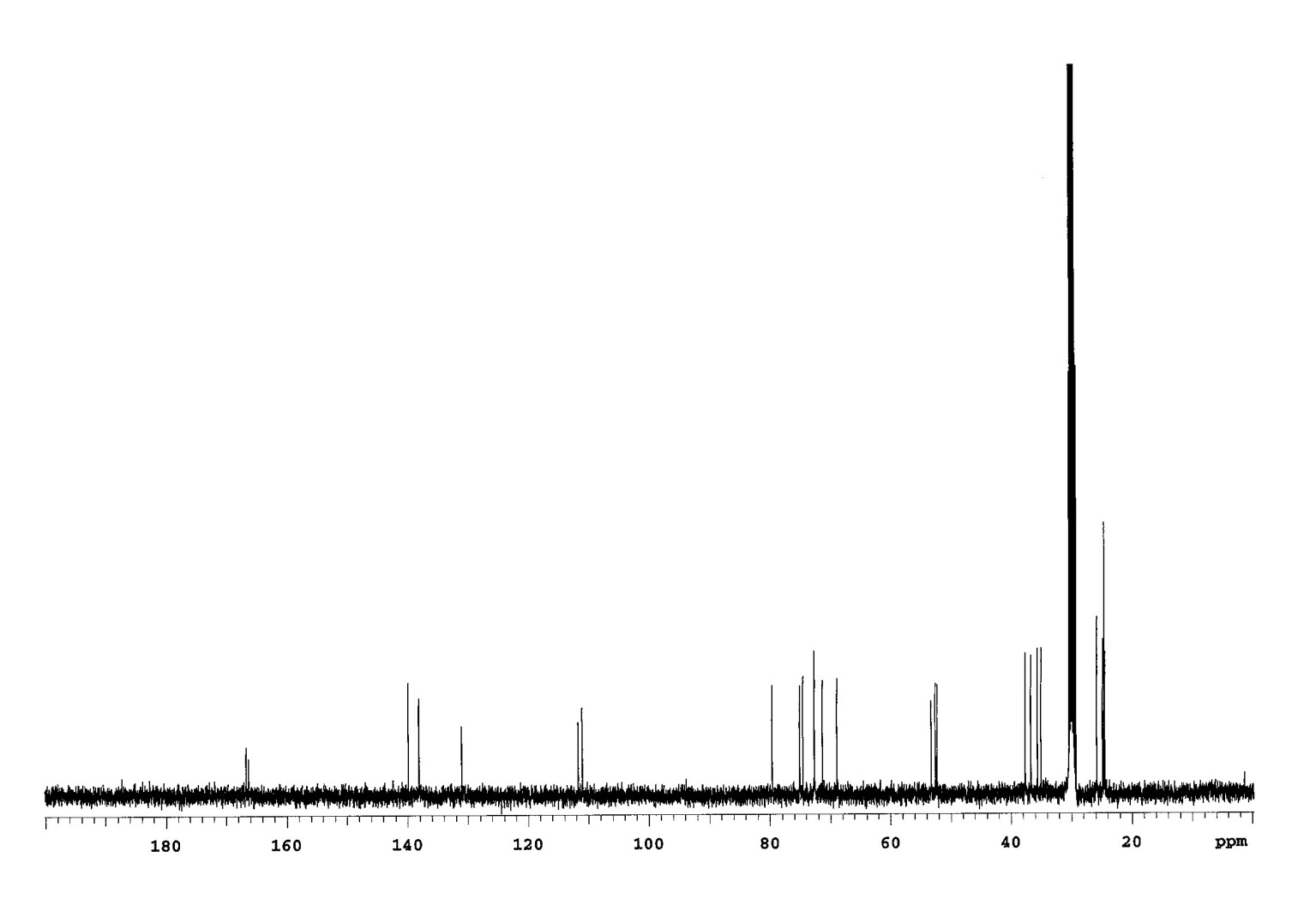 |
| --- |
| 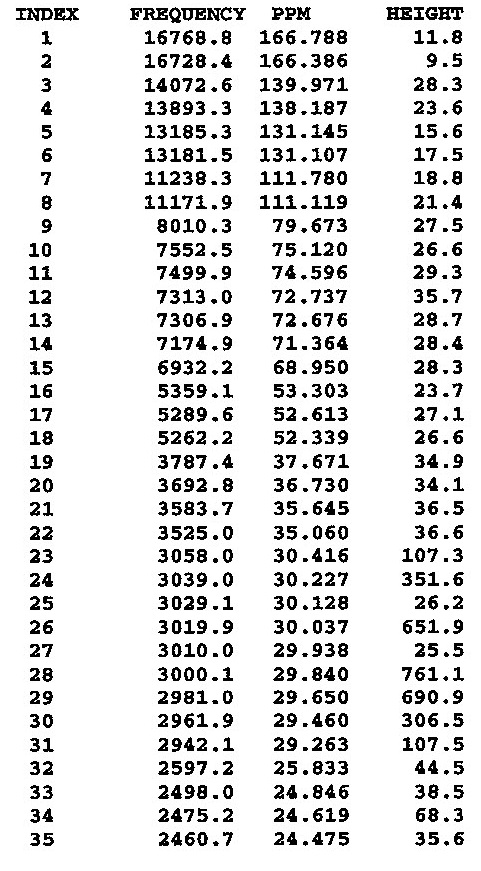 |

**Figure S6.** ^13^C-NMR spectrum of compound (+)-**25** in acetone-*d*_6_ (100 MHz).

| 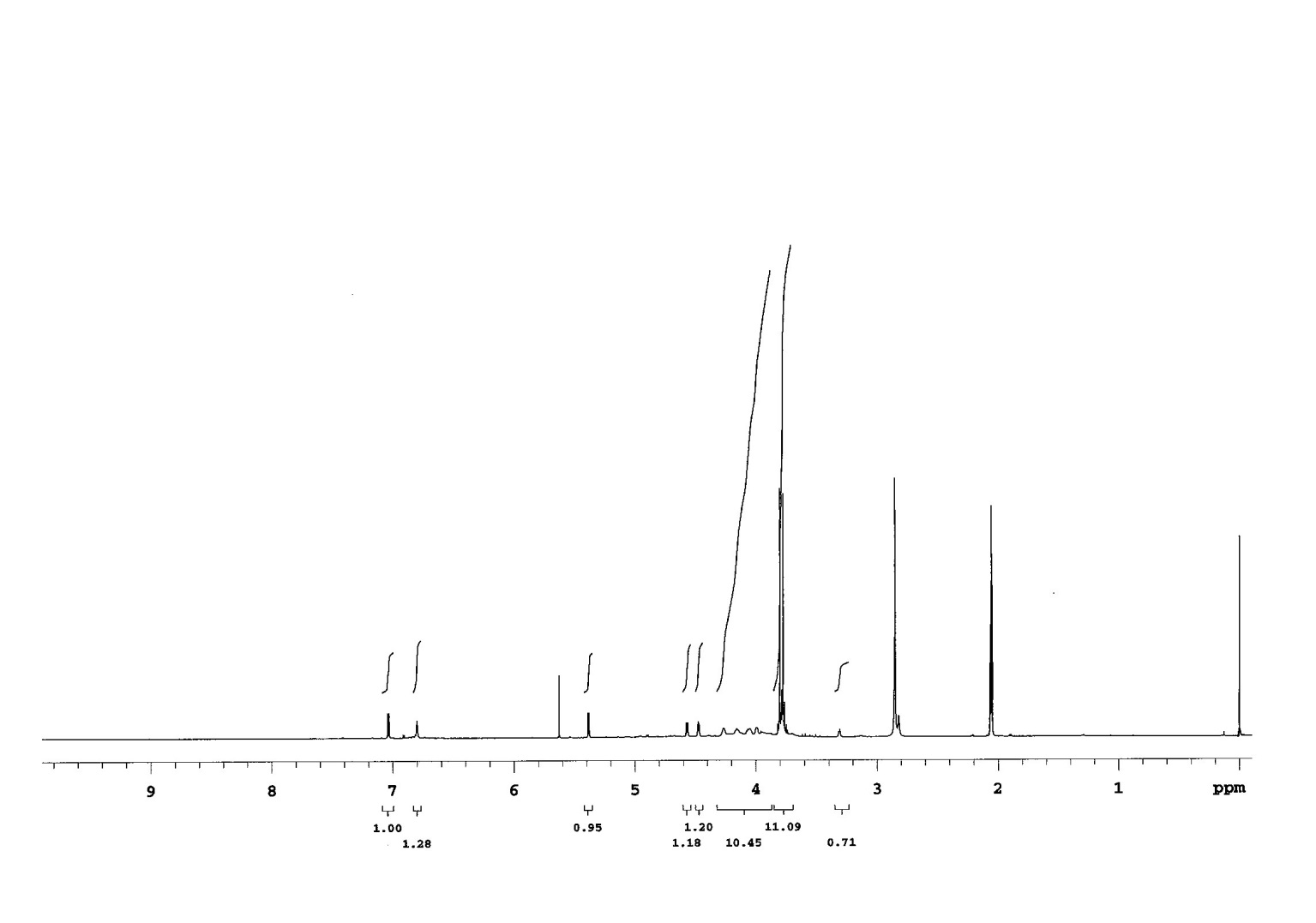 |
| --- |
| 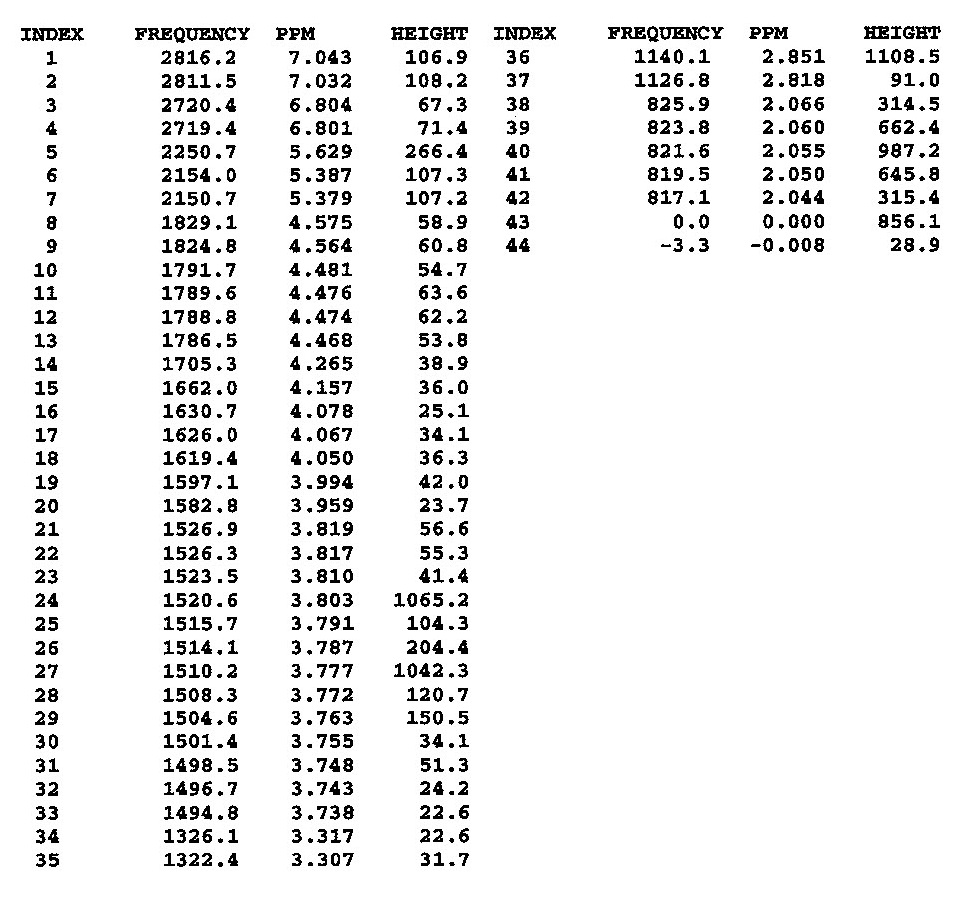 |

**Figure S7.** ^1^H-NMR spectrum of compound (+)-**22** in acetone-*d*_6_ (400 MHz).


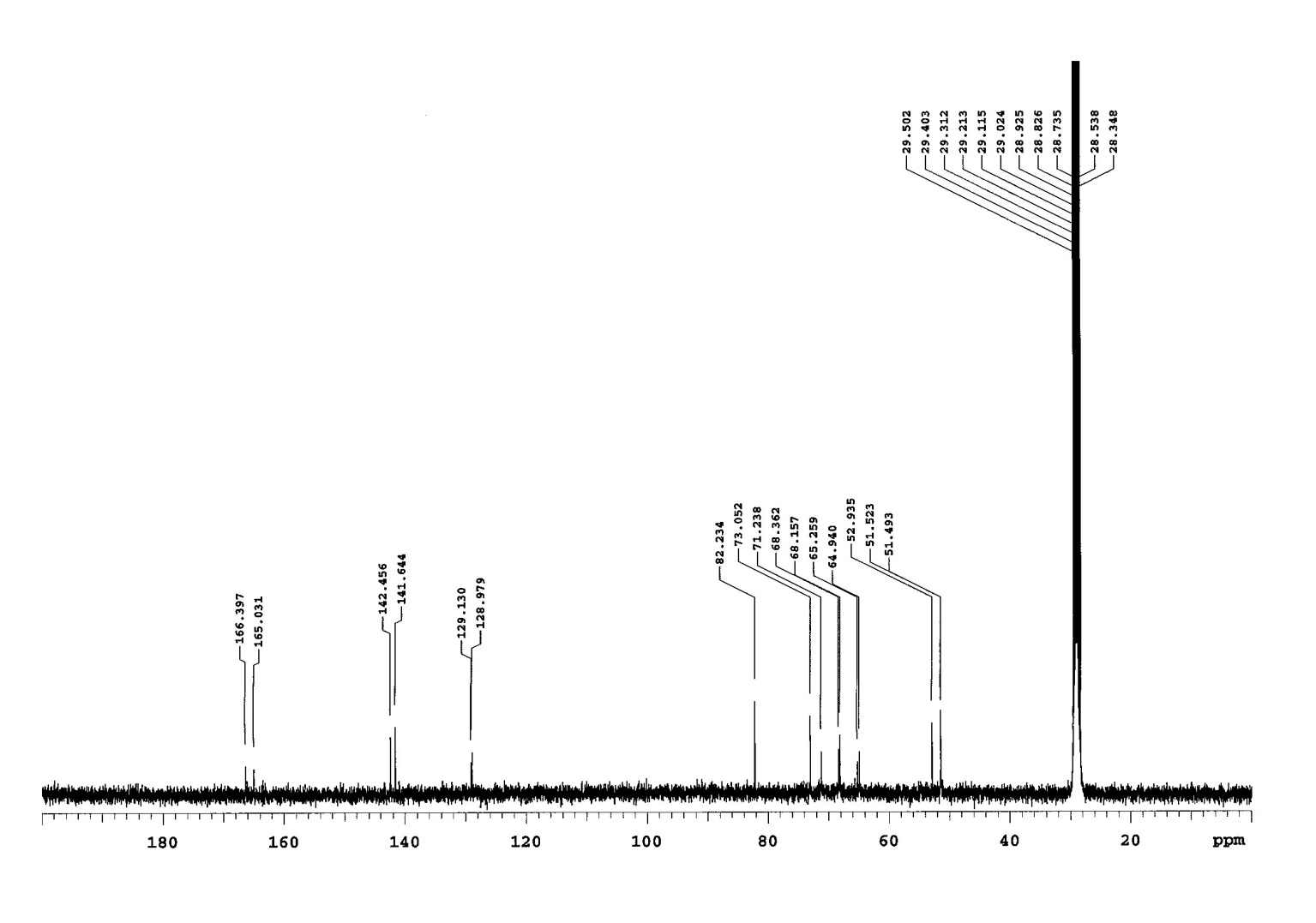


**Figure S8.** ^13^C-NMR spectrum of compound (+)-**22** in acetone-*d*_6_ (100 MHz).

| 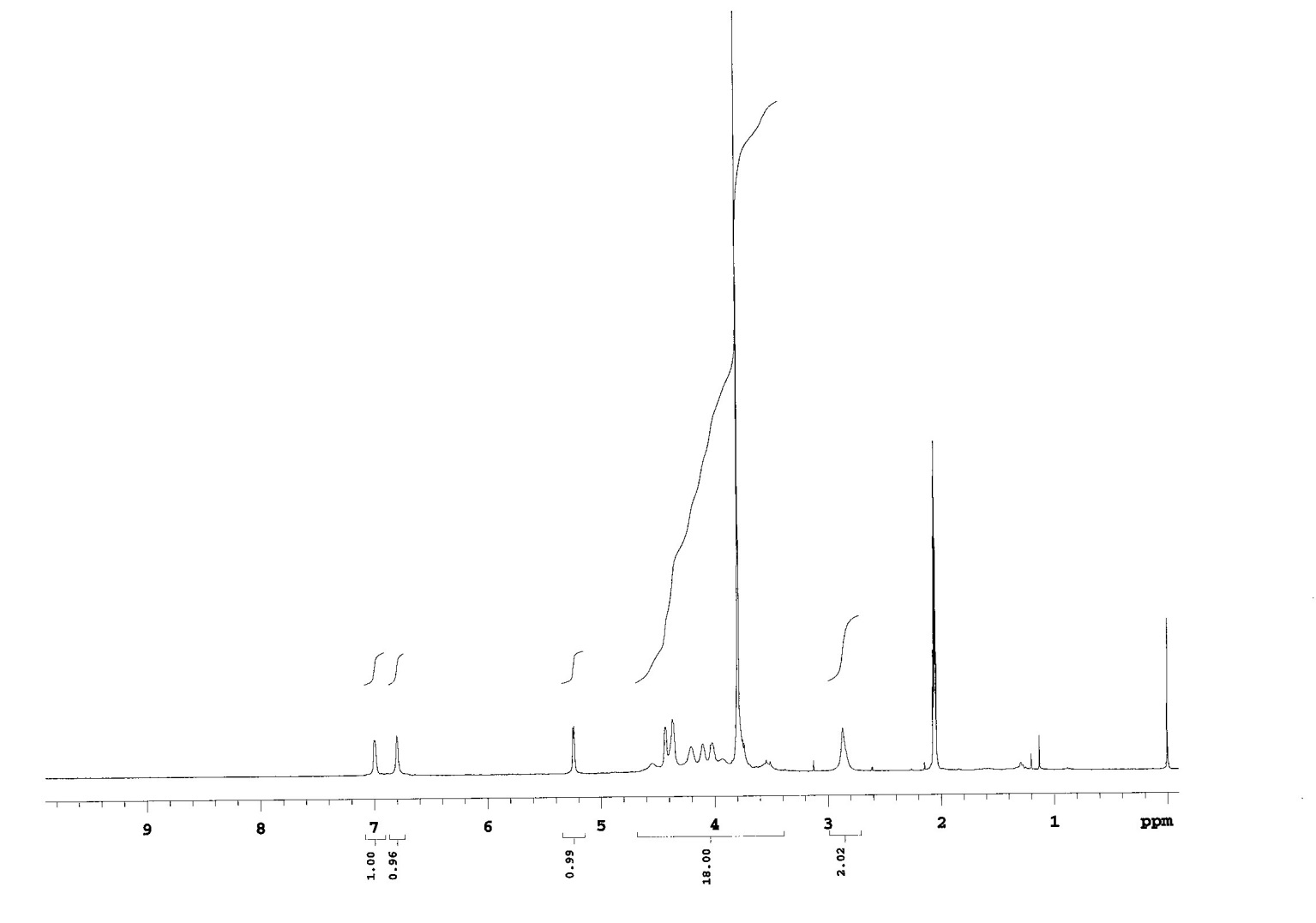 |
| --- |
| 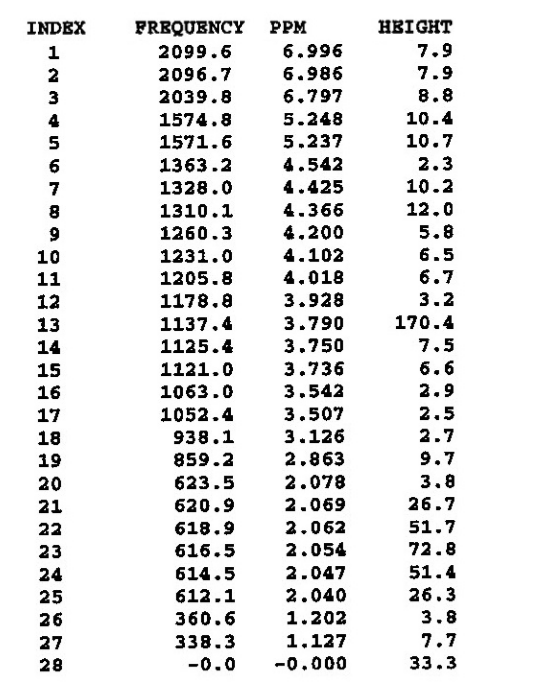 |

**Figure S9.** ^1^H-NMR spectrum of compound (+)-**23** in acetone-*d*_6_ (300 MHz).

| 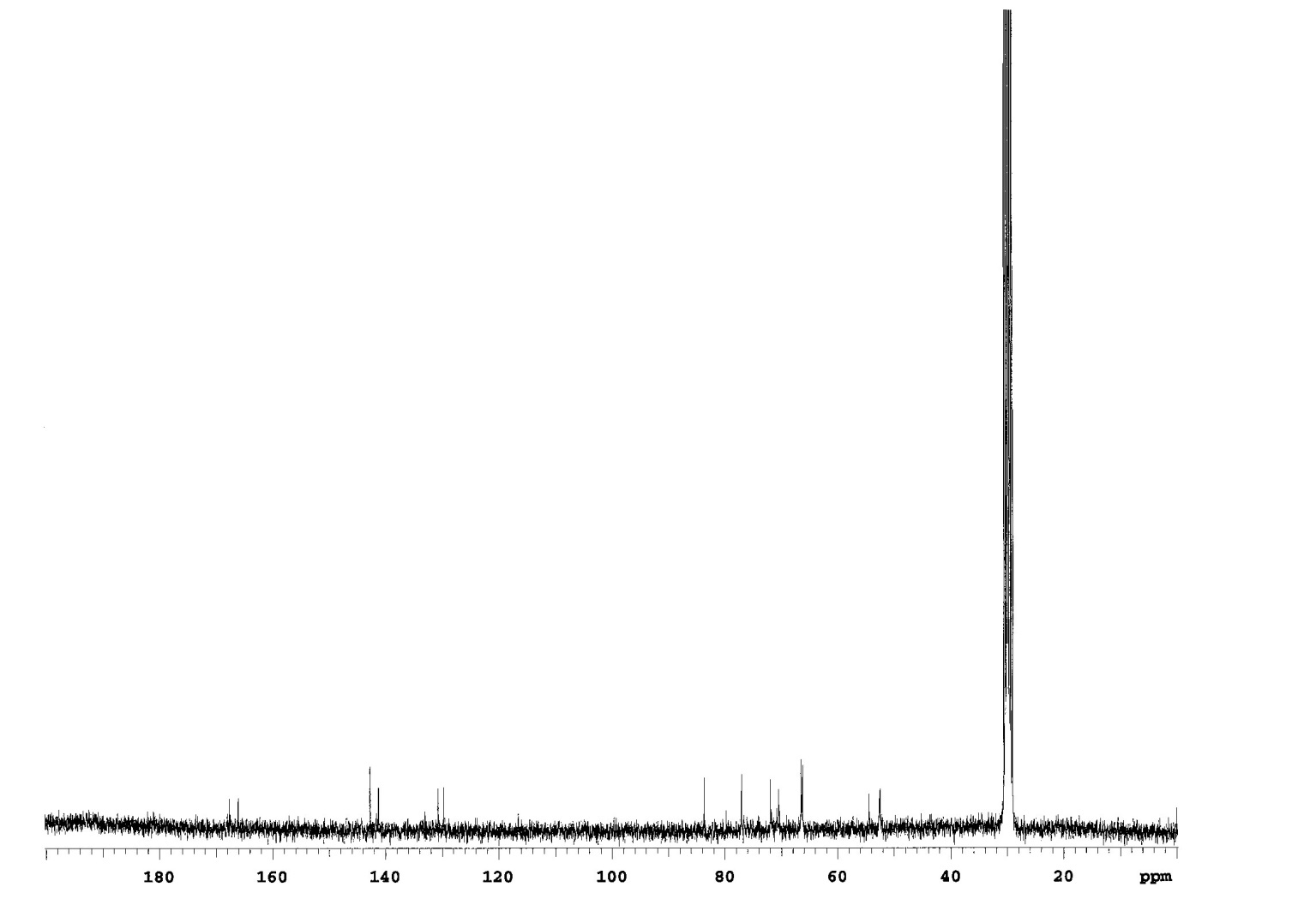 |
| --- |
| 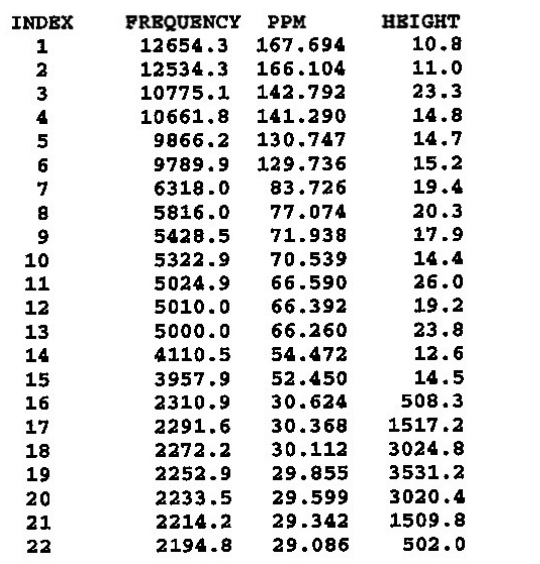 |

**Figure S10.** ^13^C-NMR spectrum of compound (+)-**23** in acetone-*d*_6_ (75 MHz).

| 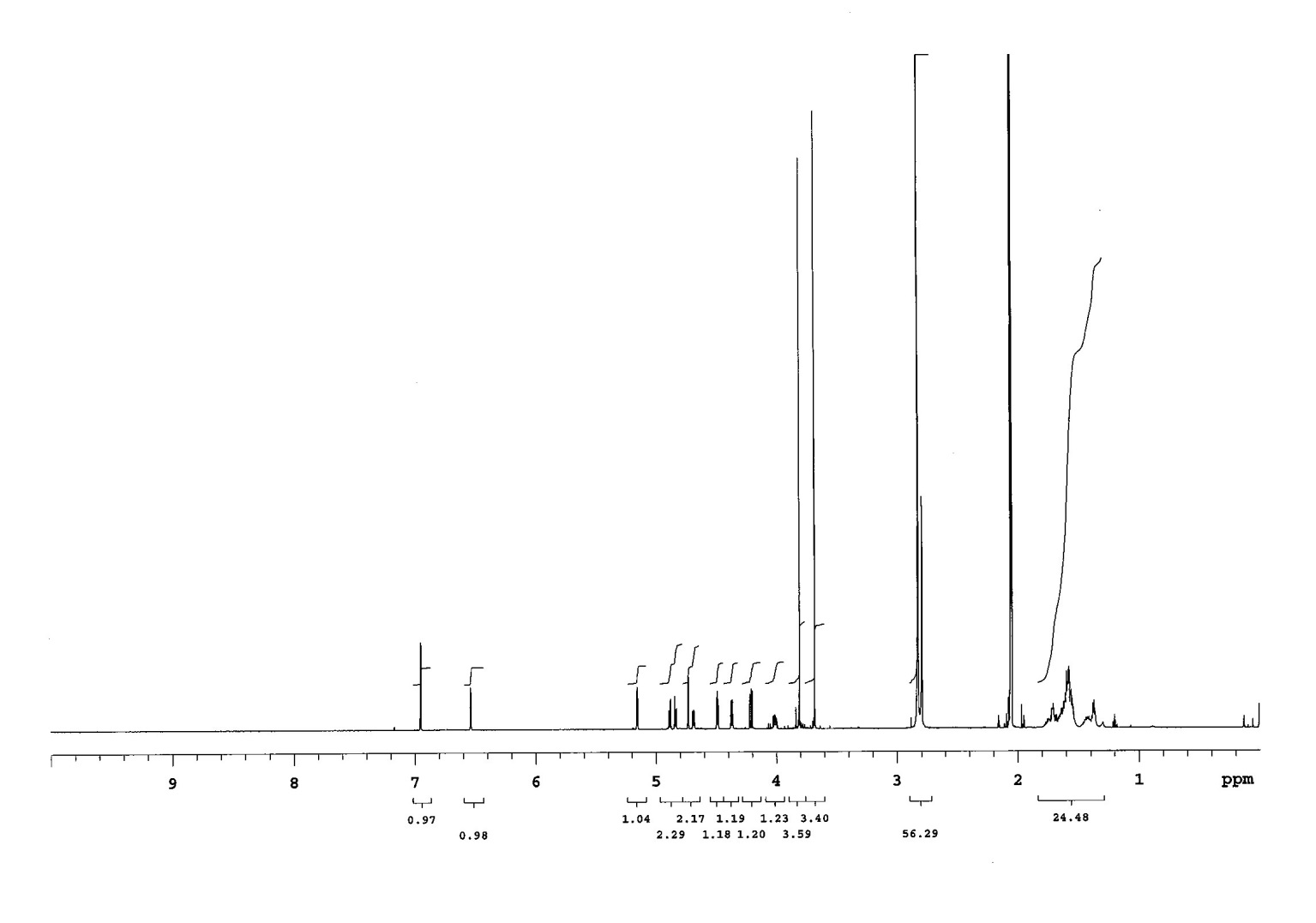 |
| --- |
| 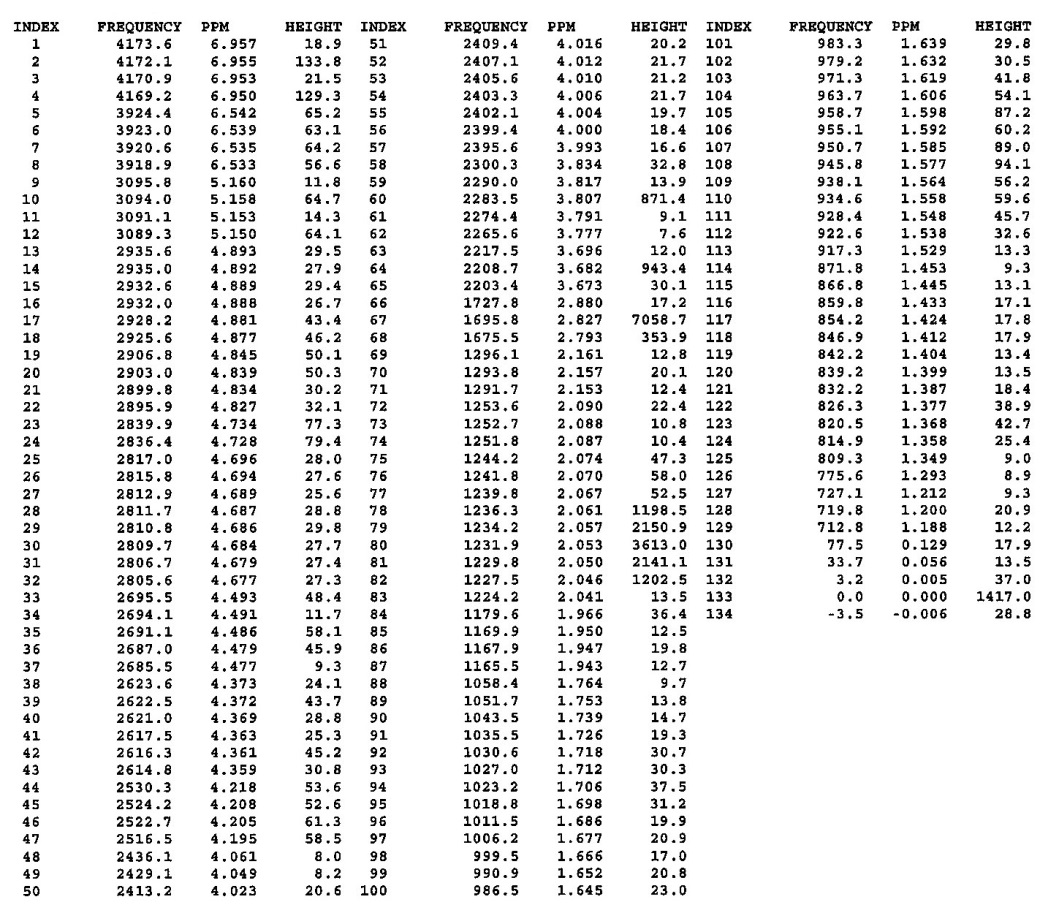 |

**Figure S11.** ^1^H-NMR spectrum of compound (+)-**8** in acetone-*d*_6_ (600 MHz).

| 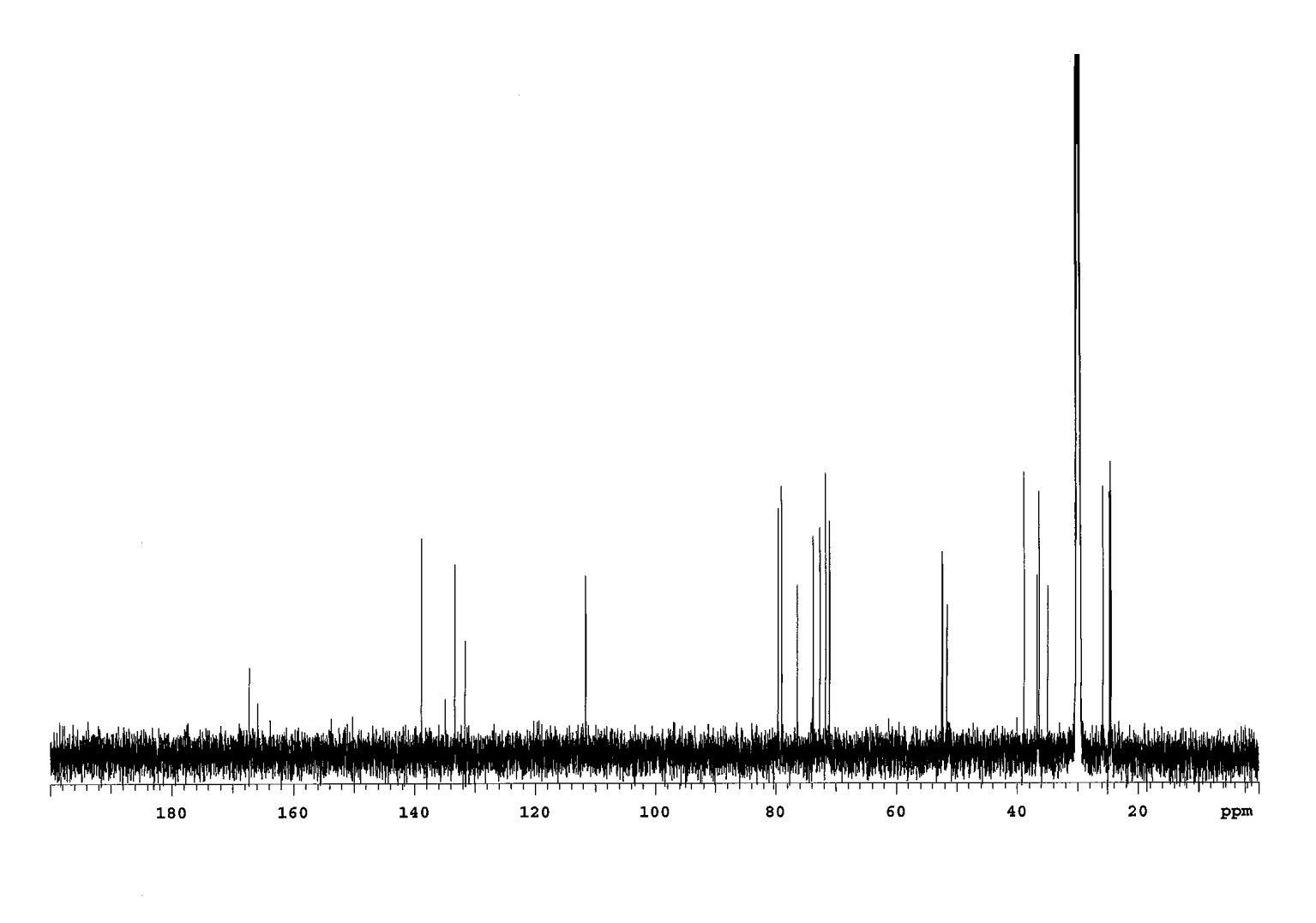 |
| --- |
| 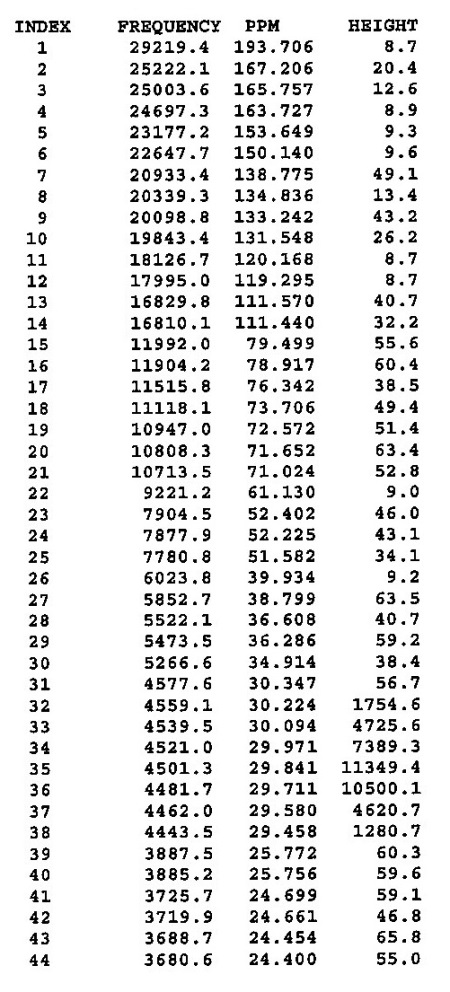 |

**Figure S12.** ^13^C-NMR spectrum of compound (+)-**8** in acetone-*d*_6_ (150 MHz).

| 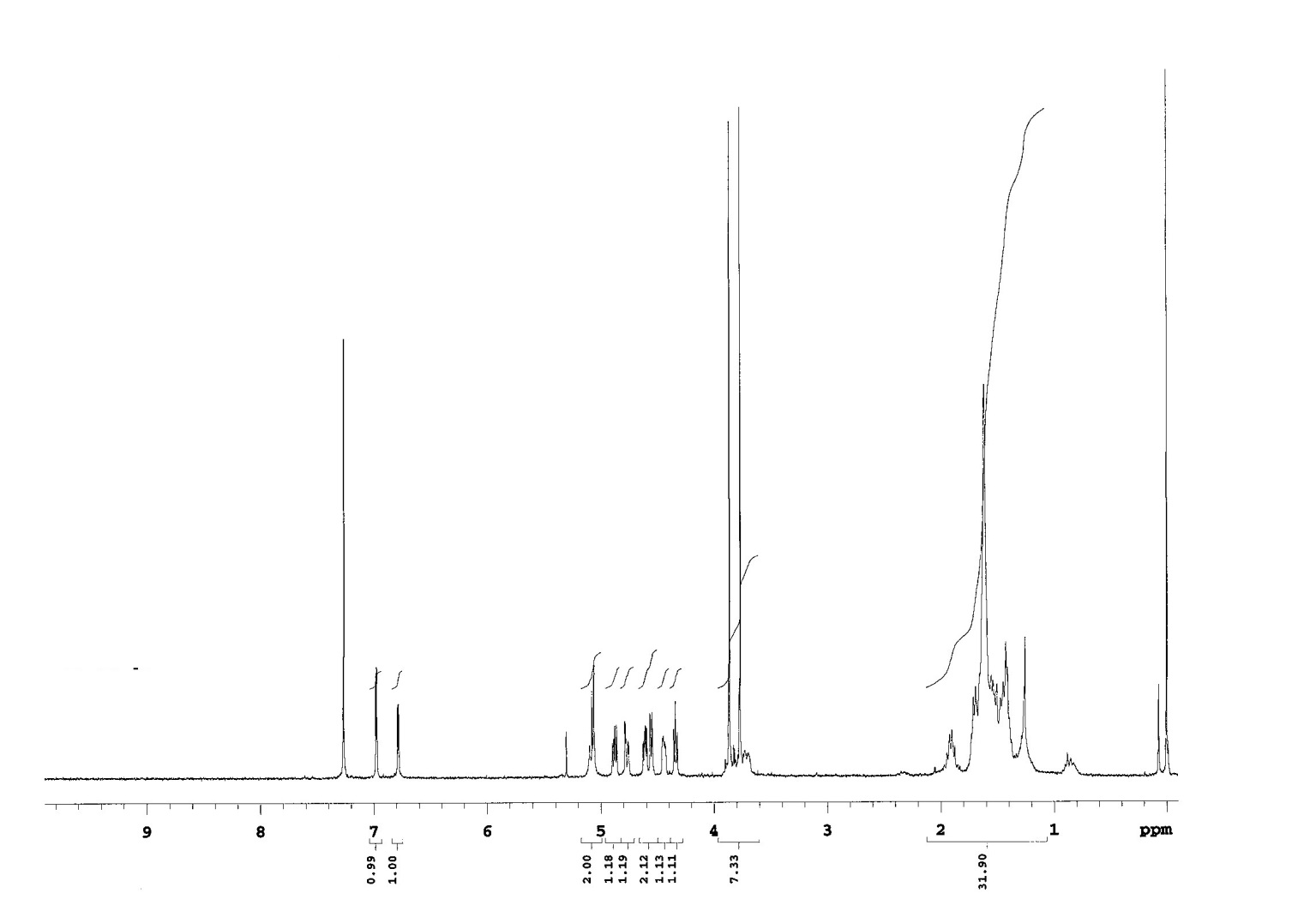 |
| --- |
| 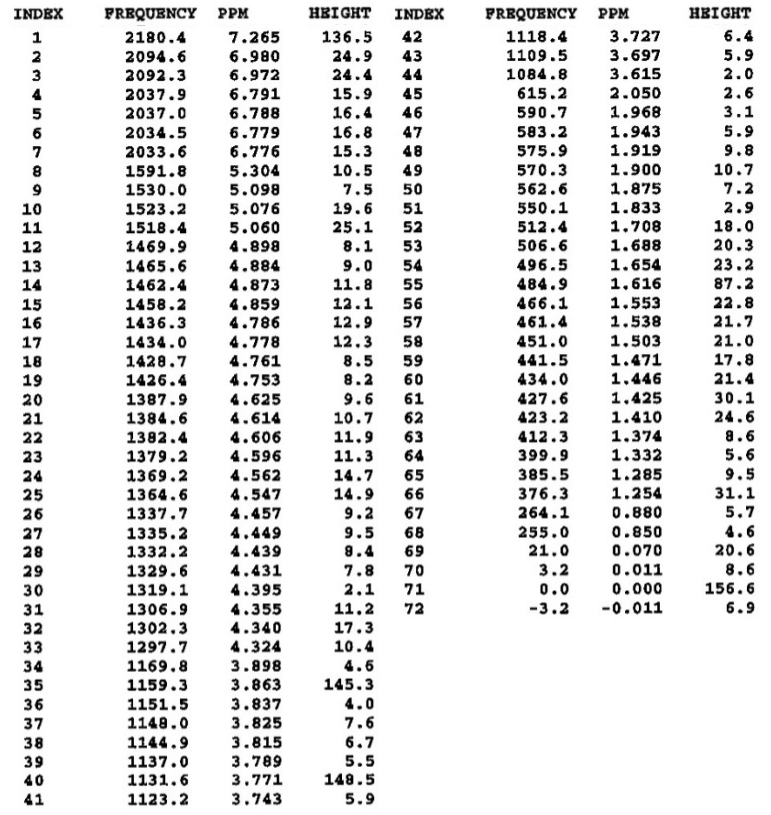 |

**Figure S13.** ^1^H-NMR spectrum of compound (+)-**20** in CDCl_3_ (300 MHz).


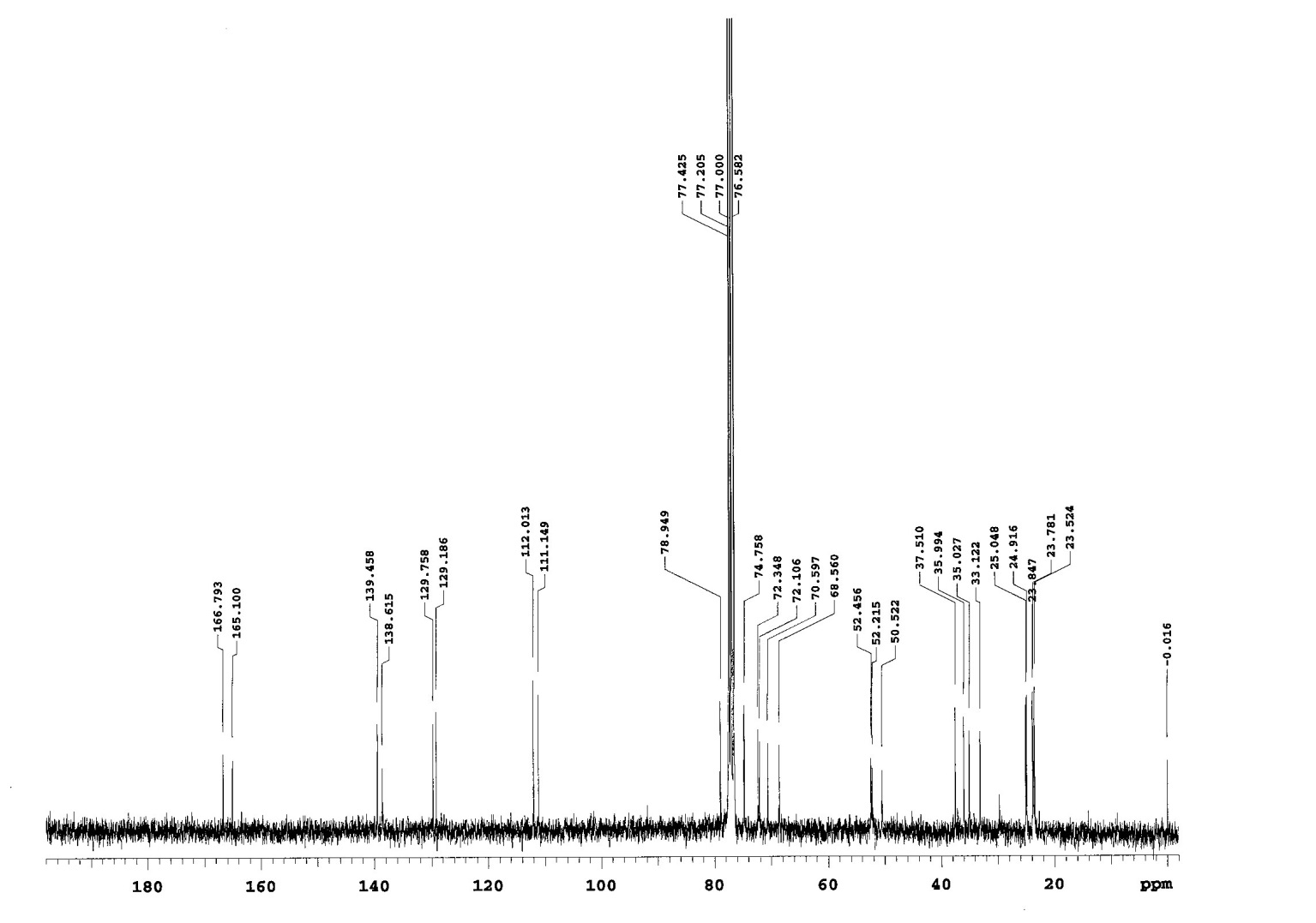


**Figure S14.** ^13^C-NMR spectrum of compound (+)-**20** in CDCl_3_ (75 MHz).

| 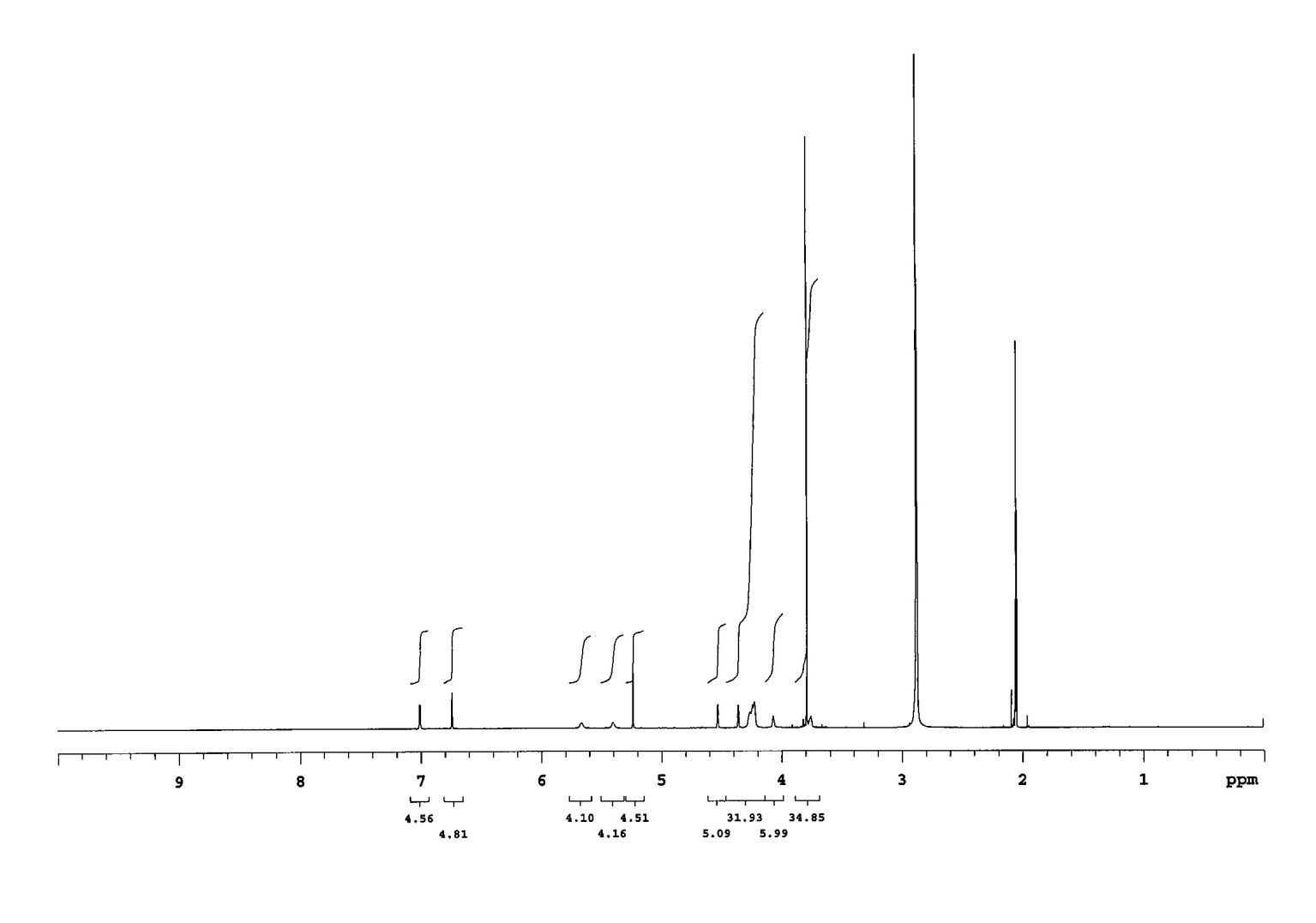 |
| --- |
| 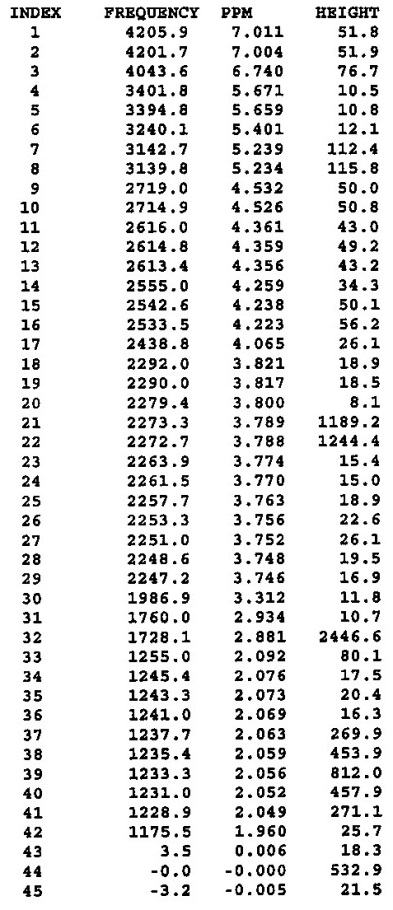 |

**Figure S15.** ^1^H-NMR spectrum of compound (+)-**6** in acetone-*d*_6_ (600 MHz).

| 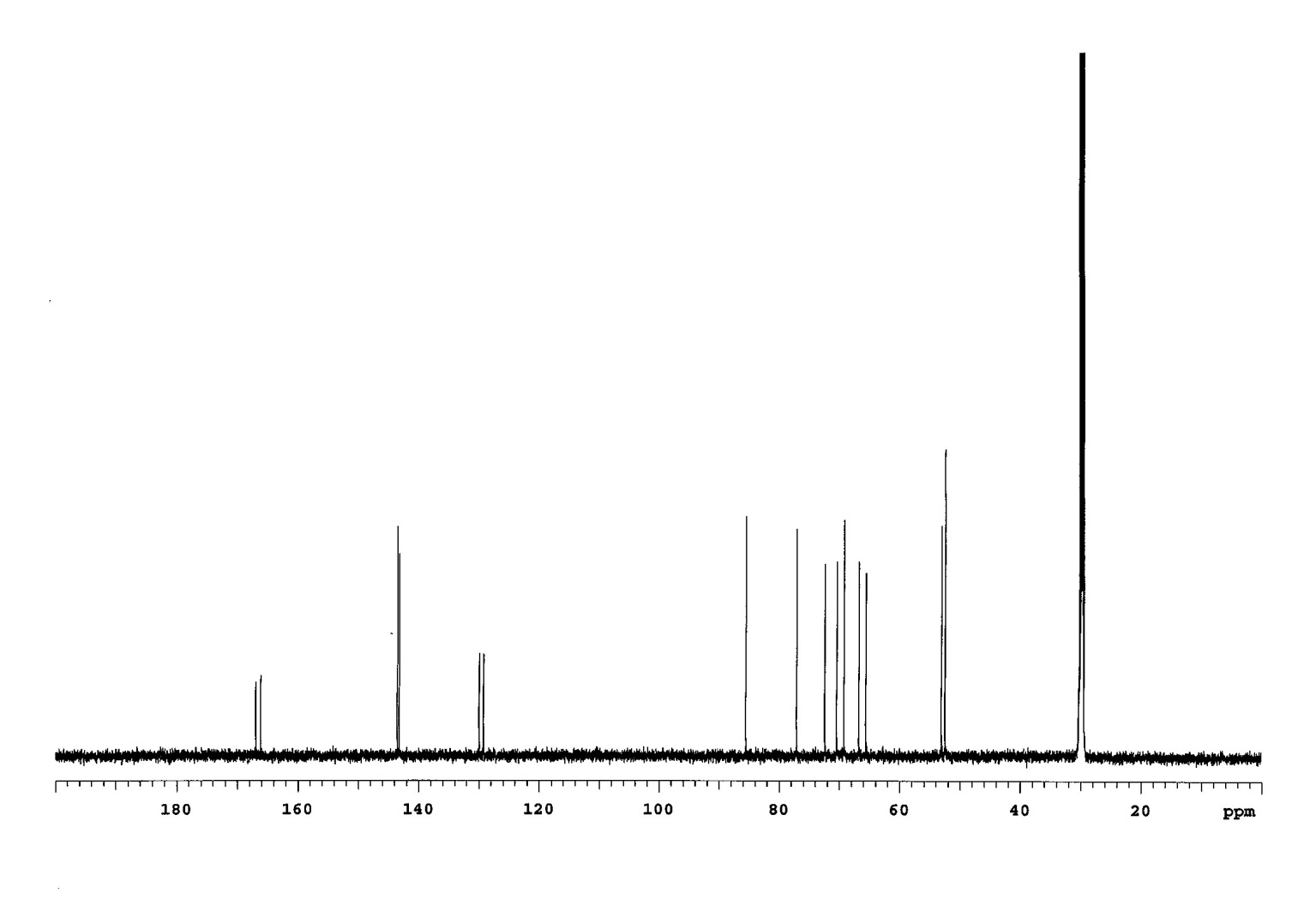 |
| --- |
| 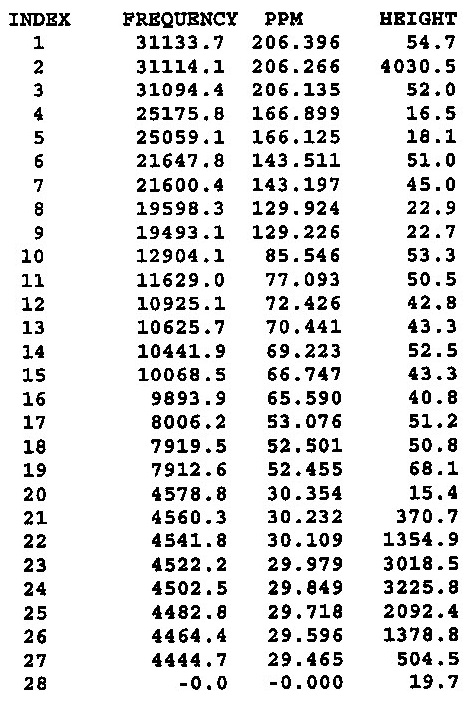 |

**Figure S16.** ^13^C-NMR spectrum of compound (+)-**6** in acetone-*d*_6_ (150 MHz).

| 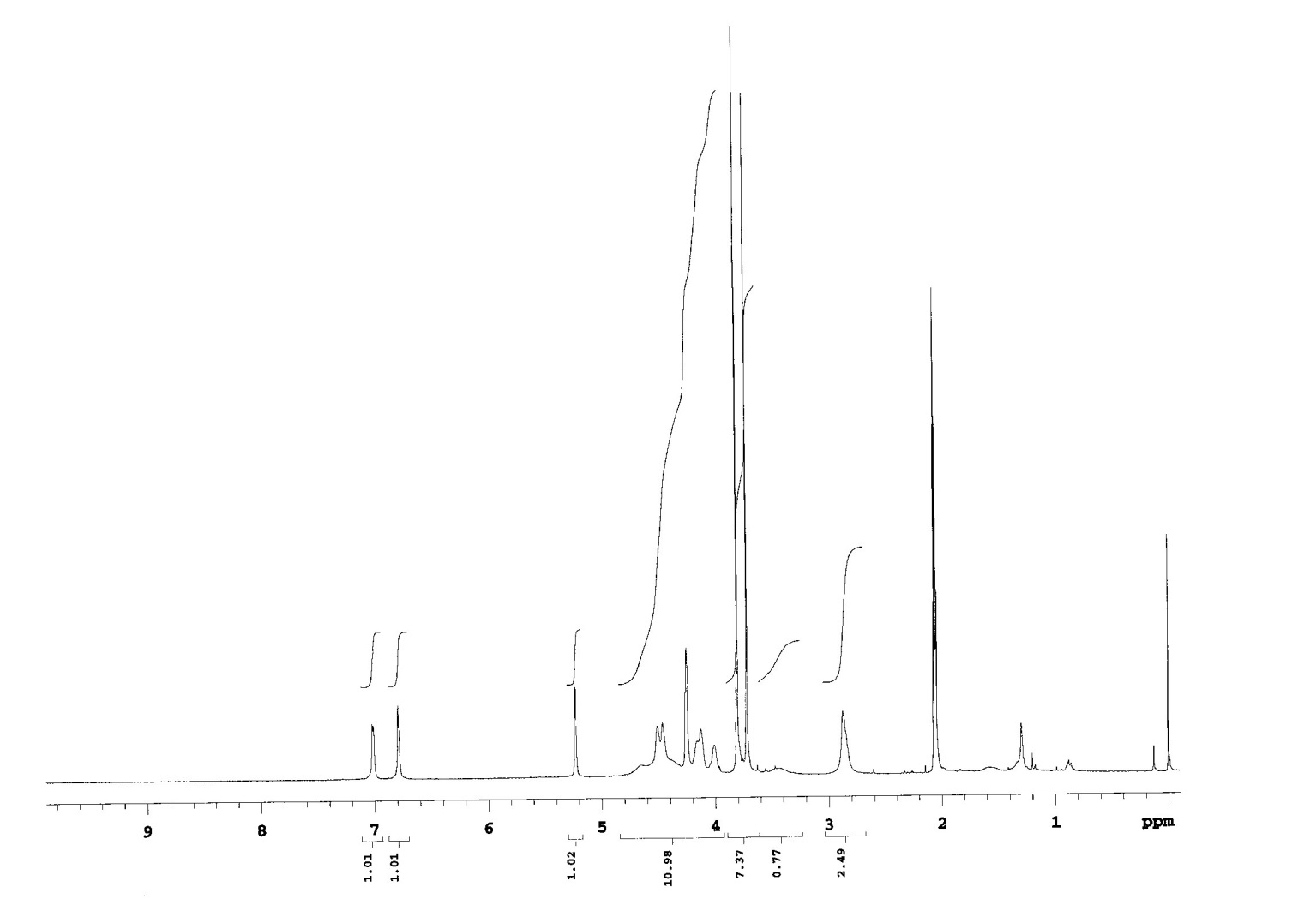 |
| --- |
| 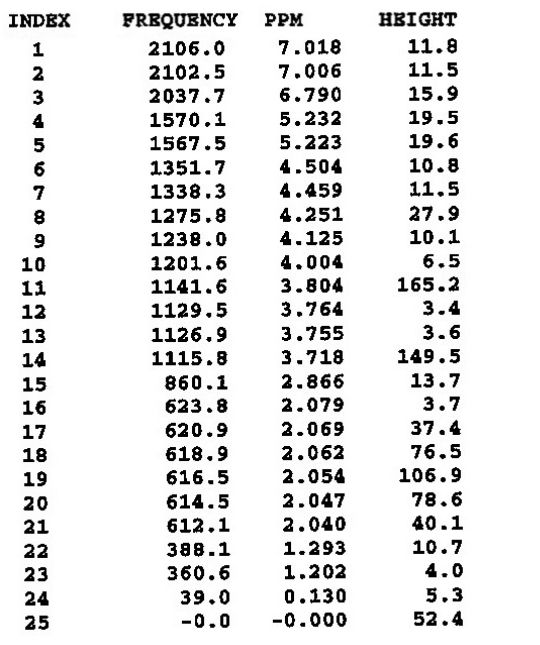 |

**Figure S17.** ^1^H-NMR spectrum of compound (+)-**21** in acetone-*d*_6_ (300 MHz).

| 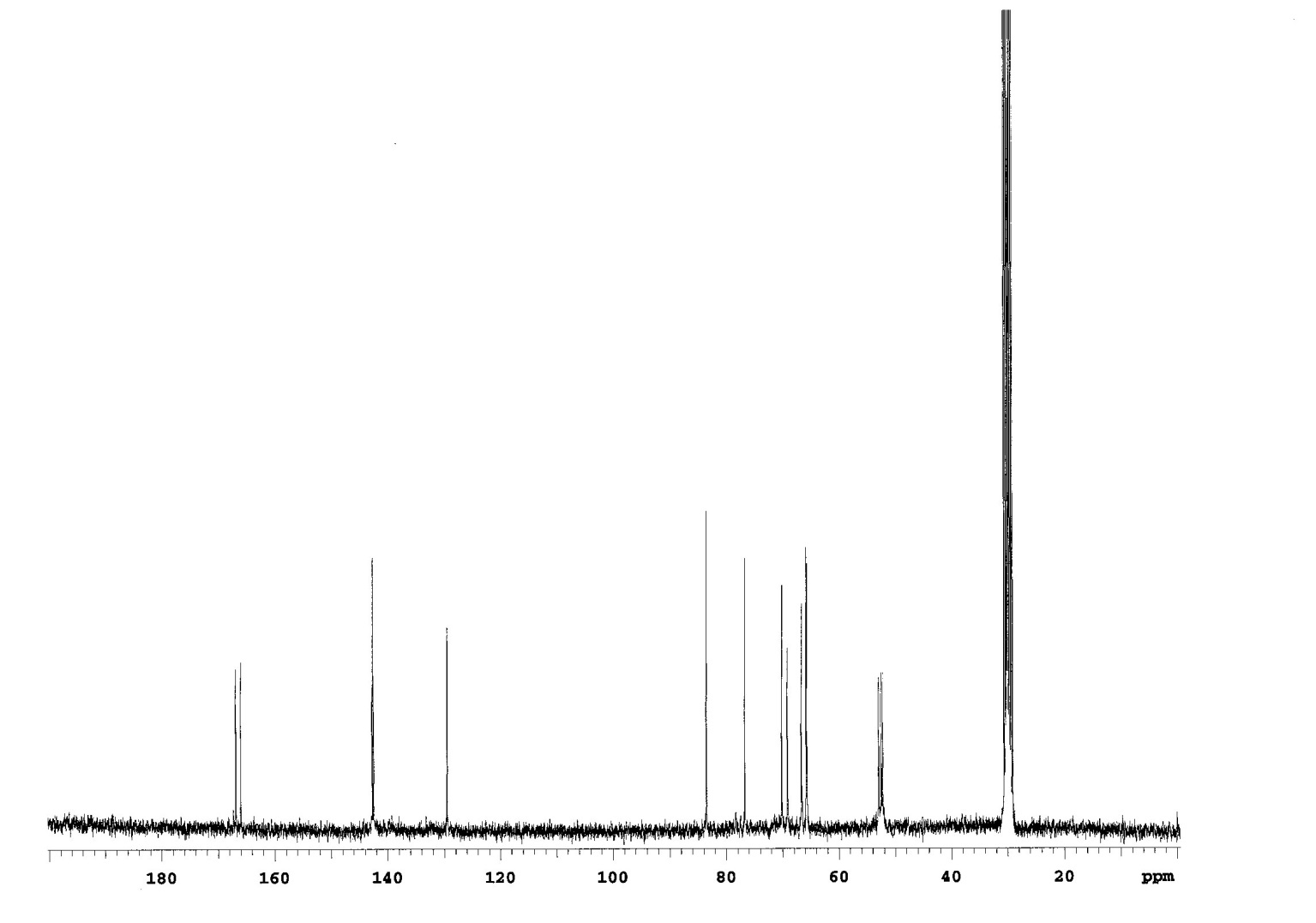 |
| --- |
| 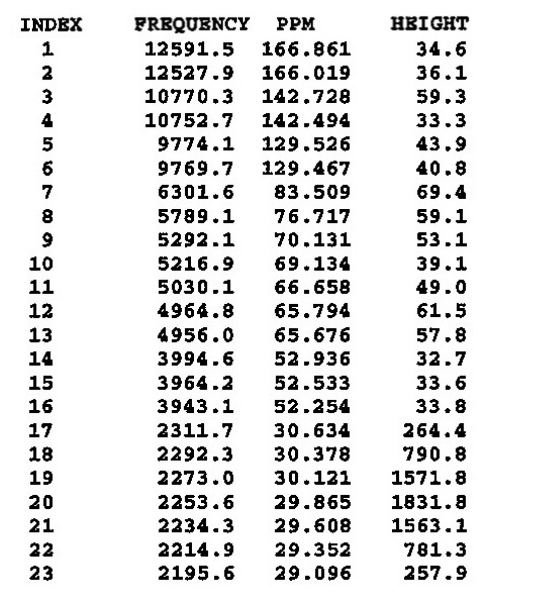 |

**Figure S18.** ^13^C-NMR spectrum of compound (+)-**21** in acetone-*d*_6_ (75 MHz).

| 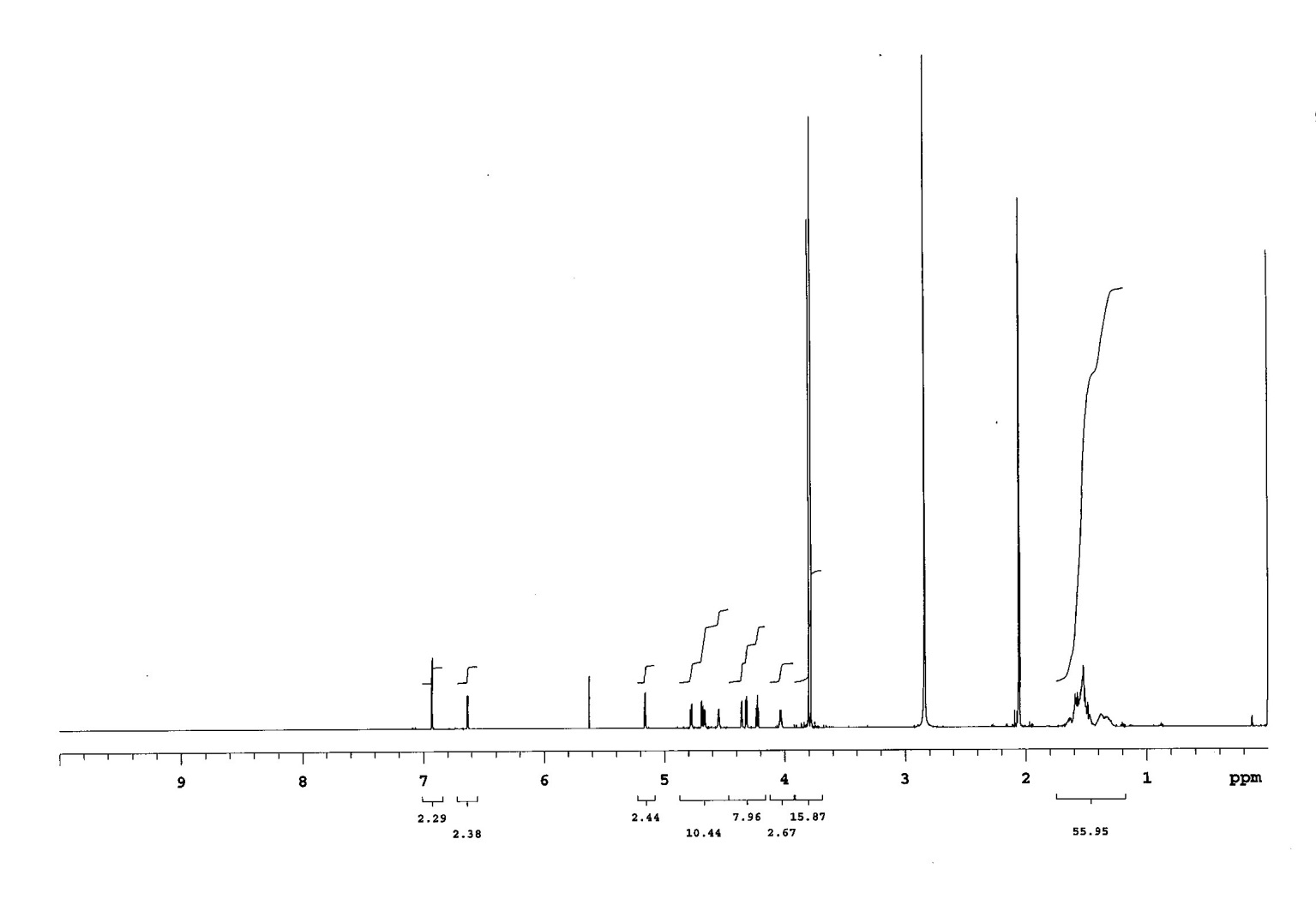 |
| --- |
| 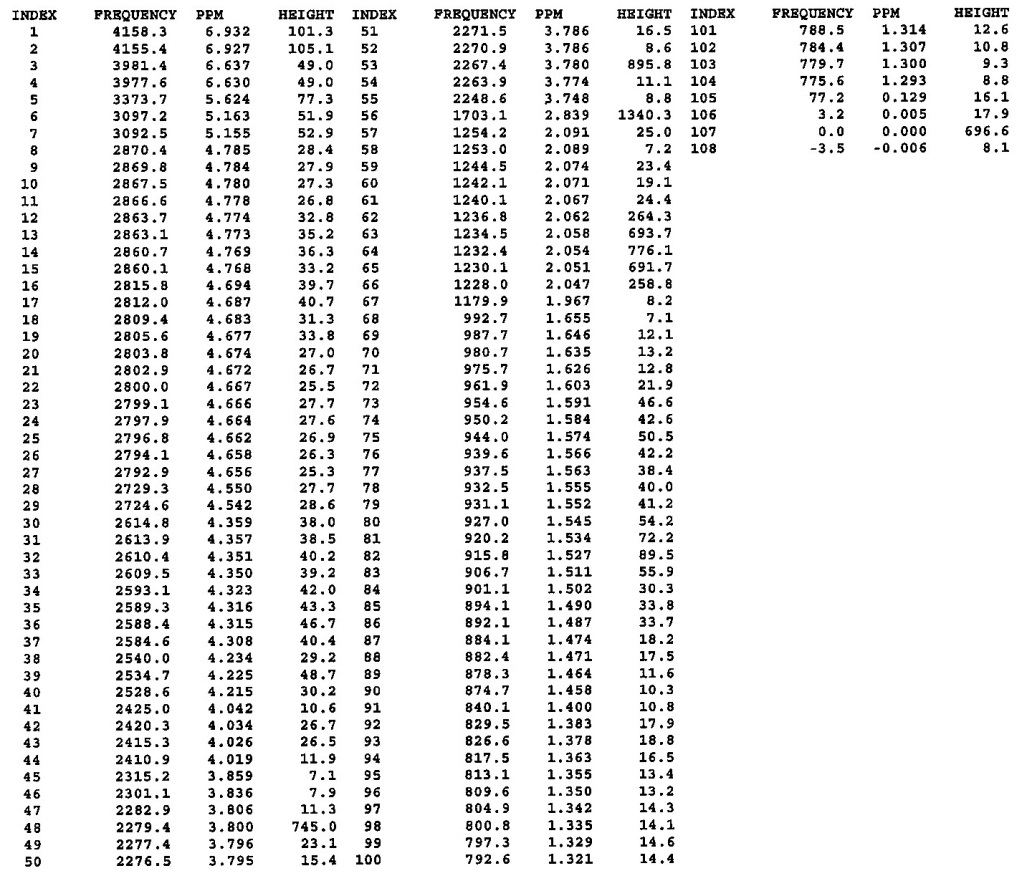 |

**Figure S19.** ^1^H-NMR spectrum of compound (−)-**24** in acetone-*d*_6_ (600 MHz).

| 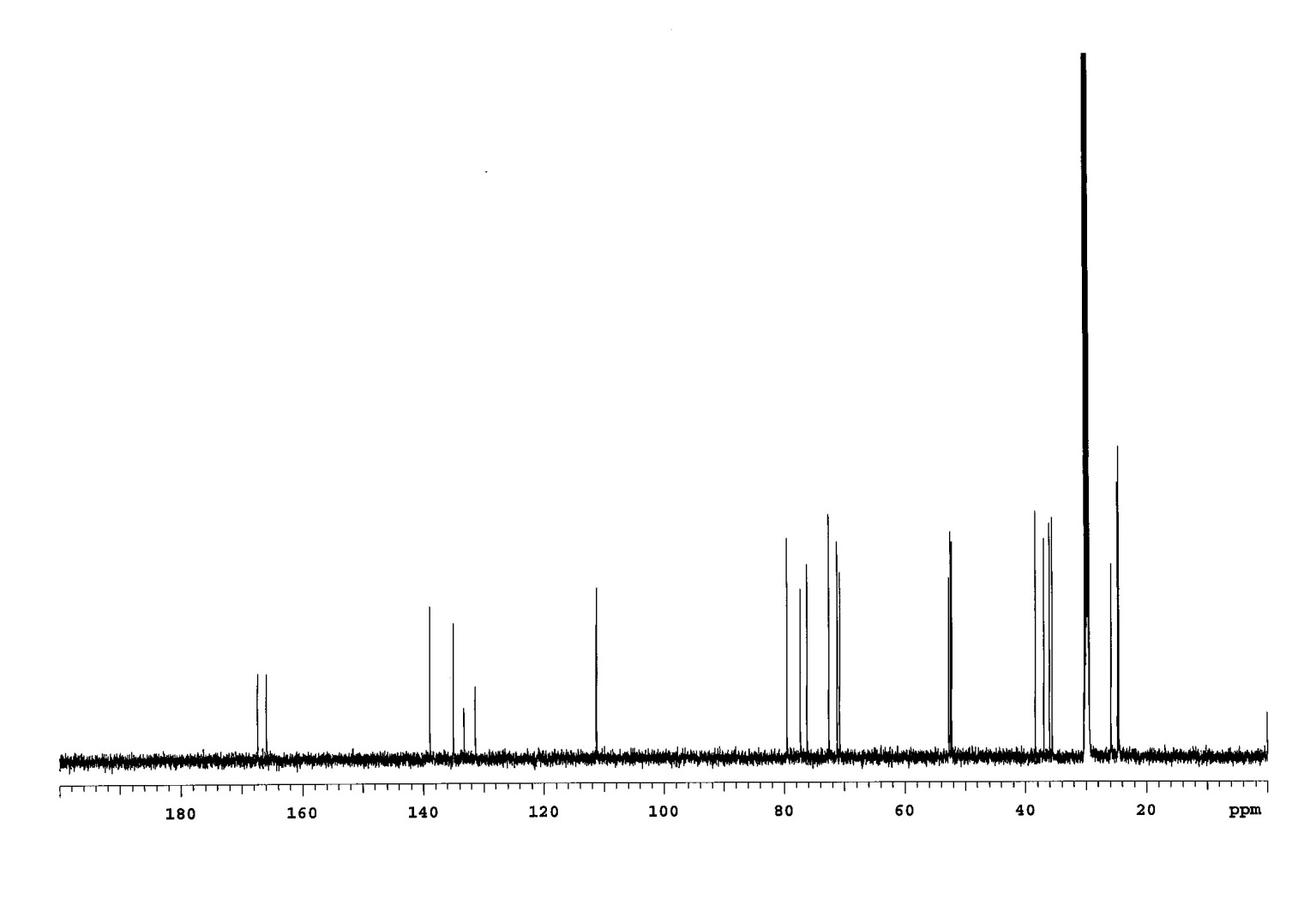 |
| --- |
| 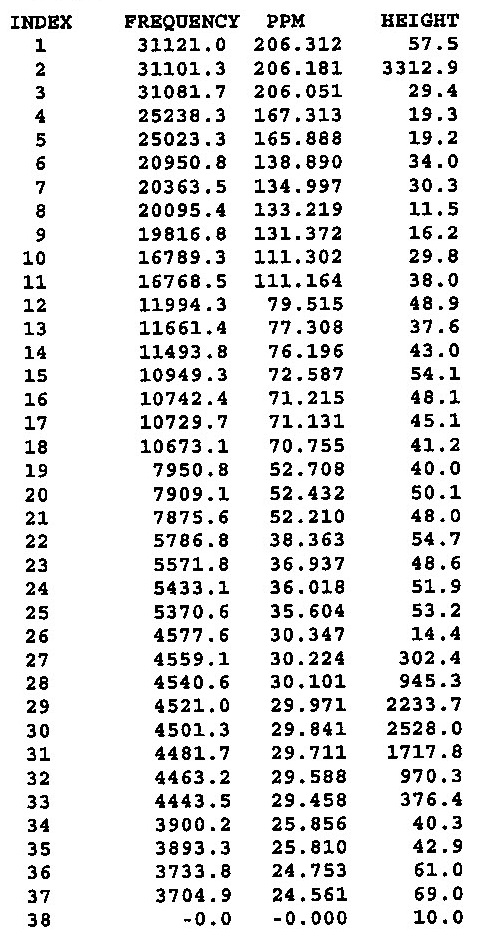 |

**Figure S20.** ^13^C-NMR spectrum of compound (−)-**24** in acetone-*d*_6_ (150 MHz).

| 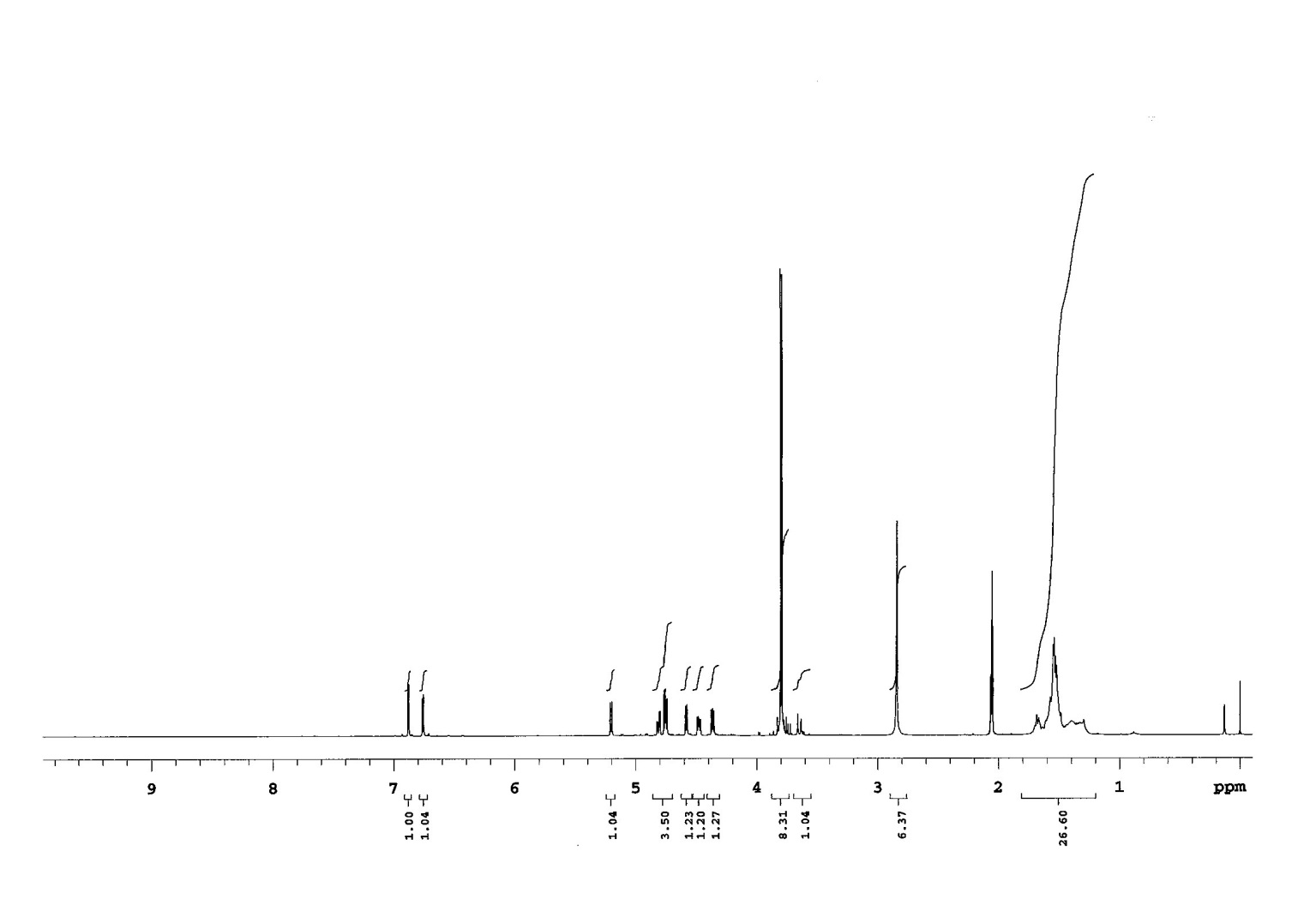 |
| --- |
| 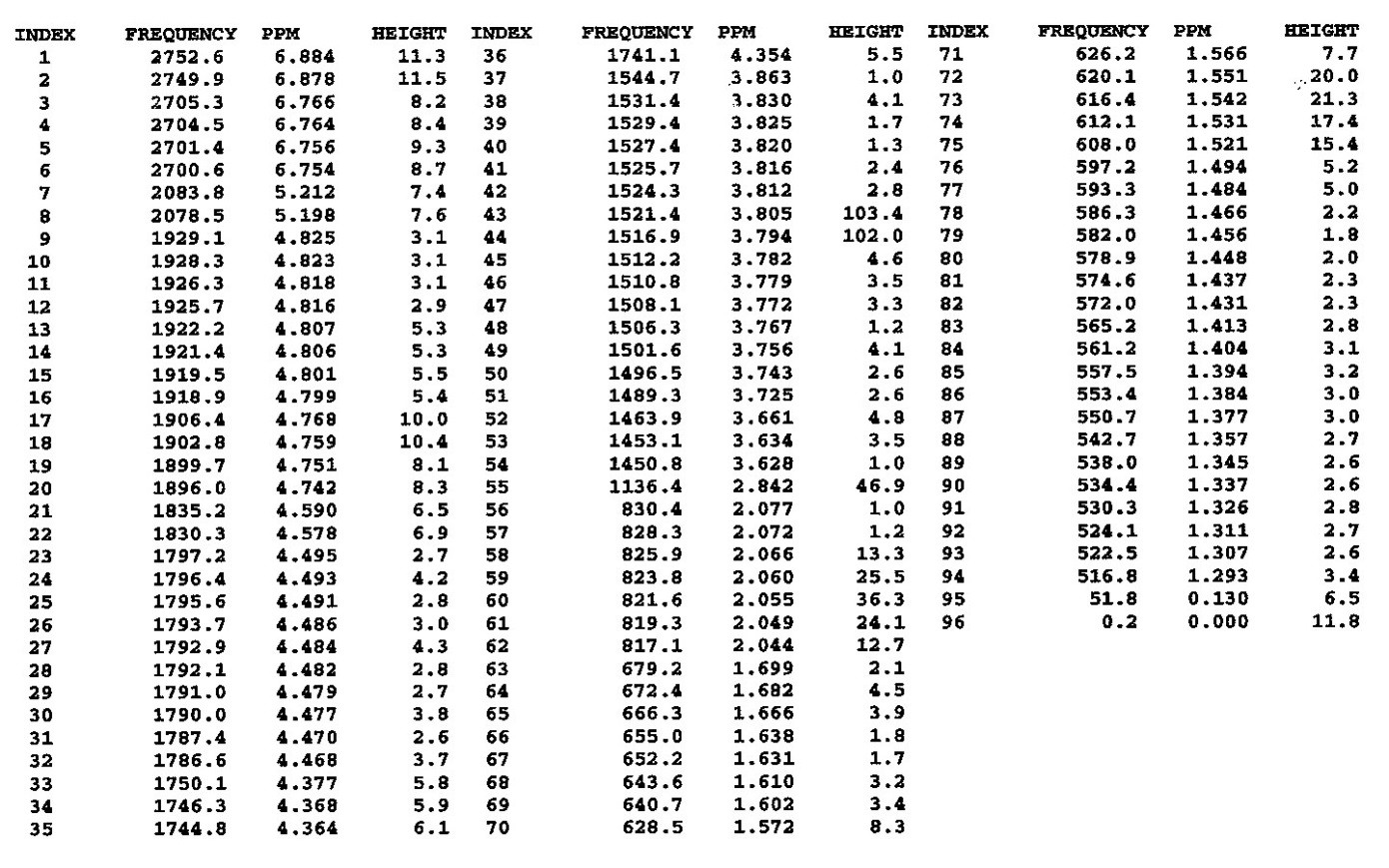 |

**Figure S21.** ^1^H-NMR spectrum of compound (−)-**25** in acetone-*d*_6_ (600 MHz).

| 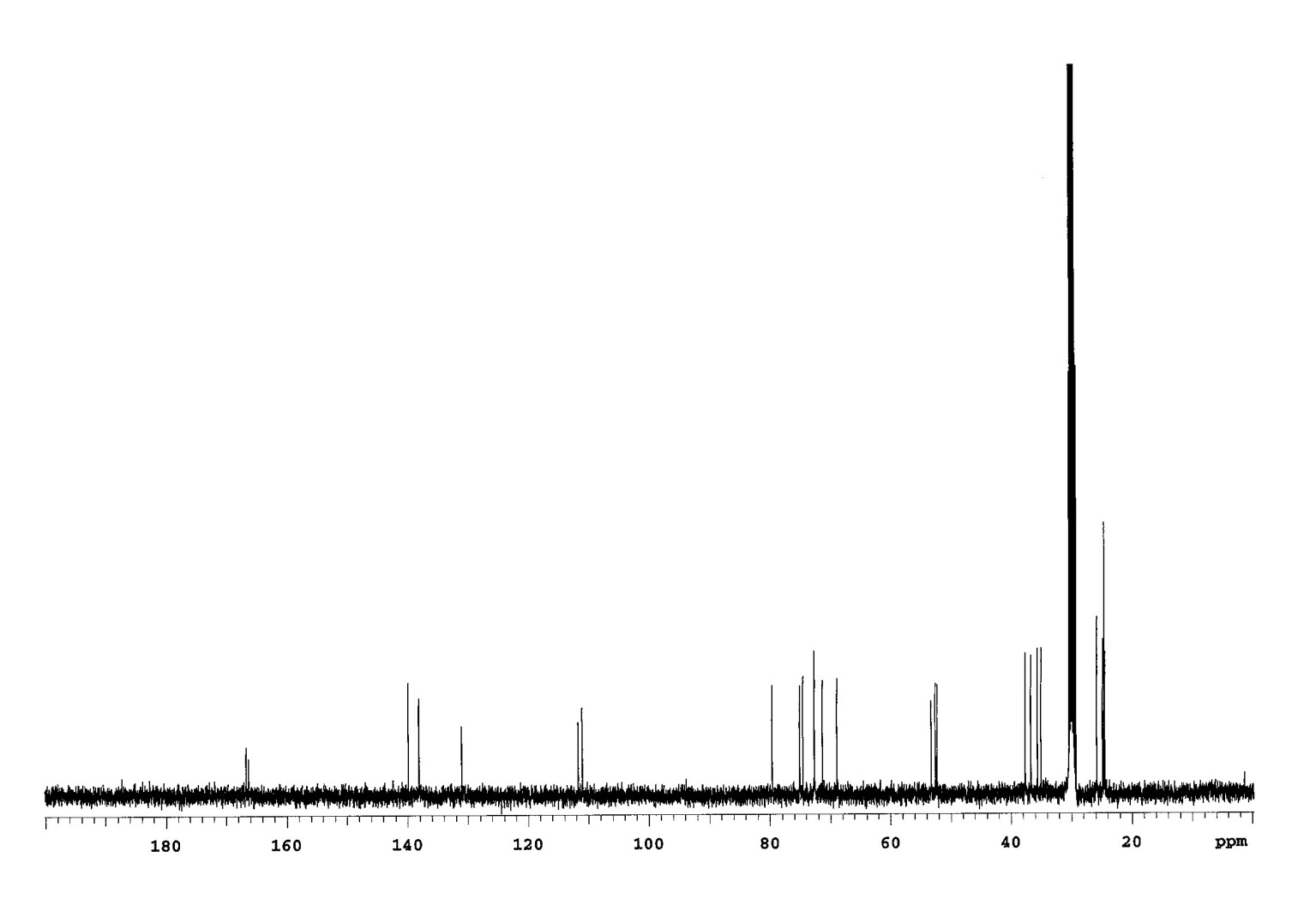 |
| --- |
| 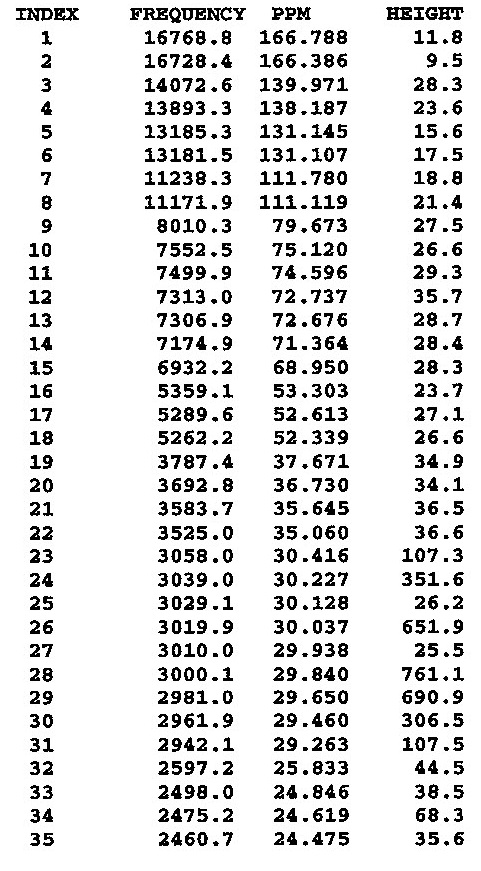 |

**Figure S22.** ^13^C-NMR spectrum of compound (−)-**25** in acetone-*d*_6_ (150 MHz).

| 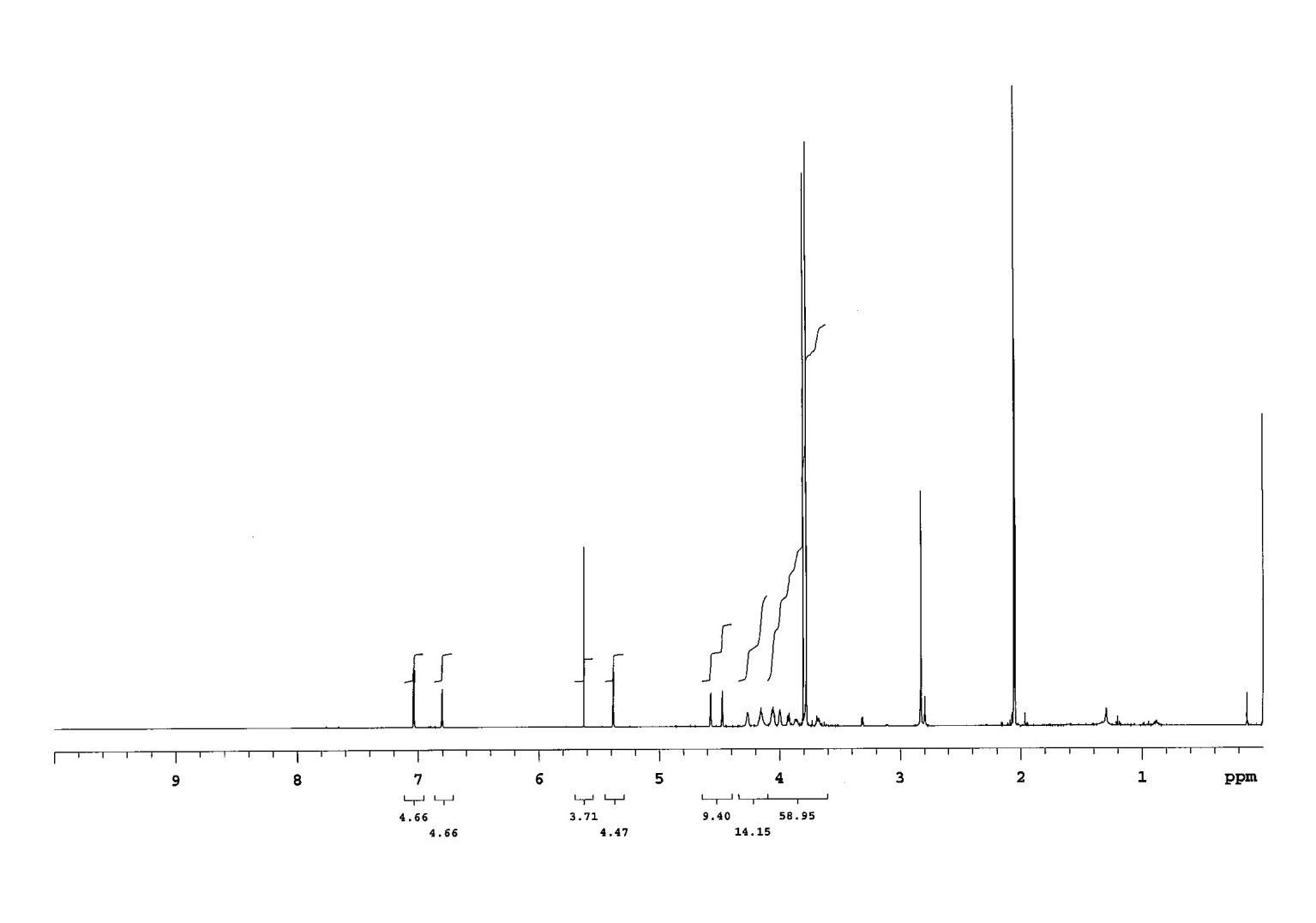 |
| --- |
| 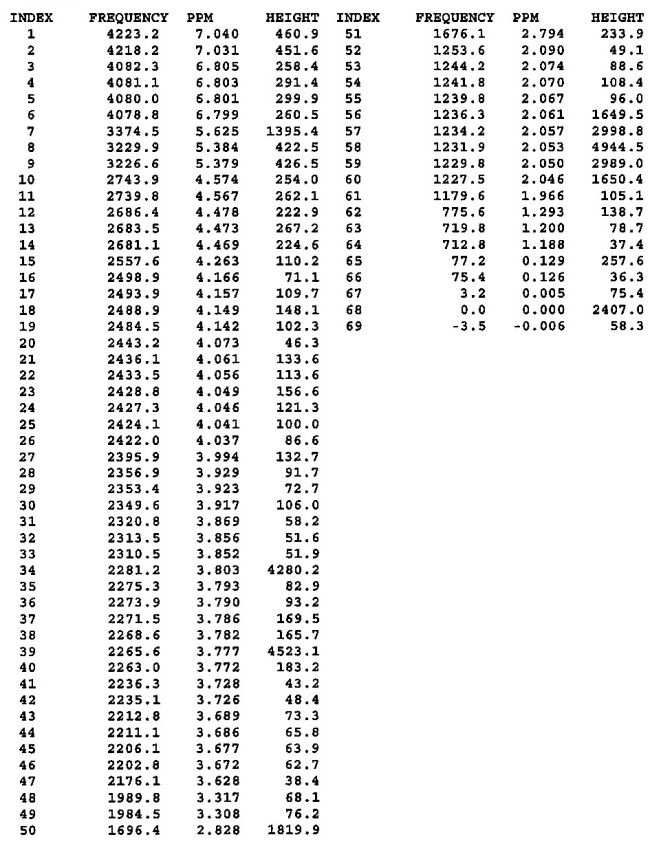 |

**Figure S23.** ^1^H-NMR spectrum of compound (−)-**22** in acetone-*d*_6_ (600 MHz).

| 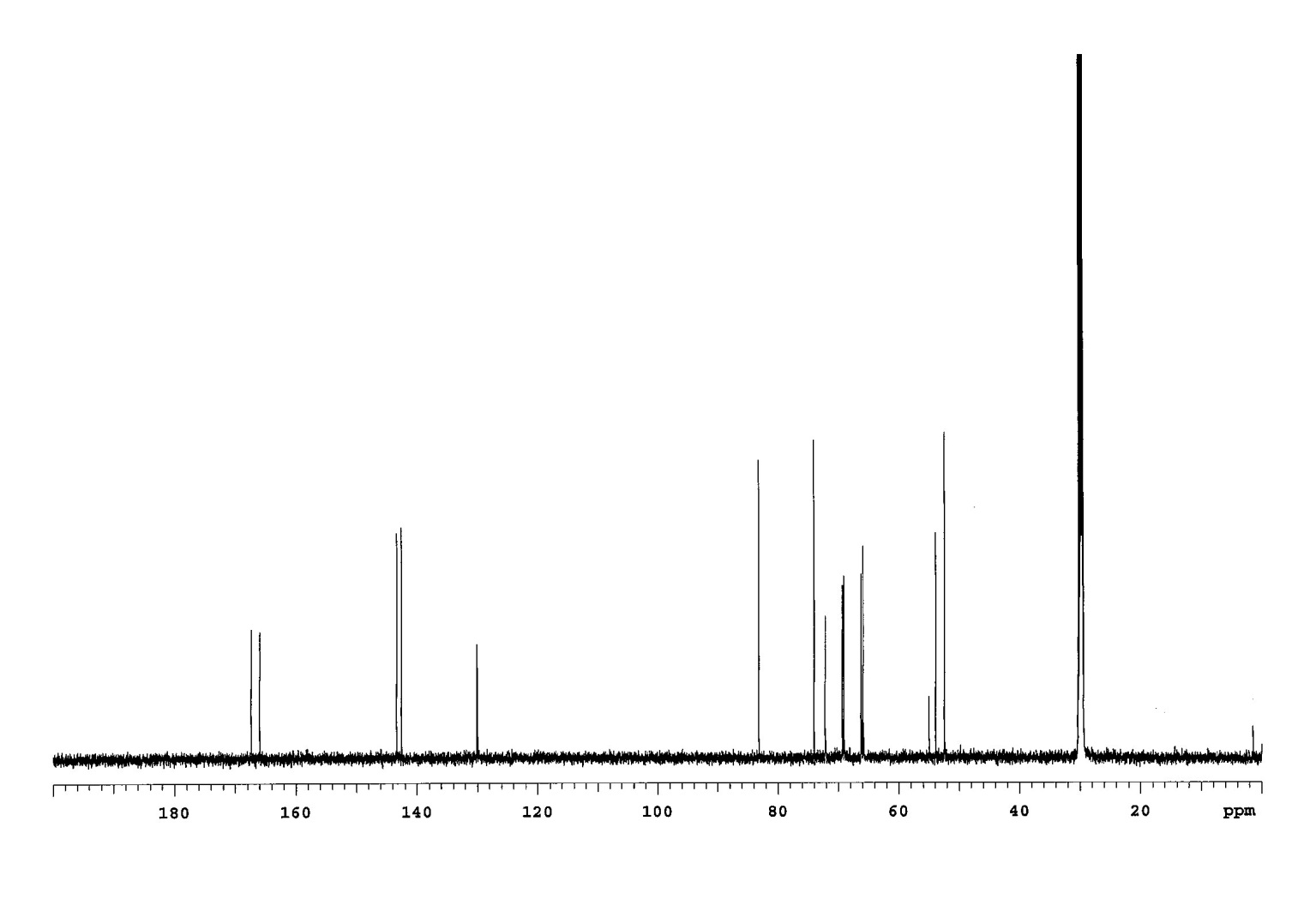 |
| --- |
| 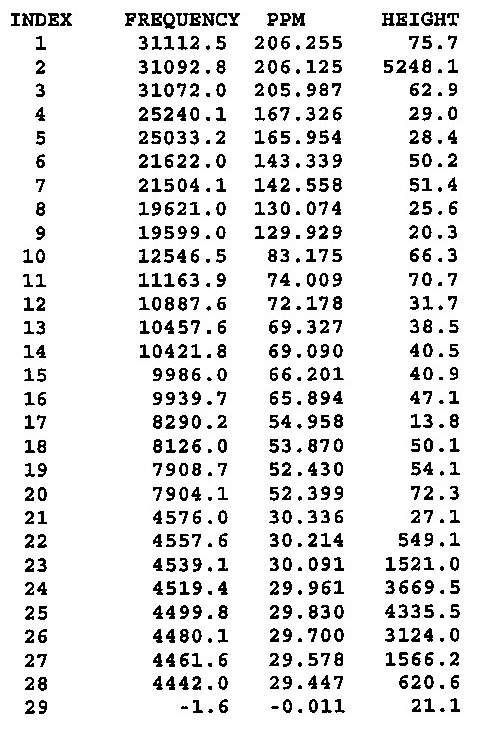 |

**Figure S24.** ^13^C-NMR spectrum of compound (−)**-22** in acetone-*d*_6_ (150 MHz).

| 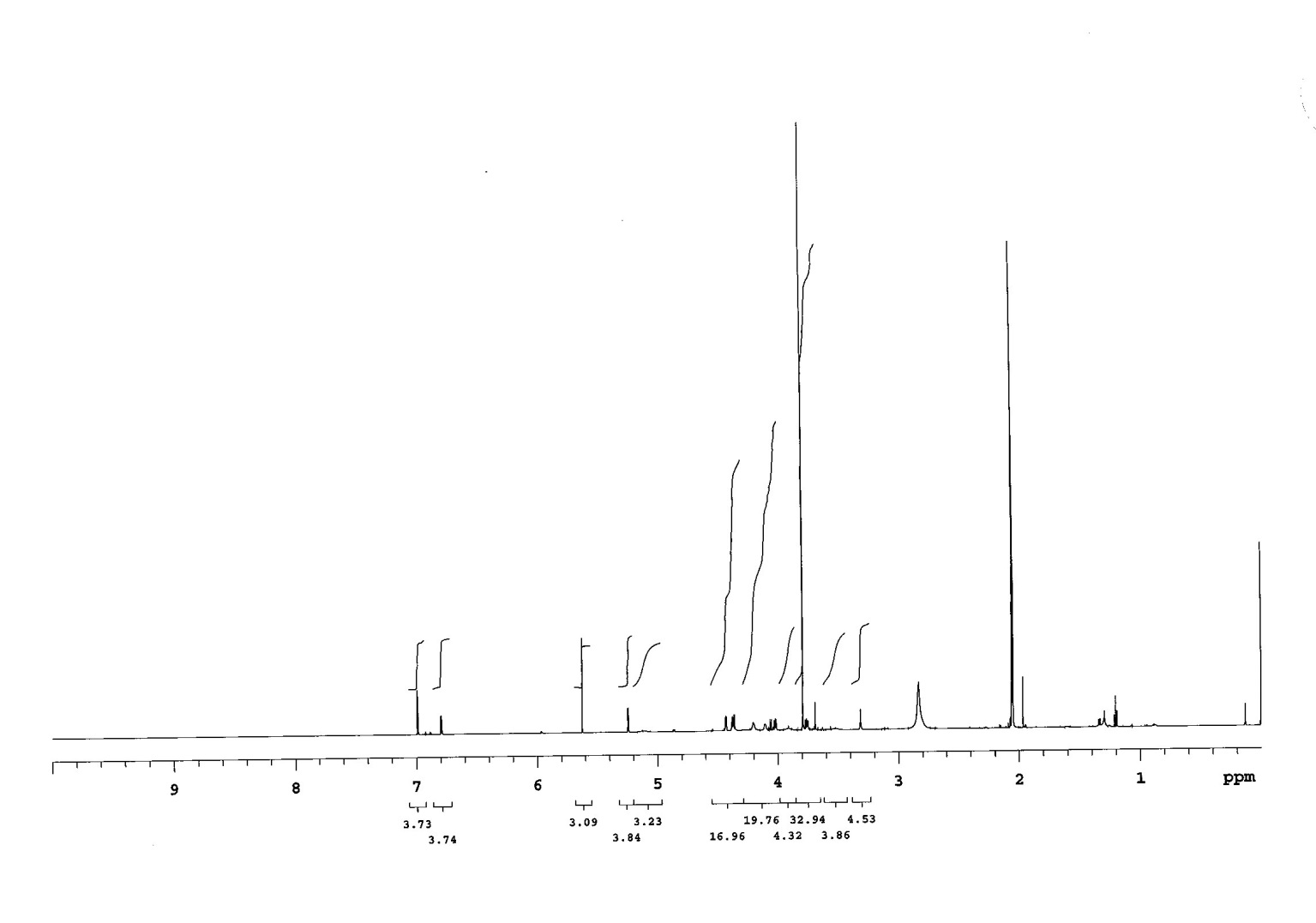 |
| --- |
| 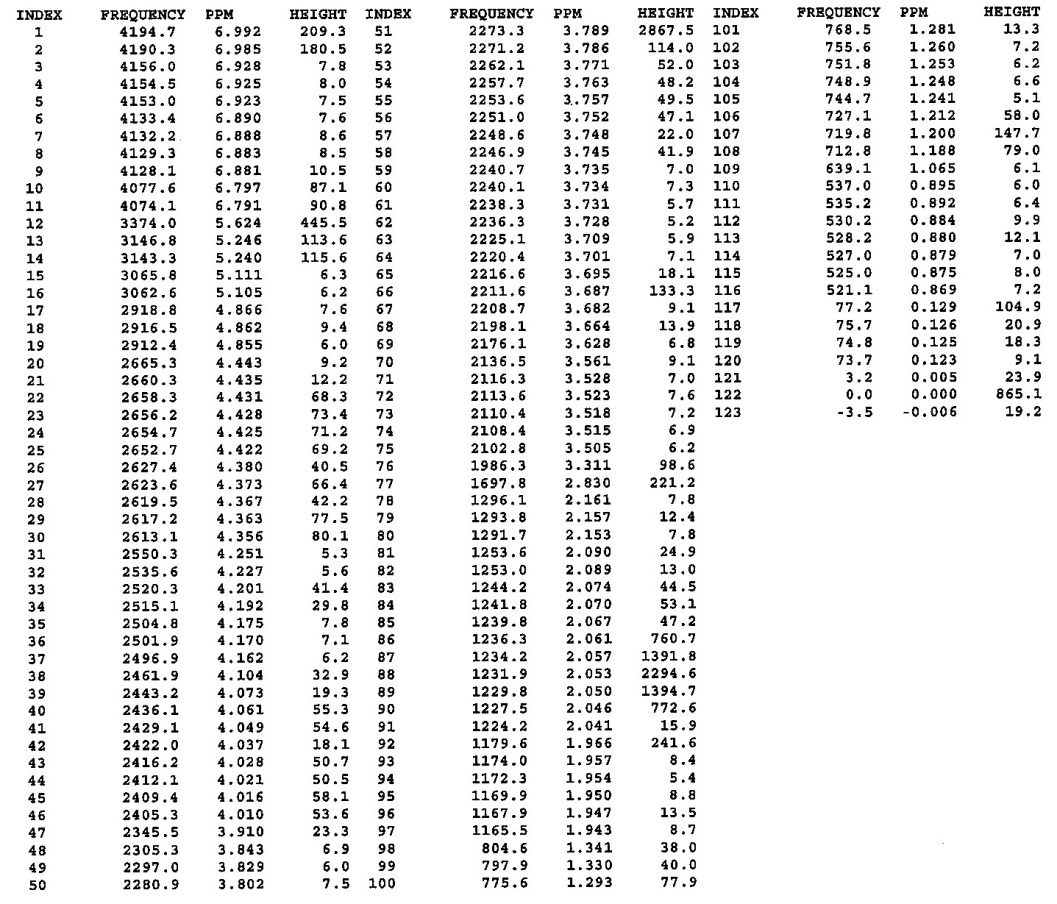 |

**Figure S25.** ^1^H-NMR spectrum of compound (−)-**23** in acetone-*d*_6_ (600 MHz).

| 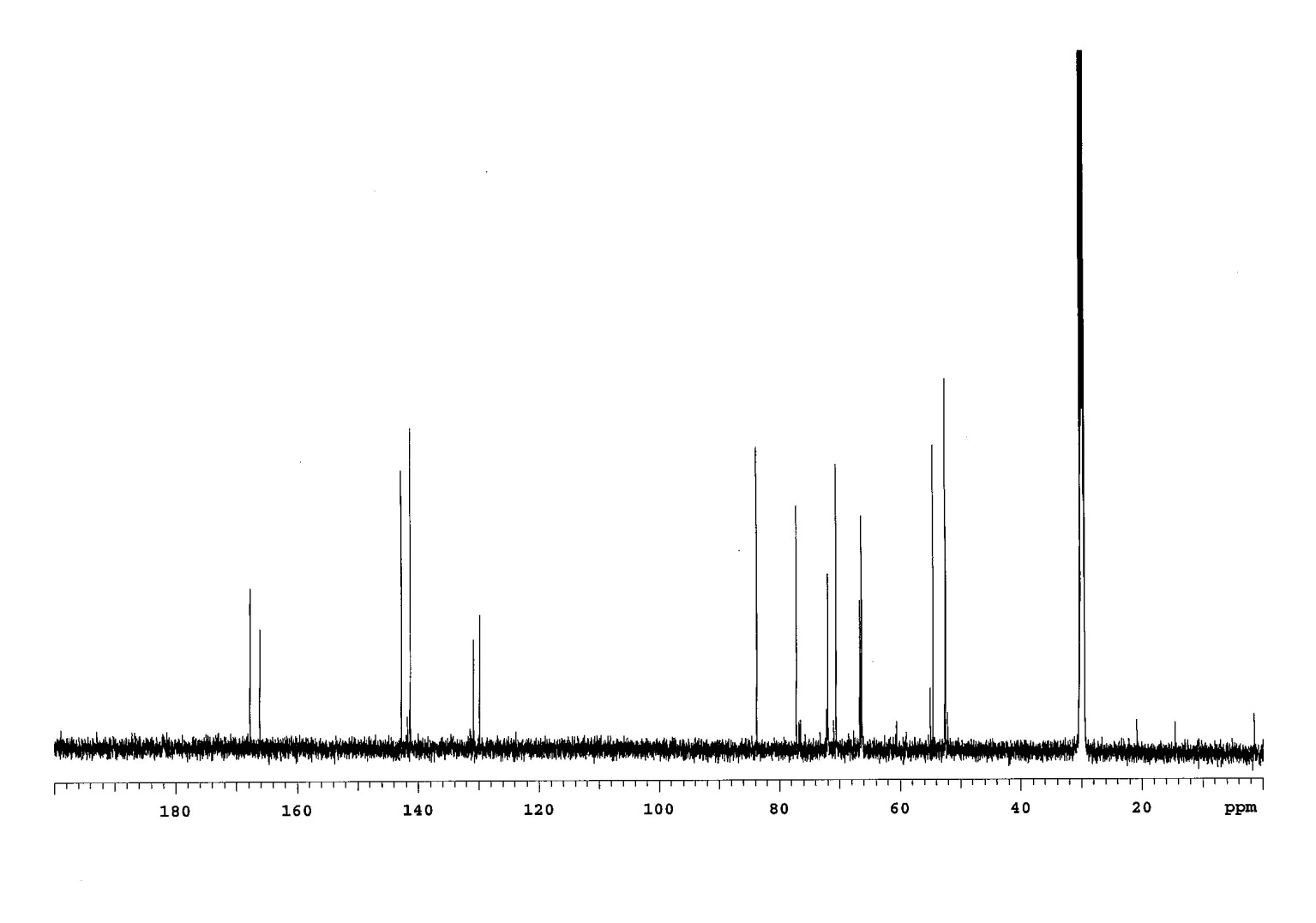 |
| --- |
| 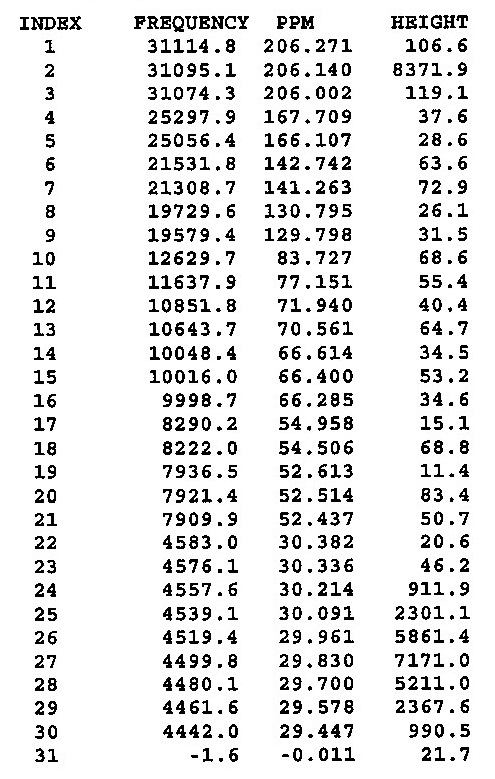 |

**Figure S26.** ^13^C-NMR spectrum of compound (−)-**23** in acetone-*d*_6_ (150 MHz).
